# Metabarcoding confirms the opportunistic foraging behaviour of Atlantic bluefin tuna and reveals the importance of gelatinous prey

**DOI:** 10.7717/peerj.11757

**Published:** 2021-08-13

**Authors:** Babett Günther, Jean-Marc Fromentin, Luisa Metral, Sophie Arnaud-Haond

**Affiliations:** MARBEC, Univ Montpellier, CNRS, Ifremer, IRD, Sète, France

**Keywords:** COI, 18S, Stomach content, Mediterranean Sea, Atlantic bluefin tuna, Diet, Top predator

## Abstract

Studies of the diet, feeding habits and trophic activity of top marine predators are essential for understanding their trophodynamics. The main direct method used for such studies thus far has been morphological inventories of stomach contents. This approach presents limitations such as missing gelatinous prey, which are usually digested too quickly to be detectable. Here, we analysed the stomachs of 48 Atlantic bluefin tuna (*Thunnus thynnus,* approximately 15 to 60 kg, including juveniles and adult fishes) collected from the Mediterranean Sea through the metabarcoding of two gene regions (cytochrome *c* oxidase subunit I (COI) and the ribosomal 18S-V1V2 region). The identified prey taxa and their relative read abundances (RRAs) estimated using COI results were in line with the findings of morphologically based inventories simultaneously performed on the same set of tuna samples. In both cases (and with the same rankings), the prey taxa included anchovy (*Engraulis encrasicolus,* here detected in more than 80% of samples, RRA = 43%), sardine (*Sardina pilchardus,* also approximately 80%, RRA = 30%), sprat (*Sprattus sprattus,* approximately 66%, RRA = 8%), mackerel (*Scomber colias,* approximately 44%, RRA = 7%) and cephalopods (approximately 15%, RRA = 1.4%). Another striking result was the detection, based on 18S (with which vertebrates were detected as the most abundant group, RRA = 61.6%), of a high prevalence and diversity of gelatinous organisms (RRA = 27.1%), including cnidarians (6.7%), salps (11.7%), and ctenophores (8.7%), the latter increasing with the size of the predator. These results thus support the hypothesis of the role of gelatinous prey in the diet of Atlantic bluefin tuna, suggesting that this species is even more generalist and opportunistic than previously thought. This study further confirms that DNA metabarcoding can be a powerful tool for assessing the diet and trophodynamics of top marine predators.

## Introduction

Large pelagic fishes are top predators that contribute to the stability and persistence of marine ecosystems through top-down control (Estes et al., 2011; Hughes et al., 2013). Information about their ecological niche and trophic dynamics, or “trophodynamics” (the spatial and temporal dynamics of trophic interactions, (Lindeman, 1942; Young et al., 2015), provides insight into essential elements of their basic biology. It is also increasingly necessary to understand and forecast the cascading effects of environmental and anthropogenic changes on the marine ecosystems to which they contribute (Myers et al., 2007; Casini et al., 2012).

Several methods can be used to identify the diets of these fish: direct morphological inventories of stomach contents, indirect reconstruction through the biochemical analysis of tissue, and indirect assessment through telemetry surveys (Young et al., 2015; Silveira da et al., 2020). Direct assessment through the analysis of stomach contents is an important step to achieve a high taxonomic resolution (to the species level) and a to-some-degree quantitative assessment of the detailed diet (as opposed to most biochemical marker analyses, which integrate various time steps and are subject to a hypothesis and baseline data underlying their indirect interpretation). However, the morphological identification of prey and/or their remains is time consuming, requires particular skills and experience and, importantly, can be limited by the differential digestion of prey, with species composed of soft tissues often being missed.

As an alternative to direct observation and to circumvent these biases, the use of genetics to inventory fish stomach contents was initiated nearly 20 years ago (Rosel & Kocher, 2002; Symondson, 2002). Currently, the modern tools of metabarcoding combine sequence-based identification with high-throughput sequencing technology (HTS; Pompanon et al., 2012), allowing the inventory of a broad range of taxonomic levels at the same time. Here, two approaches can be applied: the shotgun sequencing of genomic DNA extracted from the stomach or faeces followed by bioinformatic reconstruction or the construction of libraries for the metabarcoding of a target ‘barcode’ gene (Leray et al., 2013b; Berry et al., 2015). While the shotgun method delivers results that may theoretically allow semi-quantitative estimates to be obtained, it is characterized by a very low yield of informative sequences due to the preferential sequencing of the host genome (Alberdi et al., 2018). Furthermore, this strategy success depends on deep sequencing, and bioinformatic reconstruction is still time consuming (Young et al., 2015). In contrast, metabarcoding allows a relatively simple and rapid inventory of prey (Shortridge Megan, 2016; Yoon et al., 2017; Komura et al., 2018). However, it is mostly limited to qualitative inventories, as quantitative aspects (abundance, weight, size, and life stages) of consumed prey are not expected to be accurately inferred due to high PCR bias affecting the number of sequences obtained for each detected target (Heupel & Simpfendorfer, 2010; Nielsen et al., 2018). This method has, however, proven to surpass morphological inventories in diverse fishes, including tropical fishes (Matley et al., 2018), Great Lakes fishes (Mychek-Londer, Chaganti & Heath, 2020), invasive lionfish (Harms-Tuohy, Schizas & Appeldoorn, 2016) and stickleback (Jakubavičiute et al., 2017). Its first application to Atlantic bluefin tuna (ABFT, *Thunnus thynnus*) and Pacific bluefin tuna (*Thunnus orientalis*) demonstrated the power to identify fish prey down to the species level, especially for larvae (Kodama et al., 2017; Kodama et al., 2020).

ABFT is an emblematic large pelagic migratory species of high commercial value with a vast geographical distribution (Fromentin & Powers, 2005). ABFT often shows major changes in its spatial distribution, together with long-term fluctuations in catches (Ravier & Fromentin, 2001; Fromentin et al., 2014; Faillettaz et al., 2019). The importance of ABFT as top a predator indicates the need for better knowledge of its biology, including the trophic resources its populations depend on during different stages of the life cycle. While the ABFT adult diet is primarily composed of fish, crustaceans and cephalopods, there have also been records of jellyfish and salps (Fromentin & Powers, 2005), and the consumption of appendicularians (solitary tunicates) has been demonstrated in ABFT larvae before the switch to piscivory (Llopiz et al., 2010). This is not surprising, as stable isotope analysis supports the hypothesis of massive consumption of gelatinous prey, such as cnidarians, ctenophores, and Salpida, among other top marine predators (Cardona et al., 2012; Hays, Doyle & Houghton, 2018). For instance, metabarcoding showed that jellyfish contribute to the diet of several fishes (Lamb et al., 2017) and top predators such as albatross (McInnes et al., 2017) and penguin (Jarman et al., 2013). Nevertheless, there has been no direct evidence or species identification reported thus far to confirm this hypothesis for ABFT (and other large pelagic fish) because the anatomy of such high-water-content prey organisms is very rapidly degraded in stomachs; thus, they are very likely to be overlooked in most morphological inventories (Diaz Briz et al., 2017; Hays, Doyle & Houghton, 2018). Considering the considerable density and large blooms of jellyfish that occur in oceans worldwide and their reported (though debated) increase in recent decades, partially attributed to anthropogenic disturbance and global warming (Condon et al., 2013; Duarte et al., 2013), the confirmation of the contribution of jellyfish to the diets of top predators, such as ABFT, could drastically change our view of trophodynamics in marine ecosystems.

In a previous study aimed at quantifying ABFT predation on sardines and anchovies, Van Beveren et al. (2017) visually analysed prey items in the stomachs of ABFT caught in the northwestern Mediterranean (Gulf of Lions) and mostly identified small pelagic fish and cephalopods. No trace of gelatinous plankton was observed through visual inspection (L. Métral, 2020, pers. comm.). Here, we analysed ABFT stomach contents from the same sampling sets on the basis of two gene regions. The cytochrome *c* oxidase subunit I (COI; Hebert et al., 2003) region presents the highest potential for dietary analyses, combining species-level discrimination for many metazoan groups and the availability of large curated databases (Leray et al., 2013b; Machida et al., 2017). The 18S-V1V2 ribosomal region (Machida & Knowlton, 2012) allowed us to complete the inventory with a broader range of potential metazoan prey, including more invertebrates than are usually captured using COI. We then compared our results with the previously published morphological inventories of prey (Van Beveren et al., 2017) obtained from fishes caught the same three consecutive years.

In this work, we performed an inventory of the stomach contents of bluefin tuna using molecular metabarcoding, focusing on three main objectives. We first aimed to test the accuracy of metabarcoding stomach contents (rather than faeces) according to a presence-absence inventory as well as associated metrics (relative read abundance, RRA) to describe the ABFT diet. Our second objective was to assess the importance of gelatinous species in the ABFT diet and potential differences between ABFT juveniles and adults due to differences in foraging behaviour. Finally, we also tested the usefulness of blocking primers for minimizing predator sequences and optimizing the sequencing depth for prey in molecular analyses of tuna stomach contents.

## Methods

### Sampling

ABFTs were caught in the Gulf of Lions (northwestern Mediterranean Sea, southern France) by local small-scale fishery using longlines or handlines, which recently become the second bluefin tuna fishery in the world to achieve the Marine Stewartship council (MSC) certification (a global standard for sustainable and rather ethical fishing, see https://fisheries.msc.org/). All the stomachs were collected at the landings port (Sète, France) from fish captured between late July and early December of 2011 to 2014. Individual fishes were measured (±1 cm, fork length) and weighed (±0.1 kg) while stomach contents were weighed, and completely and partially intact prey were identified to the lowest possible taxonomic level (Van Beveren et al., 2017). A set of 48 tuna stomachs was equally subsampled regarding weight, size class, time, and even subregion but separately handled for this study by freezing for later genetic processing (see list of samples in Table S1/Appendix 1). The metadata associated with most individuals included the year, sex, weight (15.4–60.5 kg ± 0.1 kg), size class (*J* < 30 kg and *M* > 30 kg) and mouth length (8.8 cm–17 cm), as an indicator of overall length, and these metrics did not vary significantly compared to the sampling set that was used in the morphological study.

### DNA extraction and sequencing

For DNA extraction, the stomachs were only slightly thawed to facilitate the dissociation of stomach contents while maintaining the lowest possible count of host cells and, thus, minimizing contamination by host (tuna) DNA. For every stomach, all laboratory surfaces were cleaned with bleach, and the upper part of the stomach was carefully incised with a scalpel to open the stomach, after which its complete contents were emptied into a (commercial) bleach-disinfected blender, where they were mixed for 30 s to 2 min until completely homogenized. We performed this step on only unfrozen (the thawed tissues were still rigid, and the block had a nearly null temperature) stomach contents to limit the natural heating during blending to a marginal level, in order to limit DNA degradation during this process. Approximately 1 g of the homogenized mixture was then used for DNA extraction. In addition to mechanical maceration, lysis with proteinase K proved necessary, likely due to the high protein content of the mixture of nearly intact fish prey. For extraction, the best results were thus obtained using a lysis step followed by the NucleoSpin Tissue Kit (Macherey-Nagel Düren, Germany). This kit was thus used for all DNA extractions, following the manufacturer’s instructions, except that DNA elution was performed twice in 50 µl of preheated (70 °C) molecular water. Two empty NucleoSpin columns were added to the extraction series and used as extraction controls. PCR was performed separately for two barcoding gene regions (Table 1): COI (Metazoa) and 18S-V1V2 (Metazoa). All primers were synthesized with Illumina adapters and later combined with Illumina barcodes to allow multiplexing. Compared to these original primer sequences, Inosine (I) were changed with “wobbles” (N) to create degenerate primers compatible with the High-Fidelity Phusion Taq Polymerase used in our experiment (which does not recognize Inosine). Each 30 µl amplification reaction contained 4 µl of DNA template, each primer at 0.7 pM, 15 µl of Phusion^®^ High-Fidelity PCR 2X Master Mix with GC buffer (New England Biolabs, Ipswich, MA US), and molecular water to the final volume. Additionally, all COI reactions included an additional 1 mM MgCl_2_ (1.2 ul/25 mM), which together with the 1.5 mM MgCl_2_ contained in the Mix-Phusion resulted in 2.5 mM MgCl_2_. The PCR cycling conditions based on Brandt et al. (2021) were as follows: 98 °C for 30 s, followed by a specific number of cycles of 98 °C for 10 s, annealing for 45 s, and 72 °C for 60 s, with a final elongation at 72 °C for 10 min. For 18S specifically, an annealing temperature of 50 °C and 40 cycles were applied, and the corresponding conditions for COI were 48 °C and 40 cycles. All PCR products were measured via gel electrophoreses and a Qubit 3.0 fluorometer (Invitrogen, Denmark) for quality and quantity. Sequencing and library preparation were performed by the university platform GenSeq (Montpellier University, France). Library prep was performed through a second PCR step to add the Nextera XT Index Kit (Illumina, Hayward, CA, USA) separately for each gene region, including PhiX for standardisation. Sequencing was performed on an Illumina MiSeq instrument with the corresponding reagent kit to obtain 300 bp paired-end sequences. Controls were included at each processing step, with extraction control, PCR negative controls (nanopure water) several positive controls with mouse (*Mus musculus*) DNA to improve decontamination (see bioinformatic methods), and index sequencing control.

**Table 1:** All used Primers, including Illumina sequencing adapters and developed Tuna blocking primer of this study, produced by Eurofins (Ebersberg, Germany).

Name	Direction	Region	Amplicon size bp	Primer Sequence 5′–3′	Publication
mlCOIintF	Forward	Mini-COI	313	GGWACWGGWTGAACWGTWTAYCCYCC	Leray et al. (2013b)
jgHCO2198	Reverse	Mini-COI		TANACYTCNGGRTGNCCRAARAAYCA	Geller, Meyer & Parker (2013)
SSUF04	Forward	18S V1-V2	356	GCTTGTCTCAAAGATTAAGCC	Blaxter et al. (1998)
SSURmod	Reverse	18S V1-V2		CCTGCTGCCTTCCTTRGA	Sinniger et al. (2016)
Adapter	Forward	–	–	TCGTCGGCAGCGTCAGATGTGTATAAGAGACAGMK	ownership Illumina^®^
Adapter	Reverse	–	–	GTCTCGTGGGCTCGGAGATGTGTATAAGAGACAGMK	ownership Illumina^®^
ThunB1	Forward	Mini-COI	Blocking	AACCGGTTGAACAGTCTACCCTCCCCTTGCCGGC-SpC3I	this study

### Blocking primer

In gut content or faecal analyses of generalist predators, when using universal barcode primers, the amount of predator DNA present and its good status (nondegraded compared to stomach contents) can cause the host DNA to monopolize part of the sequencing capacity, lowering the effective sequencing depth and hindering the assessment of prey diversity. However, the use of blocking primers specific to the host including a three-carbon spacer (C3) modification at the 3 ′ end to specifically amplify host DNA shown good results in avoiding this potential pitfall and optimizing the sequencing of prey (Vestheim & Jarman, 2008; Leray et al., 2013a). Therefore, we developed and tested a blocking primer for the COI barcode region (forward primer) of tuna. The ThunB1 primer (see Table 1) was designed on the basis of alignments between *Thunnus thynnus* and some closely related *Thunnus* sp. barcodes from GenBank (Sayers et al., 2019). An *in silico* test was performed allowing a maximum of three mismatches against the Midori-UNIQUE Database (Machida et al., 2017) to optimize the specificity of this primer while avoiding blocking the amplification of potential prey sequences. Ten samples were included twice in the trial: once with and the second time without the defined blocking primer (which was then included at a concentration of ten times that of the amplification primers) to empirically test its efficiency and accuracy.

### Bioinformatics

Data were analysed following the bioinformatic pipeline described by Brandt et al. (2021). The FASTQ files were first processed using Cudadapt (Martin, 2011) to remove all primers and leftover adapters. The stringent error correction algorithm implemented in the program DADA2 (Callahan et al., 2016) was then applied, after pre-filtering reads using a maximum expected error (MaxEE) of 5 and truncation length of 250 bp (with minimum quality; truncQ =2). The parameters used for fragment size selection were an expected total length of 250-350 base pairs (bp) for COI and 300-500 bp for 18S-V1V2 assembled fragments, and a chimaera removal step was included. The output was a list of unique sequences referred to as amplicon sequence variants (ASVs), along with the number of times (reads) they were encountered. To avoid confounding intraspecific diversity and species diversity, particularly for metazoans, processed ASVs were clustered into operational taxonomic units (OTUs) using the program swarm2 (Mahé et al., 2015) with an iterative local threshold d (the maximum number of differences between two ASVs) of 6 for COI and 4 for 18S-V1V2.

Taxonomic assignment was performed at the ASV level using reference databases: Silva release 132 (Quast et al. 2013) for 18S-V1V2 ribosomal sequences and Midori (Machida et al., 2017) for COI. Assignments were performed using the RDP naive Bayesian classifier method (Wang et al., 2007). Possible cross-contaminants introduced during extraction, PCR, and sequencing were removed using the *decontam* R package (using the prevalence method with a threshold of 0.5; (Davis et al., 2018)), with information on the identity and number of reads found in the extract and PCR-negative controls. For all gene regions, the final OTU counts were adjusted using an R-based script (Wangensteen et al., 2018) to account for potential tag switches that are to occur during library preparation (Schnell, Bohmann & Gilbert, 2015). To remove the remaining spurious sequences and particularly possible nuclear-degenerated copies (*numts*), known to occur when amplifying COI with universal primers (e.g., (Song et al., 2008)), the program LULU (Frøslev et al., 2017) was applied to the COI data, with an identity of 0.84 and a cooccurrence of 0.9.

### Analyses and statistics

The definitive datasets for each marker contained OTUs that were taxonomically identified with an RDP bootstrap value exceeding 0.7 at the phylum level, excluding all human and tuna (*Thunnus* sp*.*) matches. COI was shown not to be diagnostic for all distinct tuna species (Viñas & Tudela, 2009); therefore, any sequence assigned to *Thunnus* sp. was considered a host/predator. Furthermore, an additional, better-curated species list was constructed based on the COI results using OTU bootstrap assignment scores of 0.99 RDP or higher. For the 18S dataset, composed of sequences from diverse phyla, including protists and fungi, only metazoan sequences were retained for downstream analysis.

A dual treatment was then applied to the OTU tables. First, the diet was summarized in simplified tables according to the presence-absence of OTUs, including the assessment of the percentage of occurrence (POO) and the weighted percentage of occurrence (wPOO) of prey taxa, both with a minimum 1% occurrence threshold. Second, to test the accuracy of the semi-quantitative information that the number of reads could deliver, RRA (Deagle et al., 2019; Mychek-Londer, Chaganti & Heath, 2020) was estimated for each prey item.

The data for all gene regions were analysed in R using the vegan package (R version 3.4.4; Oksanen et al., 2019) and rmarkdown (Xie, Allaire & Grolemund, 2018) to estimate the Sørensen index (Sørensen, 1948; based on presence-absence) and Euclidean distance (based on read abundance equalized per sample) to quantify the compositional dissimilarity among samples. The difference between the results obtained on the basis of COI and 18S-V1 was tested using the Mantel test. We applied analysis of similarities (ANOSIM) as a nonparametric statistical test for identifying parameters likely to be significantly related to differences in the diet composition (size, sex, and weight of the fish, year of sampling). The homogeneity of the sample dispersion was described using the betadisper function (distances to centroid) and tested for significance using analysis of variance (ANOVA).

We then built a generalized linear model (GLM) with the family set to ‘binomial’ to predict the presence/absence of each prey taxon (dependent variable) depending on the independent variable “year of catch” (factorial) as well as the linked (because both were correlated with size) factors “predator body mass” (in kg, log10 transformed) and “mouth length” (in cm, log10 transformed). This ‘full’ model as well as the ‘null’ model was tested against forward and backward selection, and the model was chosen according to the best AIC (Burnham & Anderson, 2004) and tested by Chi-squared test, as detailed in Günther et al. (2014).

### Data storage

All raw sequences have been stored in the Sequence Read Archive (SRA) under accession numbers SRR134477398–SRR13447844, with BioSamples SAMN17349101–SAMN17349148, ID PRJNA692564, respectively.

## Results

### General findings

Metazoans were detected in 45 and 44 of the 48 ABFT stomachs analysed on the basis of COI and 18S sequences, respectively. Two of the three remaining ABFT stomachs were nearly empty, as noted during dissection-homogenization, while we have no obvious explanation for the failure of detection in the third stomach. The number of generated reads was 40 to 100,952 (Ø40,000; after cutting primers) for 18S, with 142 to 27,237 identifications after stringent cleaning and 1329 to 53,998 taxonomic identifications (Ø30,000), leading to the identification of 832 to 41,528 sequences (after removing predator DNA, all data in Table S2/Appendix 2).

The use of the general eukaryotic 18S primer pair revealed protist barcodes (which were excluded from the rest of the analyses), including barcodes for several algae and other nonmetazoans (seen as nonintentional prey) from the following taxa Apicomplexa, Dinoflagellata, Fungi, Ochrophyta, Phragmoplastophyta, Protalveolata, and Viridiplantae. Thus, the original metazoan RRA was 58.2%, with nonmetazoans accounting for slightly less than half the RRA but excluded from further analyses as they were not the target of this study.

**Figure 1:**
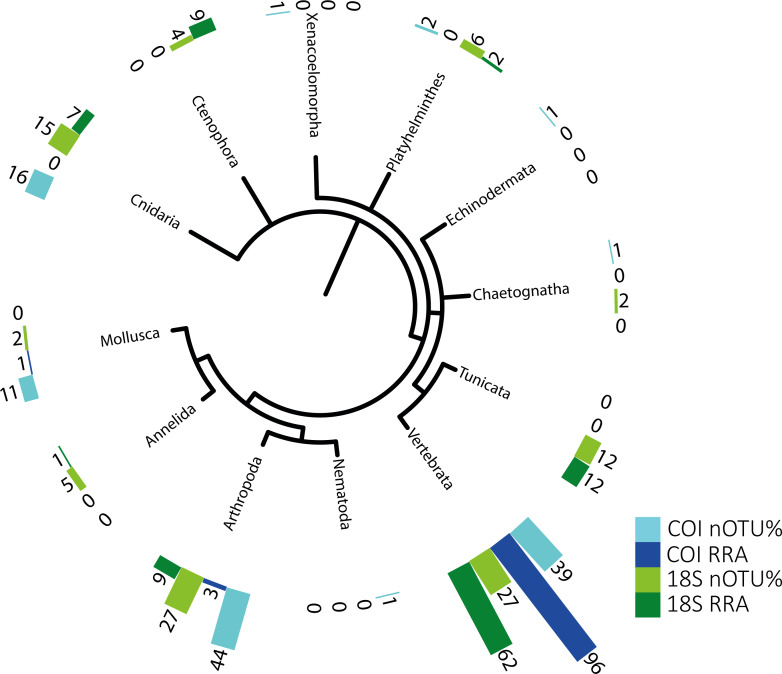
Phylogenetic tree of detected taxa. Summary of the metazoan taxa detected in the stomachs of 48 specimens collected over 4 years. Light blue indicate the taxa identified with COI, and dark blue, those identified with RRA. Light green and dark green indicate the same for the 18S identifications.

The potential metazoan prey detected using the 18S and COI sequences are summarized in Table 2 and were used for the semi-quantitative analysis presented in Fig. 1. A second, well-curated list based on COI sequences, with taxonomic assignment reaching the species level (RDP bootstrap ≥ 0.99) to facilitate comparison with morphological data, is provided in Table S3/Appendix 3. The genetic approach revealed a broad range of metazoans in the stomachs of the tunas. It is important to keep in mind that not all of the identified taxa reflect active predation (some may represent secondary predation, i.e., prey eaten by the tuna’s prey), although this possibility cannot be discarded for many taxa.

**Table 2:** Numbers and values of detected Phylas. Table 2 Summarized values from all samples (18S: upper table, COI: lower table) regarding biodiversity (count of OTUs and their number of detections), presence/absence-based approaches with the percentage of occurrence (POO) and weighted percentage of occurrence (wPOO), and semi-quantitative information, including the relative read abundance (RRA).

Taxa	Count of OTU	Sum of OTU		POO	WPOO	RRA
Annelida	4	5	0.019		0.015	0.009
Arthropoda	17	22	0.142		0.128	0.104
Cnidaria	15	40	0.189		0.171	0.161
Ctenophora	9	35	0.151		0.155	0.178
Platyhelminthes	6	6	0.047		0.036	0.029
Tunicata	10	34	0.170		0.161	0.152
Vertebrata	13	51	0.283		0.334	0.367

Taxa	Count of OTU	Sum of OTU	POO	WPOO	RRA
*Engraulis encrasicolus*	2	62	0.301	0.339	0.488
*Eledone cirrhosa*	1	1	0.008	0.006	0.013
*Cepola macrophthalma*	1	2	0.016	0.044	0.044
*Scomber colias*	1	14	0.114	0.106	0.058
*Sardina pilchardus*	1	24	0.195	0.193	0.265
*Sprattus sprattus*	1	20	0.163	0.145	0.088
Arthropoda	32	58	0.098	0.086	0.020
Chaetognatha	2	4	0.000	0.000	0.000
Vertebrata (others)	22	35	0.073	0.059	0.018
Cnidaria	14	24	0.016	0.010	0.001
Echinodermata	1	2	0.000	0.000	0.000
Mollusca	9	17	0.016	0.012	0.002
Xenacoelomorpha	2	2	0.000	0.000	0.000

The identified metazoan group with the highest diversity of species/taxa (see Fig. 1) was arthropods (41 COI, 27 18S), all but one of which were identified as crustaceans (and the remaining as an insect). However, crustaceans were detected for both gene regions in only 24 stomachs, and at a lower abundance (COI, RRA = 2.6%;18S, RRA = 8.5%). Most of the crustaceans identified according to COI sequences belonged to plankton groups of Maxillopoda (18 OTUs; RRA = 0.7%), Branchiopoda (2 OTU; 1.3%), Ostracoda (2 OTUs, RRA < 0.01%), and Malacostraca (20 OTUs, RRA = 0.6%), including shrimp and crab species. Similar results, although quantitatively slightly different, were obtained for arthropods according to the 18S data, including the identification of Maxillopoda (18 OTUs, RRA = 4.7%), Branchiopoda (1 OTU, RRA = 0.1%), Malacostraca (7 OTUs, RRA = 3.5%) and one insect (Hemiptera, RRA > 0.01).

**Figure 2:**
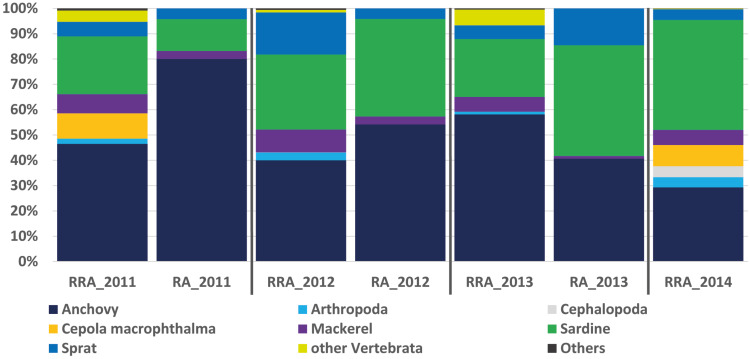
Relative abundance of stomach content. Relative read abundance of stomach contents: left, based on this study using the relative read abundance (RRA) per year, and right, the relative abundance (RA) based on morphological data from Van Beveren et al. (2017).

The second most diverse taxonomic group (but detected the most frequently) was vertebrates (see also Fig. 2), which mostly included fish according to both gene regions, with nearly all tuna containing fish DNA in their stomachs. Within predator individuals identified based on the COI region included 40 *Engraulis encrasicolus* (RRA = 43.4%), 38 *Sardina pilchardus* (RRA = 30%), 32 *Sprattus sprattus* (RRA = 8%) and 21 *Scomber colias* (RRA = 7%). Vertebrates were also the most abundant taxonomic group detected with 18SV1 (RRA = 61.6), but most sequence were assigned to *Alosa alosa* (allis shad), which is unlikely based on the limitations of species identification combined with the relatively high abundance of sequences of this particular species in reference databases.

The genetic approach also allowed the detection of soft-bodied potential prey items, with 15 different cnidarian taxa detected from the COI and 18S data (1.8% and 6.7%, respectively; see Table 2). Overall, one-third (RRA = 27.1%) of the taxa detected from 18S sequences were soft-bodied metazoans (ctenophores, cnidarians, and tunicates), including four different OTUs of Ctenophores (all Tentaculata, RRA = 8.7%) and 12 different OTUs of tunicates (salps and Appendicularia, RRA = 11.7%). Molluscs were detected mostly using COI (RRA = 1.4%), including 5 cephalopods detected in 7 samples with Octopoda, Sepiolida, and Teuthida (RRA = 1.3%), 5 gastropods (RRA = 0.7%) from the genus *Creseis,* and one Bivalvia (RAA > 0.01%).

### Comparison with morphological data

The RRA (COI) calculated per year showed the same trends as the relative abundance (RA) based on the morphological data from Van Beveren et al. (2017, see Fig. 2). A *t*-test confirmed that no significant difference could be found between RRA and RA, either per year (2011, 2012, 2013) or overall. For example, both morphological and COI analyses indicated that fish were the dominant prey, with four common and clearly identified fish species being recorded (see Fig. 2). Among the three years sampled for comparison, both the morphological and molecular COI datasets ranked anchovy (39–76% and 40–58% for RA and RRA per year, respectively), sardine (12–42% and 23–30% for RA and RRA per year, respectively) and sprat (4–14% and 5–17% for RA and RRA per year, respectively) as the most consumed prey. Less abundant common species, such as *Cepola macrophthalma, Gadiculus argenteus, Lesueurigobius friesii, Merluccius merluccius, Scomber scombrus,* and *Trisopterus sp.*, were also present in both inventories. However, five additional rare species were identified only morphologically, and 18 were identified only with COI (see Appendix 3).

### Statistical analysis

**Figure 3:**
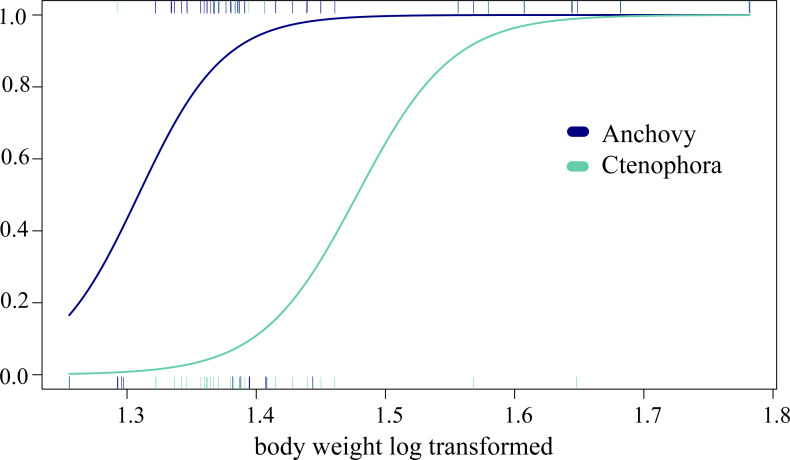
Changing predation with increased predator body weight. The significant GLM results for the relationship between increased predation with increased weight (log transformed, *x*-axis) and the predicted presence/absence (*y*-axis) of prey for Ctenophora (18S-based) and anchovy (COI) predation.

In the analysis of compositional dissimilarity, none of the available fish descriptors (weight, sex, size, etc.) showed any significant relationship with the stomach contents (based on presence-absence and read abundance, see the R markdown Appendix 4). However, weight and mouth length showed significantly heterogeneous dispersions (*p* = 0.0016 for COI testing weight at euclidian distribution, all others with *p* < 0.001), which is likely to affect the correlation analysis. The two factors were, however, correlated, as they are both proxies of ABFT size. General linear models (GLMs) showed that ABFT weight/size and year of catch were significantly related to the presence/absence of anchovy, arthropods, and Ctenophora values (coefficient estimate and std. error) given in Table 3, based on the full models, and AIC for all the models in Table S5/Appendix 5. The detected increases in prey types; anchovy and Ctenophora are shown according to size in Fig. 3.

**Table 3:** GLMs output. Output from the GLMs, with *p*-values, coefficient estimate and std. error, based on presence/absence data for both gene regions. The full model was always used based on the AIC (see Table S5/Appendix 5).

	**Ctenophora (18S)**				**Arthropoda(18S)**				**Anchovy (COI)**			
	**Estimate**	**Std. Error**	**value**	** **	**Estimate**	**Std. Error**	**value**	** **	**Estimate**	**Std. Error**	**value**	
(Intercept)	11.70	13.49	0.39		−24.25	13.26	0.07	.	−1.24	16.37	0.94	
Year2012	2.49	2.81	0.38		−3.65	2.40	0.13		4.45	3.20	0.16	
Year2013	4.55	2.83	0.11		−4.29	2.37	0.07	.	5.06	2.99	0.09	.
Year2014	4.86	2.92	0.10	.	−4.37	2.57	0.09	.	2.73	2.92	0.35	
log body mass	26.95	12.50	0.03	*	−16.93	9.38	0.07	.	30.18	14.36	0.04	*
log Length	−50.39	28.89	0.08	.	47.39	24.63	0.05	.	−39.84	29.63	0.18	

### Blocking primer results

**Figure 4:**
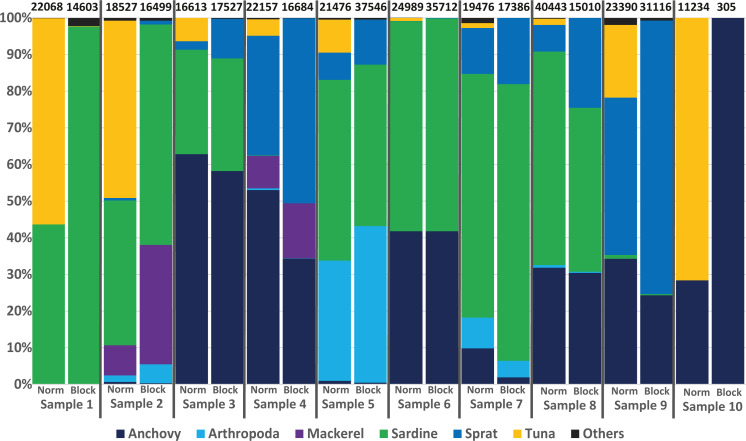
Avoiding predator DNA using a blocking primer. One-to-one comparison of relative sequences detected between 10 samples with and without the developed blocking primer. The numbers at the top present the total number of sequences per sample.

To avoid the possible overdetection of predator DNA (ABFT sequences in this case), a blocking primer for the COI gene region of *Thunnus thynnus* (and other *Thunnus* spec.) was developed and tested within this study. Nearly all samples analysed produced a high number of *Thunnus* sequences. However, in the 10 samples analysed with the blocking primer, no *Thunnus* DNA was found, showing the high efficiency of the primer (see Fig. 4).

Regarding the specificity of the primer, *in silico* analyses suggested a low amount of (if any) undesired blocking (i.e., for non-tuna species), as only 92 species (including all tuna species) among the tested 110,726 species in the Midori database could be blocked when accepting a blocking effect with up to three mismatches. The exact list, including the number of mismatches, is provided in Table S6/Appendix 6. Primers designed to block all *Thunnus* species might potentially prevent the amplification of sequences from 8 other Scombridae if some mismatches were accepted. However, the detection of *Scomber colias* was positive with or without blocking primer in the present study, suggesting that the blocking primer did not prevent amplification in this family. Moreover, compared to data from the same samples obtained without the blocking primer (Fig. 4), the presence-absence of other species did not differ significantly: only one OTU represented by fewer than 100 reads (less than 0.5% of the reads) was found in one sample but not in the other. Both *in silico* and empirical tests thus also suggest a good specificity of the applied primer.

## Discussion

Following the recommendation of Deagle et al. (2019) to conduct species-by-species comparative studies when working with stomach contents (rather than faeces), our comparative analysis showed reasonable congruence between the morphological inventories and RRA based on the COI metabarcoding of stomach contents, at least for vertebrate prey. The study also has comparable findings for fish preying on invertebrates. The main ABFT prey in the Mediterranean have been described as fish, cephalopods and crustaceans (Karakulak, Salman & Oray, 2009; Van Beveren et al., 2017). Using individuals from the same collection employed in the study of Van Beveren et al. (2017), the most important prey species in terms of both occurrence and relative abundance were found to be similar in the morphology-based and molecular-based inventories; these species were anchovy (*Engraulis encrasicolus*), sardine (*Sardina pilchardus*), sprat (*Sprattus sprattus*) and mackerel (*Scomber colias*). Moreover, the results obtained from the 18S metabarcoding of stomach contents highlighted a high diversity of gelatinous taxa in the ABFT diet, which was stable across years; the importance of these taxa seemed to increase with ABFT weight and, thus, likely with age.

### The diet of ABFT

Microscopy-based studies have shown that ABFT exhibits little prey size selectivity (Fromentin & Powers, 2005; Karakulak, Salman & Oray, 2009), and stable isotope analyses have indicated that ABFT, similar to other tuna species, is an opportunist and generalist predator (Sarà & Sarà, 2007; Varela et al., 2014). The Pacific bluefin tuna (*Thunnus orientalis*) consumes the same types of prey, with a diet dominated by fish, crustaceans, and cephalopods, including an age-based shift from crustaceans to more fish-based food during growth (Shimose et al., 2013; Hiraoka et al., 2019). These studies suggest the generalist behaviour of tuna, as well as influences of age and geographical zone. To date, the use of metagenomics approaches in diet studies has been limited and mostly restricted to larvae (Kodama et al., 2017; Kodama et al., 2020). Here, the applied molecular approach detected an even higher biodiversity of potential prey than the morphological inventory, providing additional support to the generalist behaviour hypothesis for tuna. Additional potential prey include fish species not detected in morphological assessments, tunicates, cnidarians, ctenophores and molluscs, and possibly even echinoderms. The anecdotal detection of chicken DNA (*Gallus gallus;* detected by COI 100% identity verified on Genbank) in only one of the ABFT stomachs, with 5% of reads, and only three other detected taxa (sprat, sardine, and shrimp; thus allowing us to favour real biological evidence over the hypothesis of possible contamination) most likely originated from left-over food from humans and could indicate either scavenging or even less target-oriented foraging then hitherto considered. Such opportunistic swallowing of potential food would, however, be in line with the extremely large amounts of different sized plastic debris found in ABFT stomachs (Romeo et al., 2012).

One of the major pitfalls of the use metabarcoding for diet reconstruction is the inability to delineate the set of prey from the list of species resulting from secondary predation (King et al., 2008; Deagle et al. (2019)), or swallowed planktonic species in marine systems (Hardy et al., 2017). This is particularly problematic when manipulating presence-absence data to make a simple list of items that avoids the potential bias of a quantitative approach based on the number of reads. For distinguishing between target and non-target prey, the approach based on RRA (Alberdi et al., 2018; Deagle et al., 2019; Mychek-Londer, Chaganti & Heath, 2020) provides important information. Due to the differential digestion rates of prey, Deagle et al. (2019) suggested that faecal analyses could be more reliable than those of stomach contents. However, these authors also noted that biases due to the differential kinetics of prey digestion may be predator specific and encouraged pilot studies on stomach contents. Our results tend to show an absence of such pitfalls in the present case study (ABFT stomach contents), as the detection and relative abundance of prey resulting from the morphological approach or RRA (i.e., fish and cephalopods) were very similar. This encouraging result was surprising for two other reasons. First, it suggests that well-known PCR biases (Acinas et al., 2005; Kanagawa, 2003) still allow reliable semi-quantitative analysis in some contexts, such as the tuna diet. Second, the choice of the High-Fidelity Phusion Taq Polymerase used in this study was driven by the quality of the sequences obtained, whereas among a panel of 6 high-fidelity enzymes tested on synthetic oligos with various GC contents, this polymerase performed less reliably in terms of quantitative data (Nichols et al., 2018). The good relationship obtained here between the RRA and RA data is thus extremely encouraging in a framework focused on quantitative data where a more appropriate polymerase could be chosen to reduce abundance biases (Nichols et al., 2018) or q–PCR tests could be included to estimate the absolute DNA concentration of specific prey taxa (Zemb et al., 2020).

Some invertebrates, such as annelids, platyhelminthes, nemerteans, and xenacoelomorphs, are unlikely to all be intentional prey. In fact, these groups exhibit varied detection patterns (single taxa and unique detections) associated with generally low values (RRA ≤ 3%). In contrast, groups such as crustaceans showed considerable diversity associated with more frequent detection and a sometimes higher RRA (2.6% for COI and up to 8.5% for 18S). Their frequent presence, associated with a relatively high abundance even compared to fishes, is not surprising and has often been reported for ABFT juveniles, either through morphological or isotope analysis (Sinopoli et al., 2004; Varela et al., 2014). Based on stable isotope analyses, northern krill (*Meganyctiphanes norvegica*) and other shrimp have been shown to be important food sources for ABFT, albeit more frequently reported in juveniles than adults (Fromentin & Powers, 2005; Sarà & Sarà, 2007). Many of the stomachs included almost completely intact (i.e., not digested and hardly chewed) fish prey, and some of the identified zooplankton taxa are known to be components of the diets of fish such as anchovies or sardines in the Gulf of Lion (Le Bourg et al., 2015; Plounevez & Champalbert, 2000)); these two small pelagic fish become key prey items for bluefin tuna with increasing age (see also past studies, e.g., Van Beveren et al., 2017). Indeed, the GLM indicated increasing consumption of arthropods as well as anchovy prey with fish size (see Fig. 3, Table 3). The remains of some crabs and shrimp found in the morphological analyses could not be specifically identified, while the molecular approach identified species such as *Goneplax rhomboides*, *Liocarcinus vernalis,* and *Jaxea nocturna.* Whether directly hunted or not, crustaceans appear to be a nonnegligible source of food for ABFT*.* Finally, the RRA for Malacostraca and Cephalopoda was low, at <1.4%, which is in line with the results of visual detection (Van Beveren et al., 2017) and may confirm rare prey items in this case, not necessarily indicating secondary predation. This confirms that the interpretation of the status of taxa (prey, secondary prey, incidental ingestion) associated with a low RRA should be interpreted with caution or based on robust previous knowledge of the predator diet. Interestingly, however, especially for these last groups, the identification of prey leftovers is often limited due to poor conservation, whereas genetic analyses allow more precise taxonomic identification.

### The importance of gelatinous taxa in the ABFT diet

Here, we complemented the COI metabarcode data with 18S data (although the latter are not ideal for the most important vertebrate prey) to specifically target invertebrates (Cowart et al., 2015; Cowart et al., 2020). Like COI, vertebrates showed the highest RRA for 18S (>60%), directly followed by gelatinous prey. In case of ctenophores, the rate of detection tended to increase with ABFT size, indicating increasing consumption. As validated here by comparing COI RRA with morphological RA for vertebrates, previous studies have shown a good relationship between the 18S read abundance and morphological inventories of ctenophores and cnidarians (Günther et al., 2018). The large numbers of cnidarians, ctenophores, and even salps that were detected (RRA 27% of metazoan 18S detection altogether) tend to confirm that gelatinous taxa (including both benthic and planktonic taxa) are usually found in the ABFT diet, as this has also been shown for other top marine predators (Cardona et al., 2012; Hays, Doyle & Houghton, 2018). Although gelatinous preys could also be a crucial part of the diet of some fish (see e.g., Ayala et al., 2018 about for the European eel), the nutritional content of gelatinous plankton is poor, so that their importance in the diet of bluefin tuna remains debatable. However, the consumption of gelatinous taxa by bluefin tuna, and in general by top predators, may play an important role for ecosystem functioning. The predation of gelatinous prey has been poorly explored in trophic or ecosystemic models, but its impact on the trophodynamics of large pelagic fish could thus be nonnegligible. The warming of the Mediterranean Sea due to climate change is expected to affect the species composition and occurrence of hydroid communities (González-Duarte et al., 2015). Furthermore, the overexploitation of some fish stocks together with environmental changes is suspected to favour cascading effects, inducing an increasing abundance of jellyfish or gelatinous zooplankton, as shown by the spectacular invasion of *Mnemiopsis leidyi* observed in the Black Sea (Daskalov et al., 2007; Galil, 2012; Ghabooli et al., 2013). In this context of global change in the Mediterranean Sea, our results appear to be a positive outcome, as ABFT, a major top predator in the Mediterranean, may mitigate outbreaks of gelatinous species through top-down regulation. It would be of great interest to further investigate this potential top-down effect through proper trophic/ecosystemic modelling simulations.

Our results also indicate that the use of stomach contents may be preferrable to faeces for ABFT because the morphology of the tuna stomach allows a very clear-cut separation of the stomach contents from the stomach wall, avoiding any significant contamination by the host DNA, which would be expected with faeces. The blocking primers developed in this study may, however, be useful for faeces analysis, as they showed effective blocking of tuna DNA during PCR, and the differences were not significant compared to data from the same stomachs obtained without blocking primers. Additionally, the use of blocking primers can hinder the detection of potential cannibalism, which has been observed towards ABFT larvae (Uriarte et al., 2019), and also early juveniles (Fromentin, 2020, pers. comm) in large ABFT adults.

## Conclusion

The results presented here validate the use of metabarcoding to assess the diet of a top predator, Atlantic bluefin tuna (ABFT). In addition to providing a comprehensive list of prey taxa, our results unexpectedly showed a good match between semi-quantitative estimates (relative abundance) inferred from morphological and molecular (COI) inventories of stomach contents. They further confirmed the more opportunistic feeding behaviour of ABFT than hitherto indicated. Perhaps more importantly, the concomitant use of the 18S ribosomal barcode finally confirmed the importance of diverse gelatinous prey in the ABFT diet. The work presented here thus encourages the further use and improvement of molecular approaches to better understand trophic interactions and their predicted evolution in a changing environment.

##  Supplemental Information

10.7717/peerj.11757/supp-1 Supplemental Information 1 Samples, Number of reads, Found species, GLM models, and in silico block

10.7717/peerj.11757/supp-2 Supplemental Information 2 Rmarkdown including all statistical scripts and output of this study

## Additional Information and Declarations

### DNA Deposition

The following information was supplied regarding the deposition of DNA sequences: The raw sequences are available at the Sequence Read Archive (SRA): SRR13447739–SRR13447844, with BioSamples SAMN17349101–SAMN17349148, BioProject: PRJNA692564.

### Data Availability

The following information was supplied regarding data availability: The data is available in the Supplemental Files.
