# Supplementary material for: Metabarcoding confirms the opportunistic foraging behaviour of Atlantic bluefin tuna and reveals the importance of gelatinous prey
Source: PeerJ. 2021 Aug 13;9:e11757. doi: 10.7717/peerj.11757 (PMC8366523; doi:10.7717/peerj.11757)
Supplement: Supplemental Information 2 [file peerj-09-11757-s002.pdf]

Metabarcoding the tuna stomach

Babett Günther, including scripts from Deagle et aL. 2019, and Günther et al. 2015

18/05/2021

R Markdown

This is an R Markdown document. Markdown is a simple formatting syntax for authoring HTML, PDF, and MS Word documents. For more details on using R Markdown see http://rmarkdown.rstudio.com.

When you click the **Knit** button a document will be generated that includes both content as well as the output of any embedded R code chunks within the document. You can embed an R code chunk like this:

######EXPLANATION GUIDE #### PROCESSING IN 5 STEPS ##### 1. Read and process datasets ##### 2. calculating values RRA,POO,wPOO,FOO for 18S, ##### 3. calculating values RRA,POO,wPOO,FOO for COI ##### 4. RRA PER YEAR TO COMPARE TO MOPHOLOGICAL RA, relative abundance data ##### 5.Statistical distribution tests with euclidean distance/Sorensen dissimilarity, based on OTU level, finding driving factors ##### 6.GlMs and predictive models and their Drawings

```
##### 1. Read and process datasets
#reading in all data
data_1 <-read.csv ("18S_Metazoa.csv",  sep=";", dec=",", header=TRUE, check.names=TRUE)
str(data_1)
```

1. Read and process datasets

```
## 'data.frame':    74 obs. of  51 variables:
##  $ ClusterID: Factor w/ 74 levels "OTU_10","OTU_106",..: 36 10 11 15 23 17 33 18 42 32 ...
##  $ Phylum   : Factor w/ 7 levels "Annelida","Arthropoda",..: 1 1 1 1 2 2 2 2 2 2 ...
##  $ Species  : Factor w/ 47 levels "Acartia clausii",..: 5 4 4 4 1 3 8 10 11 17 ...
##  $ M275     : int  0 0 0 0 0 0 0 0 0 0 ...
##  $ J290     : int  0 0 0 0 0 0 0 0 0 0 ...
##  $ J269     : int  0 0 0 0 0 0 0 0 0 0 ...
##  $ M281     : int  0 0 0 0 0 0 0 0 0 0 ...
##  $ M261     : int  0 0 0 0 0 0 0 0 0 2 ...
##  $ M198     : int  0 0 7 0 679 0 0 0 0 0 ...
##  $ M273     : int  0 0 0 0 0 0 0 8 0 0 ...
##  $ J233     : int  0 0 0 0 0 0 0 0 0 0 ...
##  $ J201     : int  0 0 0 0 0 0 0 0 0 0 ...
##  $ M280     : int  0 0 0 10 0 0 0 0 0 0 ...
##  $ J222     : int  0 0 0 0 0 0 0 0 0 0 ...
##  $ M118     : int  0 0 0 0 0 0 0 0 0 0 ...
##  $ M277     : int  0 0 0 0 0 0 0 0 0 0 ...
```

```
##  $ J221     : int  0 0 0 0 0 0 0 0 0 0 ...
##  $ M262     : int  0 0 0 0 0 0 0 0 0 0 ...
##  $ M279     : int  0 0 0 0 0 0 2 0 0 0 ...
##  $ J226     : int  0 0 0 0 0 0 0 0 0 0 ...
##  $ J53      : int  0 0 0 0 0 0 0 0 0 0 ...
##  $ J215     : int  0 0 0 0 0 0 0 0 0 0 ...
##  $ M117     : int  0 0 0 0 0 0 0 0 229 0 ...
##  $ M33      : int  0 0 0 0 0 0 0 0 0 0 ...
##  $ J47      : int  0 0 0 0 0 0 0 0 0 0 ...
##  $ J276     : int  0 0 0 0 0 0 0 0 0 0 ...
##  $ M59      : int  0 0 0 0 0 0 0 0 0 0 ...
##  $ J52      : int  0 0 0 0 0 0 0 0 0 0 ...
##  $ M29      : int  0 0 4 0 0 0 0 0 0 0 ...
##  $ J138     : int  0 0 0 0 0 0 0 0 0 0 ...
##  $ J125     : int  0 0 0 0 0 0 0 0 0 0 ...
##  $ J121     : int  0 0 0 0 50 0 0 0 0 0 ...
##  $ M49      : int  0 0 0 0 0 0 0 0 0 0 ...
##  $ J144     : int  0 0 0 0 0 0 0 0 0 0 ...
##  $ J134     : int  0 0 0 0 0 0 0 0 0 0 ...
##  $ J150     : int  0 0 0 0 0 0 0 0 0 0 ...
##  $ J227     : int  0 0 0 0 0 0 0 0 0 0 ...
##  $ J93      : int  1 0 0 0 0 0 0 0 0 0 ...
##  $ J57      : int  0 0 0 0 0 0 0 0 0 0 ...
##  $ M30      : int  0 0 0 0 0 0 0 0 0 0 ...
##  $ J46      : int  0 0 0 0 0 0 0 3 0 0 ...
##  $ J145     : int  0 0 0 0 0 9 0 0 0 0 ...
##  $ J96      : int  0 0 0 0 0 0 0 0 0 0 ...
##  $ J164     : int  0 0 0 0 0 0 0 0 0 0 ...
##  $ M123     : int  0 0 0 0 0 0 0 0 0 0 ...
##  $ J128     : int  0 0 0 0 0 0 0 0 0 0 ...
##  $ M253     : int  0 0 0 0 0 0 0 0 0 0 ...
##  $ J229     : int  0 0 0 0 0 0 0 0 0 0 ...
##  $ J217     : int  0 14 0 0 0 0 0 0 0 0 ...
##  $ J205     : int  0 0 0 0 0 0 0 0 0 0 ...
##  $ J170     : int  0 0 0 0 0 0 0 0 0 0 ...
```

```
d1_1<-data_1
data_2 <-read.csv ("COI.csv",  sep=";", dec=",", header=TRUE, check.names=TRUE)
str(data_2)
```

```
## 'data.frame':    91 obs. of  51 variables:
##  $ ClusterID: Factor w/ 91 levels "OTU_1","OTU_101",..: 22 30 35 34 23 24 27 21 19 18 ...
##  $ Taxon    : Factor w/ 9 levels "Arthropoda","Chaetognatha",..: 1 1 1 1 1 1 1 1 1 1 ...
##  $ Species  : Factor w/ 84 levels "Acartia_clausii",..: 4 33 46 49 52 58 79 11 26 82 ...
##  $ Best     : Factor w/ 15 levels "anchovy","Arthropoda",..: 2 2 2 2 2 2 2 2 2 2 ...
##  $ M275     : int  0 0 0 0 0 0 0 0 0 0 ...
##  $ J290     : int  0 0 0 0 0 0 0 0 0 0 ...
##  $ J269     : int  0 0 0 0 0 0 0 0 0 0 ...
##  $ M281     : int  0 0 0 0 0 0 0 0 0 0 ...
##  $ M261     : int  0 0 0 0 0 0 0 0 0 0 ...
##  $ M198     : int  0 0 0 0 0 0 0 0 0 0 ...
##  $ M273     : int  0 0 0 0 0 0 0 0 0 0 ...
##  $ J233     : int  0 0 0 0 0 0 0 0 0 0 ...
##  $ J201     : int  0 0 0 0 0 0 0 0 0 0 ...
```

```
##  $ M280     : int  0 0 0 0 3 0 0 0 0 0 ...
##  $ J222     : int  0 0 0 0 0 0 0 0 0 0 ...
##  $ M118     : int  0 2 0 0 0 3 0 0 0 0 ...
##  $ M277     : int  0 0 0 0 0 0 0 0 0 0 ...
##  $ J221     : int  0 0 0 0 0 0 0 0 0 0 ...
##  $ M262     : int  0 0 0 0 0 0 0 0 0 0 ...
##  $ M279     : int  0 0 0 0 0 0 0 0 0 0 ...
##  $ J226     : int  0 0 0 0 0 0 0 0 0 0 ...
##  $ J53      : int  0 0 0 0 0 0 0 0 0 0 ...
##  $ J215     : int  0 0 0 0 0 0 0 0 0 0 ...
##  $ M117     : int  0 0 0 0 0 0 0 0 0 0 ...
##  $ M33      : int  0 0 0 0 0 0 0 0 0 0 ...
##  $ J47      : int  0 0 0 0 0 0 0 0 0 0 ...
##  $ J276     : int  0 0 0 0 0 0 0 0 0 0 ...
##  $ M59      : int  0 0 0 0 0 0 0 0 0 0 ...
##  $ J52      : int  0 0 0 0 0 0 0 0 0 0 ...
##  $ M29      : int  0 0 0 0 0 0 0 0 0 0 ...
##  $ J138     : int  0 0 0 0 0 0 0 0 0 0 ...
##  $ J125     : int  0 0 0 0 0 0 0 0 0 0 ...
##  $ J121     : int  0 0 0 0 0 0 0 0 0 0 ...
##  $ M49      : int  0 0 0 0 0 0 0 0 0 0 ...
##  $ J144     : int  0 0 0 0 0 0 0 0 0 0 ...
##  $ J134     : int  0 0 0 0 0 0 0 0 0 0 ...
##  $ J150     : int  0 0 0 0 0 0 3 0 0 0 ...
##  $ J227     : int  0 0 0 0 0 0 0 0 0 0 ...
##  $ J57      : int  0 0 0 0 0 0 0 0 0 0 ...
##  $ M30      : int  0 0 0 0 0 0 0 0 0 0 ...
##  $ J46      : int  0 0 0 0 0 0 0 0 0 0 ...
##  $ J145     : int  0 0 0 0 0 0 0 0 0 0 ...
##  $ J96      : int  0 0 0 0 0 0 0 0 0 0 ...
##  $ J164     : int  0 0 0 0 0 0 0 0 0 0 ...
##  $ M123     : int  0 0 0 0 0 0 0 0 0 0 ...
##  $ J128     : int  0 0 0 0 0 0 0 0 0 0 ...
##  $ M253     : int  0 0 0 0 0 0 0 0 0 0 ...
##  $ J229     : int  0 0 0 0 0 0 0 0 0 0 ...
##  $ J217     : int  0 0 0 0 0 0 0 0 0 0 ...
##  $ J205     : int  0 0 2 2 0 0 0 4 4 4 ...
##  $ J170     : int  0 0 0 0 0 0 0 0 0 0 ...
```

```
#reading metatdata
dm <-read.csv ("metaCOI.csv", sep=";", dec=",", header=TRUE,check.names=TRUE,row.names = 1)
dm2 <-read.csv ("meta18S.csv", sep=";", dec=",", header=TRUE,check.names=TRUE,row.names = 1)
#reading data from Morpho study
morp <-read.csv ("morpho_year_best.csv", sep=";", dec=".", header=TRUE,check.names=TRUE,row.names = 1)
#18S
d1_1$ClusterID <- NULL
d1_1$Species <- NULL
#sum up identic species ##### this process is on Phylum level not on OTU level
d1_2 <- ddply(d1_1,"Phylum",numcolwise(sum))
#first row speices name
df1_3 <- data.frame(d1_2[,-1], row.names= d1_2[,1])
#delete fish without any prey, and undetected prey after processing
i <- (colSums(df1_3) != 0)
df1_4 <- df1_3[, i]
```

```
df1_5 <- subset(df1_4, select = -J93) #( none in COI, to better comparison deleted here)
df1_6<- df1_5 [rowSums(df1_5) != 0, ]
#same for COI
#count are most important species, based on previous tests, rest count together on phyla
d2_1 <- data_2
d2_1$ClusterID <- NULL
d2_1$Species <- NULL
d2_1$Taxon <- NULL
#sum up identic species ##### this process is on species level not on OTU level
d2_2 <- ddply(d2_1,"Best",numcolwise(sum))
#first row speices name
df2_3 <- data.frame(d2_2[,-1], row.names= d2_2[,1])
#delete fish without any prey, and undetected prey after processing
i <- (colSums(df2_3) != 0)
df2_4 <- df2_3[, i]
df2_5<- df2_4[rowSums(df2_4) != 0, ]
write.csv (df2_5,'COI_clean.csv')

##### Now the same, but on OTU level for statistical analyses
d3_1<-data_1 #18S but now working on OTU level
d4_1<-data_2 #COI on OTu level
d3_1$Phylum <- NULL
d3_1$Species <- NULL
#sum up identic species ##### this process is on species level not on OTU level
d3_2 <- ddply(d3_1,"ClusterID",numcolwise(sum))
df3_3 <- data.frame(d3_2[,-1], row.names= d3_2[,1])
i <- (colSums(df3_3) != 0)
df3_4 <- df3_3[, i]
df3_5 <- subset(df3_4, select = -J93) #(ONLY 2 SEQUENCES IN 18S, none in COI, to better comparsion dele
df3_6<- df3_5 [rowSums(df3_5) != 0, ]
df3_7<-t(df3_6)
df3_8pa<-decostand(df3_7, method="total")
df3_8<-decostand(df3_7, method="pa")
df3_8<-data.frame(df3_8)
df3_8pa<-data.frame(df3_8pa)
##forCOI
d4_1$Best <- NULL
d4_1$Species <- NULL
d4_1$Taxon <- NULL
#sum up identic species ##### this process is on species level not on OTU level
d4_2 <- ddply(d4_1,"ClusterID",numcolwise(sum))
#first row speices name
df4_3 <- data.frame(d4_2[,-1], row.names= d4_2[,1])
#delete fish without any prey, and undetected prey after processing
i <- (colSums(df4_3) != 0)
df4_4 <- df4_3[, i]
df4_5<- df4_4[rowSums(df4_4) != 0, ]
df4_6<-t(df4_5)
df4_7<-decostand(df4_6, method="total")
df4_7pa<-decostand(df4_6, method="pa")
df4_8<-data.frame(df4_7)
df4_8pa<-data.frame(df4_7)
```

```r
##### 2. calculating values RRA,POO,wPOO,FOO for 18S
Tuna18S_D=as.matrix(df1_6)
# How many seq per stomach?
Tuna18S_RC=apply(Tuna18S_D,2,sum);Tuna18S_RC;mean(Tuna18S_RC)
```

2. calculating values RRA,POO,wPOO,FOO for 18S

```
## M275 J290 J269 M281 M261 M198 M273 J233 J201 M280 J222 M118 M277 J221 M262 M279
##    5   37   10   84   25  908  286   80   60 1045  151  107   10  111  210  800
## J226  J53 J215 M117  M33  J47 J276  J52  M29 J138 J125 J121  M49 J144 J134 J150
##    3   51   32  497  240  721   39  194   35  142  122 1191  197   74  192  155
## J227  J57  M30  J46 J145  J96 J164 M123 M253 J229 J217 J205
##   53  119   71  571   62   30 3580  313  142  368   55   69
```

```
## [1] 301.0682
```

```r
write.csv(Tuna18S_RC,'Tuna18S_reads_per_stomach.csv')
# number of taxa against read depth
Tuna18S_TaxC=apply(Tuna18S_D,2,function(x) length(unique(x)))
plot(Tuna18S_RC,Tuna18S_TaxC)
```

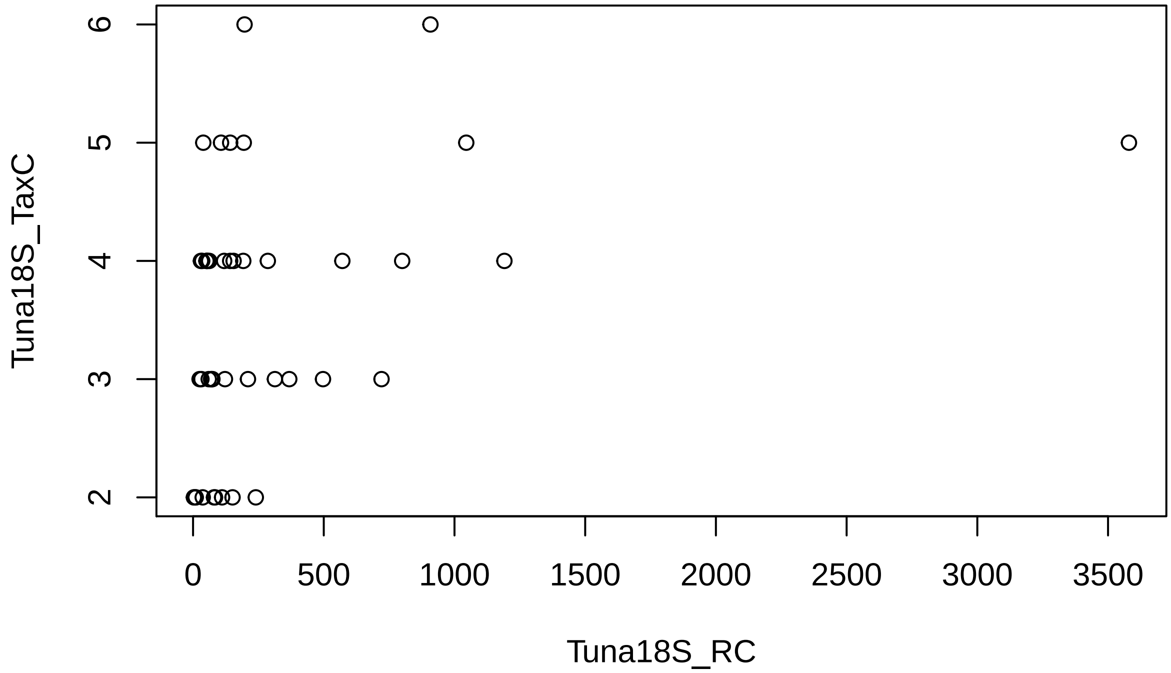

```
write.csv(Tuna18S_TaxC,'Tuna18S_taxa_per_stomach.csv')
#Turn into proportions, proportional datset
Tuna18S_Dprop=prop.table(Tuna18S_D,2)
dim(Tuna18S_Dprop)
```

```
## [1]  7 44
```

```
apply(Tuna18S_Dprop,2,sum)
```

```
## M275 J290 J269 M281 M261 M198 M273 J233 J201 M280 J222 M118 M277 J221 M262 M279
##    1    1    1    1    1    1    1    1    1    1    1    1    1    1    1    1
## J226  J53 J215 M117  M33  J47 J276  J52  M29 J138 J125 J121  M49 J144 J134 J150
##    1    1    1    1    1    1    1    1    1    1    1    1    1    1    1    1
## J227  J57  M30  J46 J145  J96 J164 M123 M253 J229 J217 J205
##    1    1    1    1    1    1    1    1    1    1    1    1
```

```
write.csv(Tuna18S_Dprop,'Tuna18S_proportions.csv')
# Make Presence absence (PA) dataset,
PA_Tuna18S_D= Tuna18S_Dprop
PA_Tuna18S_D[PA_Tuna18S_D>.0099999999]=1 ## here we fix the threshold for presence absence transformati
PA_Tuna18S_D[PA_Tuna18S_D<.01]=0 ## else 0
write.csv(Tuna18S_D,'Tuna18S_pres_abs.csv')
FOO_All_Tuna18S_=apply(PA_Tuna18S_D,1,sum)/length(PA_Tuna18S_D[1,])
Ord_Tuna18S_=order(-FOO_All_Tuna18S_)
write.csv(Ord_Tuna18S_,'Tuna18S_Foo.csv', row.names = TRUE)#
P_FOO_Tuna18S_=  apply(PA_Tuna18S_D,1,sum)/sum(apply(PA_Tuna18S_D,1,sum))
P_FOO_Tuna18S_
```

```
##        Annelida      Arthropoda        Cnidaria      Ctenophora Platyhelminthes
##      0.01886792      0.14150943      0.18867925      0.15094340      0.04716981
##        Tunicata      Vertebrata
##      0.16981132      0.28301887
```

```
sum(P_FOO_Tuna18S_)
```

```
## [1] 1
```

```
df_FOO_Tuna18S <- data.frame(P_FOO_Tuna18S_)
write.csv(df_FOO_Tuna18S,'Tuna18S_Poo.csv', row.names = TRUE)
barplot(FOO_All_Tuna18S_[Ord_Tuna18S_ ][1:15],ylim=c(0,1),col="blue",xaxt='n', xlab="% of samples with p
```

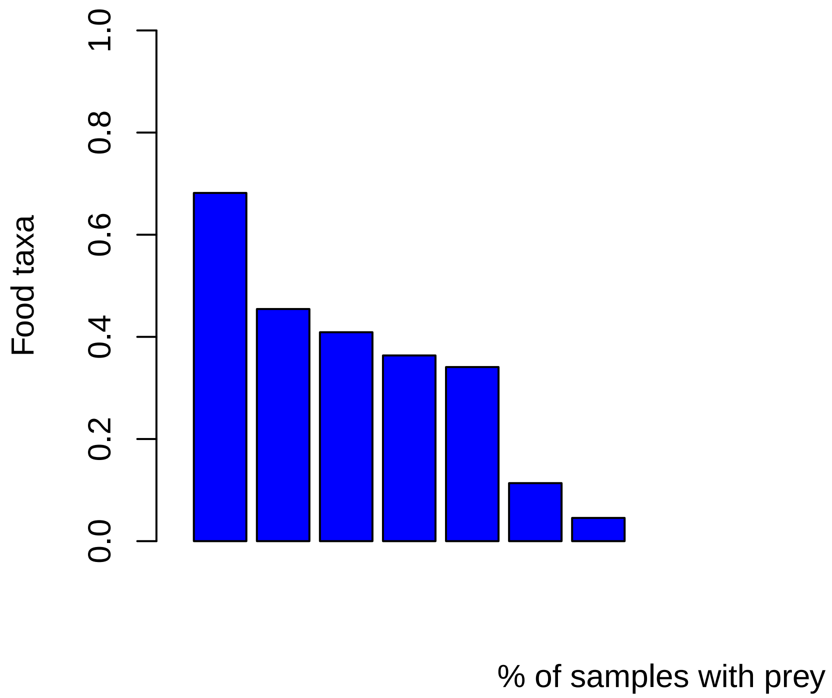


```
P_wFOO_Tuna18S_=apply(prop.table(PA_Tuna18S_D,2),1,mean,na.rm=T)
P_wFOO_Tuna18S_
```

```
##        Annelida      Arthropoda        Cnidaria      Ctenophora Platyhelminthes
##      0.01515152      0.12840909      0.17083333      0.15492424      0.03560606
##        Tunicata      Vertebrata
##      0.16136364      0.33371212
```

```
sum(P_wFOO_Tuna18S_)
```

```
## [1] 1
```

```
df_wFOO_Tuna18S <- data.frame(P_wFOO_Tuna18S_)
write.csv(df_wFOO_Tuna18S,'Tuna18S_wPoo.csv', row.names = TRUE)

P_RRA_Tuna18S_=apply(prop.table(as.matrix(Tuna18S_D),2),1,mean)
P_RRA_Tuna18S_
```

```
##        Annelida      Arthropoda        Cnidaria      Ctenophora Platyhelminthes
##     0.008775223     0.103940195     0.160764761     0.178458873     0.029076247
##        Tunicata      Vertebrata
##     0.151849864     0.367134838
```

```r
sum(P_RRA_Tuna18S_)
```

```
## [1] 1
```

```r
df_RRA_Tuna18S <- data.frame(P_RRA_Tuna18S_)
write.csv(df_RRA_Tuna18S,'Tuna18S_RRA.csv', row.names = TRUE)

plot(P_FOO_Tuna18S_[Ord_Tuna18S_][1:15],pch=15,col=2,type="b",ylim= c(0,0.3),ylab="% in Diet", xlab="Pr
points(16, sum(P_FOO_Tuna18S_[Ord_Tuna18S_][16:length(P_FOO_Tuna18S_)]),pch=15,col=2,cex=1.2 )
points(P_wFOO_Tuna18S_[Ord_Tuna18S_][1:15],pch=16,col=3,type="b")
points(16, sum(P_wFOO_Tuna18S_[Ord_Tuna18S_][16:length(P_wFOO_Tuna18S_)]),pch=16,col=3,cex=1.2 )
sum(P_wFOO_Tuna18S_[Ord_Tuna18S_][1:15])
```

```
## [1] NA
```

```r
points(P_RRA_Tuna18S_[Ord_Tuna18S_][1:15],pch=17,type="b")
points(16, sum(P_RRA_Tuna18S_[Ord_Tuna18S_][16:length(P_RRA_Tuna18S_)]),pch=17,cex=1.2 )
legend('top',c('POO','wPOO','RRA'),pch=c(15,16,17),col=c(2,3,1))
```

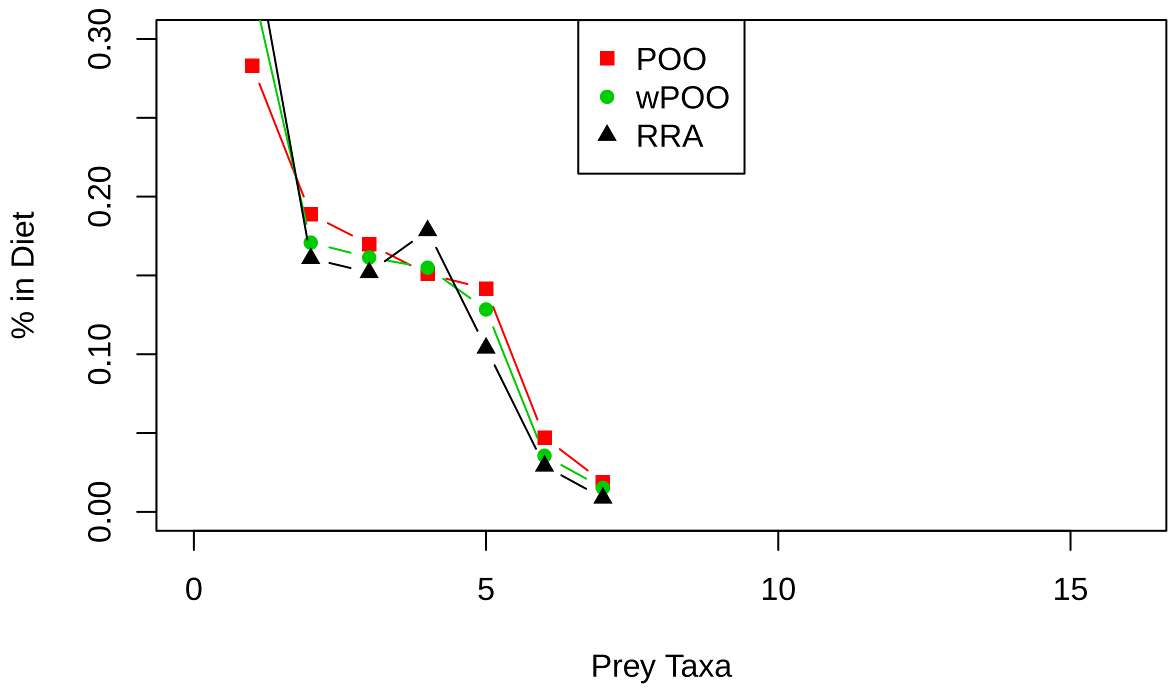


```r
#dev.new()
```

######3. calculating values RRA,POO,wPOO,FOO for COI

```r
########3. calculating values RRA,POO,wPOO,FOO for COI
TunaCOI_D=as.matrix(df2_5)
# How many seq per stomach?
TunaCOI_RC=apply(TunaCOI_D,2,sum);TunaCOI_RC;mean(TunaCOI_RC)
```

```
##  M275  J290  J269  M281  M261  M198  M273  J233  J201  M280  J222  M118  M277
##  2029   537  1480  1444   671  3630  6892  3051  3077  2764 13993  1399  8357
##  J221  M262  M279  J226   J53  J215  M117   M33   J47  J276   J52   M29  J138
## 17801  3374  1282  1068  4118  2835 10054  1036  5815  3284  4510   950  4643
##  J125  J121   M49  J144  J134  J150  J227   J57   M30   J46  J145   J96  J164
##   525   817   255  5104  3881 10269  5278   999   746  3317  5111  9454  3193
##  M123  M253  J229  J217  J205  J170
##  4261  3114  6592  3944 14440  5897
```

```
## [1] 4384.244
```

```r
write.csv(TunaCOI_RC,'TunaCOI_reads_per_stomach.csv')
# number of taxa against read depth
TunaCOI_TaxC=apply(TunaCOI_D,2,function(x) length(unique(x)))
plot(TunaCOI_RC,TunaCOI_TaxC)
```

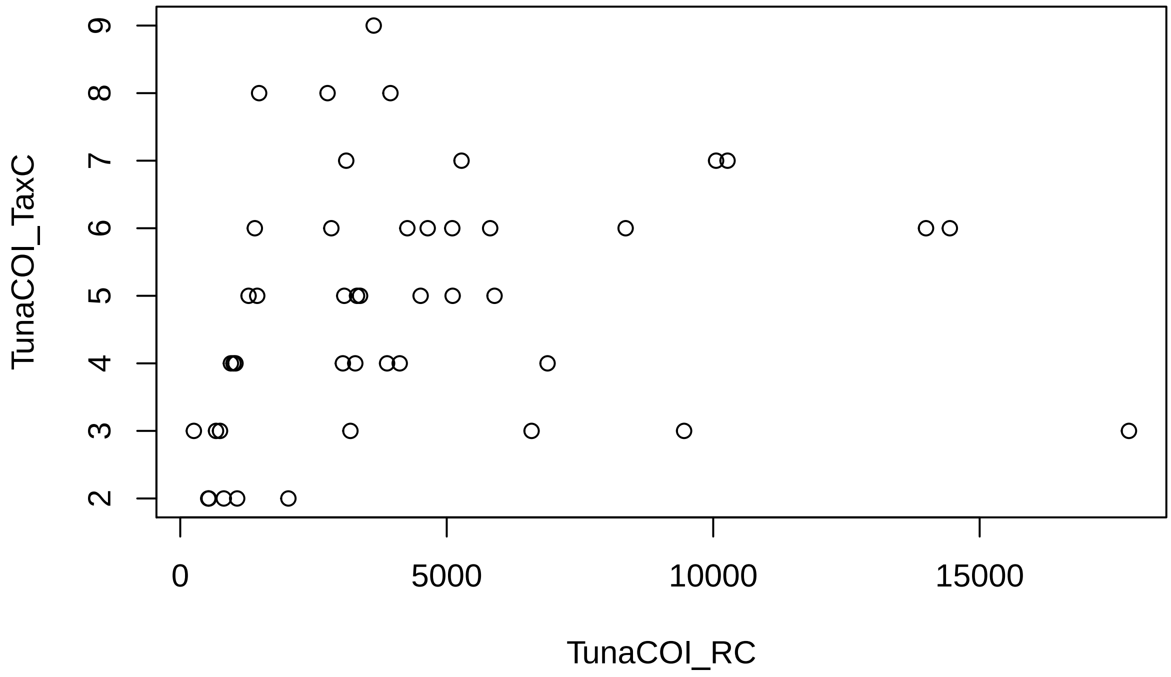


```r
write.csv(TunaCOI_TaxC,'TunaCOI_taxa_per_stomach.csv')
#Turn into proportions, proportional datset
TunaCOI_D
```

```
##                 M275 J290 J269 M281 M261 M198 M273 J233 J201 M280 J222 M118
## anchovy            0  537  406  882  569 1238 4740  311    0 2170 3859    0
## Arthropoda         0    0    6   17  102  100    0    0  361  167    0   51
## cephaloda          0    0    0    0    0    0    0    0    0    0    0    0
## Cepolaepola        0    0    0    0    0    0    0    0    0    0    0    0
## Chaetognatha       0    0    0    0    0   17    0    0    0    0    0    0
## Cnidaria           0    0   12    0    0   81    2    0    0   30    6    6
## Echinodermata      0    0    0    0    0    0    0    0    0    0    0    0
## mackerel           0    0  251    0    0    0    0    0    0    0    0  224
## Mollusca           0    0   23    0    0   26    0    0    0   16    0    0
## sardine         2029    0    0    0    0 2153 2150 1034 1556  351 8178 1103
## sprat              0    0  756  328    0    0    0 1706 1081    0 1863    0
## Tunicata           0    0    0    0    0    0    0    0    0    9    0    0
## Vertebrata         0    0   26  217    0   13    0    0   79   21   87   15
## Xenacoelomorpha    0    0    0    0    0    2    0    0    0    0    0    0
##                 M277  J221 M262 M279 J226  J53 J215 M117 M33  J47 J276  J52 M29
## anchovy         8030 11410 1799  800 1068 3088 2699 4161   0 4223 2498 2180 433
## Arthropoda       181     0   34    0    0    0  110    3   0    6    0    0   0
## cephaloda          0     0    0    0    0    0    0    0   0    0    0    0   0
## Cepolaepola        0     0    0    0    0    0    0    0   0    0    0    0   0
## Chaetognatha       0     0    0    0    0    0    0    0   0    0    0    0   0
## Cnidaria          39     0    0    2    0    0    5    0   0    0    0    0   0
## Echinodermata      0     0    0    0    0    0    9    0   0   17    0    0   0
## mackerel           0     0    0    0    0 1027    0    0   0 1474    0    0   0
## Mollusca          57     0   29    0    0    0   12   24   9   89    0   13   0
## sardine            0     0    0  383    0    0    0 5223 992    0  705 1634 424
## sprat              0  6391    0    0    0    0    0  564   0    0   81  683  93
## Tunicata           0     0    0    0    0    3    0    0   0    0    0    0   0
## Vertebrata        50     0 1512   97    0    0    0   79  35    6    0    0   0
## Xenacoelomorpha    0     0    0    0    0    0    0    0   0    0    0    0   0
##                 J138 J125 J121 M49 J144 J134 J150 J227 J57 M30  J46 J145  J96
## anchovy            0    0    0 242 3281 2833 2756 3063 677 723 1995 1847 8991
## Arthropoda       118    0    0   0    0    0   37    5   0   0    0   20    0
## cephaloda       2745    0    0   0    0    0    0    0   0   0    0    0    0
## Cepolaepola        0  525  817   0    0    0    0    0   0   0    0    0    0
## Chaetognatha       0    0    0   0    0    0    0    4   0   0    0    0    0
## Cnidaria           0    0    0   0    3    0    8    9   0   0    3    3    0
## Echinodermata      0    0    0   0    0    0    0    0   0   0    0    0    0
## mackerel         460    0    0  13  603 1045 3579  345 311   0 1027    0  463
## Mollusca           0    0    0   0    0    0    0    0   0   0    0    0    0
## sardine         1312    0    0   0  983    0 3161    0   0   0    0 3241    0
## sprat              0    0    0   0  234    0  728 1852   0  23  292    0    0
## Tunicata           0    0    0   0    0    0    0    0   0   0    0    0    0
## Vertebrata         8    0    0   0    0    3    0    0  11   0    0    0    0
## Xenacoelomorpha    0    0    0   0    0    0    0    0   0   0    0    0    0
##                 J164 M123 M253 J229 J217 J205 J170
## anchovy          784 1599    0 2983  449 5173 2722
## Arthropoda         0    0 1006    0  269   75    0
## cephaloda          0    0    0    0    0    0    0
## Cepolaepola        0    0    0    0    0    0    0
## Chaetognatha       0    0    6    0    0    0    0
## Cnidaria           0    0    0    0    6   13    0
## Echinodermata      0    0    0    0    0    0    0
## mackerel           0  728    0    0    0    0    0
```

```
## Mollusca            0    0    4    0    0    0   16
## sardine          2409 1812 1733 3609 2496 8098    0
## sprat               0  115  360    0  680 1068 3123
## Tunicata            0    0    0    0    0    0    0
## Vertebrata          0    7    5    0   24   13   36
## Xenacoelomorpha     0    0    0    0   20    0    0
```

```r
TunaCOI_Dprop=prop.table(TunaCOI_D,2)
dim(TunaCOI_Dprop)
```

```
## [1] 14 45
```

```r
apply(TunaCOI_Dprop,2,sum)
```

```
## M275 J290 J269 M281 M261 M198 M273 J233 J201 M280 J222 M118 M277 J221 M262 M279
##    1    1    1    1    1    1    1    1    1    1    1    1    1    1    1    1
## J226  J53 J215 M117  M33  J47 J276  J52  M29 J138 J125 J121  M49 J144 J134 J150
##    1    1    1    1    1    1    1    1    1    1    1    1    1    1    1    1
## J227  J57  M30  J46 J145  J96 J164 M123 M253 J229 J217 J205 J170
##    1    1    1    1    1    1    1    1    1    1    1    1    1
```

```r
write.csv(TunaCOI_Dprop,'TunaCOI_proportions.csv')
# Make Presence absence (PA) dataset,
PA_TunaCOI_D= TunaCOI_Dprop
PA_TunaCOI_D[PA_TunaCOI_D>.0099999999]=1 ## here we fix the threshold for presence absence transformati
PA_TunaCOI_D[PA_TunaCOI_D<.01]=0 ## else 0
write.csv(TunaCOI_D,'TunaCOI_pres_abs.csv')
FOO_All_TunaCOI_=apply(PA_TunaCOI_D,1,sum)/length(PA_TunaCOI_D[1,])
Ord_TunaCOI_=order(-FOO_All_TunaCOI_)
write.csv(Ord_TunaCOI_,'TunaCOI_Foo.csv', row.names = TRUE)
barplot(FOO_All_TunaCOI_[Ord_TunaCOI_ ][1:15],ylim=c(0,1),col="blue",xaxt='n', xlab="% of samples with
```

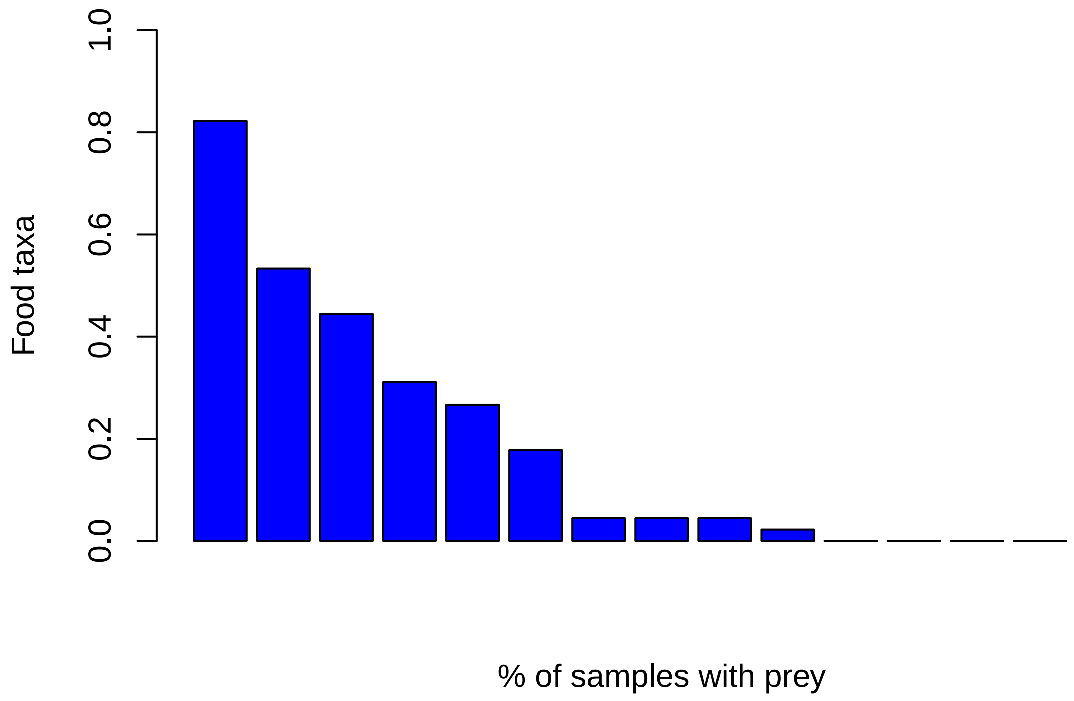


```
P_FOO_TunaCOI_=  apply(PA_TunaCOI_D,1,sum)/sum(apply(PA_TunaCOI_D,1,sum))
P_FOO_TunaCOI_
```

```
##         anchovy      Arthropoda       cephaloda     Cepolaepola    Chaetognatha
##     0.303278689     0.098360656     0.008196721     0.016393443     0.000000000
##        Cnidaria  Echinodermata        mackerel        Mollusca         sardine
##     0.016393443     0.000000000     0.114754098     0.016393443     0.196721311
##           sprat        Tunicata      Vertebrata Xenacoelomorpha
##     0.163934426     0.000000000     0.065573770     0.000000000
```

```
sum(P_FOO_TunaCOI_)
```

```
## [1] 1
```

```
df_FOO_TunaCOI <- data.frame(P_FOO_TunaCOI_)
write.csv(df_FOO_TunaCOI,'TunaCOI_Poo.csv', row.names = TRUE)

P_wFOO_TunaCOI_=apply(prop.table(PA_TunaCOI_D,2),1,mean,na.rm=T)
P_wFOO_TunaCOI_
```

```
##         anchovy      Arthropoda       cephaloda     Cepolaepola    Chaetognatha
##     0.339629630     0.087037037     0.005555556     0.044444444     0.000000000
##        Cnidaria  Echinodermata        mackerel        Mollusca         sardine
##     0.011111111     0.000000000     0.106296296     0.011851852     0.194444444
```

```
##           sprat        Tunicata      Vertebrata Xenacoelomorpha
##     0.145185185     0.000000000     0.054444444     0.000000000
```

```
sum(P_wFOO_TunaCOI_)
```

```
## [1] 1
```

```
df_wFOO_TunaCOI <- data.frame(P_wFOO_TunaCOI_)
write.csv(df_wFOO_TunaCOI,'TunaCOI_wPoo.csv', row.names = TRUE)

P_RRA_TunaCOI_=apply(prop.table(as.matrix(TunaCOI_D),2),1,mean)
P_RRA_TunaCOI_
```

```
##         anchovy      Arthropoda       cephaloda     Cepolaepola    Chaetognatha
##    4.880851e-01    2.026187e-02    1.313806e-02    4.444444e-02    1.637298e-04
##        Cnidaria  Echinodermata        mackerel        Mollusca         sardine
##    1.361316e-03    1.355128e-04    5.832636e-02    1.808894e-03    2.654016e-01
##           sprat        Tunicata      Vertebrata Xenacoelomorpha
##    8.835000e-02    8.854799e-05    1.830967e-02    1.249324e-04
```

```
sum(P_RRA_TunaCOI_)
```

```
## [1] 1
```

```
df_RRA_TunaCOI <- data.frame(P_RRA_TunaCOI_)
write.csv(df_RRA_TunaCOI,'TunaCOI_RRA.csv', row.names = TRUE)

plot(P_FOO_TunaCOI_[Ord_TunaCOI_][1:15],pch=15,col=2,type="b",ylim= c(0,0.3),ylab="% in Diet", xlab="Pr
points(16, sum(P_FOO_TunaCOI_[Ord_TunaCOI_][16:length(P_FOO_TunaCOI_)]),pch=15,col=2,cex=1.2 )
points(P_wFOO_TunaCOI_[Ord_TunaCOI_][1:15],pch=16,col=3,type="b")
points(16, sum(P_wFOO_TunaCOI_[Ord_TunaCOI_][16:length(P_wFOO_TunaCOI_)]),pch=16,col=3,cex=1.2 )
sum(P_wFOO_TunaCOI_[Ord_TunaCOI_][1:15])
```

```
## [1] NA
```

```
points(P_RRA_TunaCOI_[Ord_TunaCOI_][1:15],pch=17,type="b")
points(16, sum(P_RRA_TunaCOI_[Ord_TunaCOI_][16:length(P_RRA_TunaCOI_)]),pch=17,cex=1.2 )
legend('top',c('POO','wPOO','RRA'),pch=c(15,16,17),col=c(2,3,1))
```

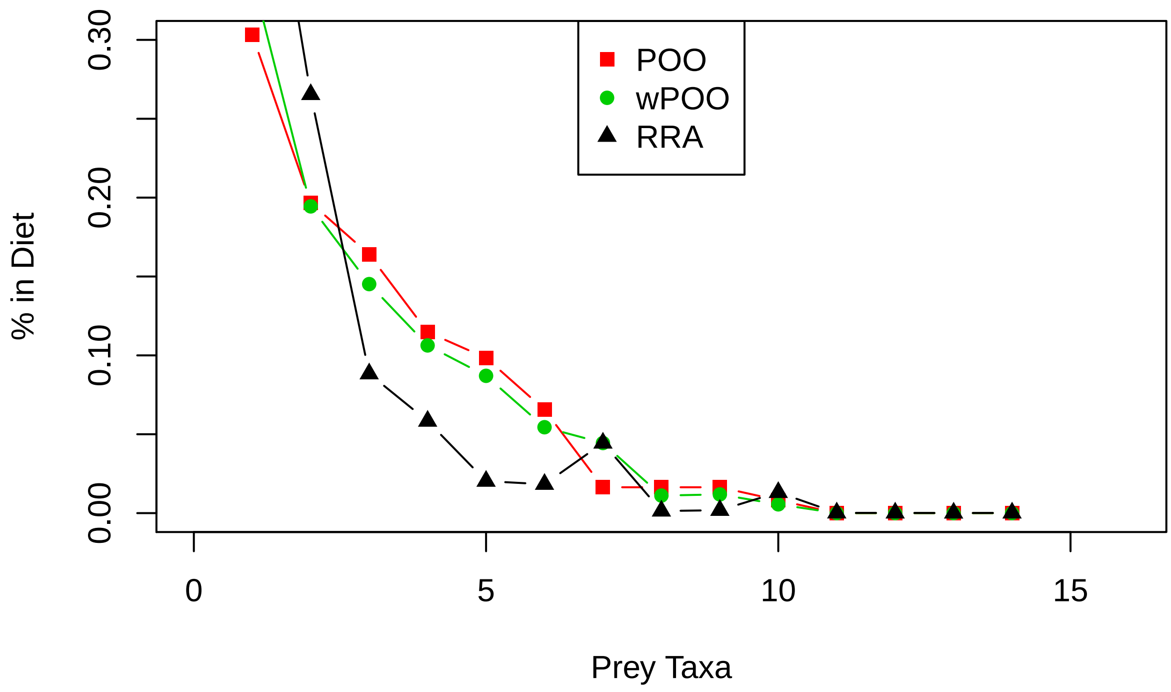


```
##### 4. RRA PER YEAR TO COMPARE TO MOPHOLOGICAL RA, relative abundance data
df1_7<-t(df1_6)
df2_6<-t(df2_5)
S182011 <- subset(df1_7,dm2$Year == 2011)
S18_2011<-t(S182011)
S182012 <- subset(df1_7,dm2$Year == 2012)
S18_2012<-t(S182012)
S182013 <- subset(df1_7,dm2$Year == 2013)
S18_2013<-t(S182013)
S182014 <- subset(df1_7,dm2$Year == 2014)
S18_2014<-t(S182014)
COI2011 <- subset(df2_6,dm$Year == 2011)
COI_2011<-t(COI2011)
COI2012 <- subset(df2_6,dm$Year == 2012)
COI_2012<-t(COI2012)
COI2013 <- subset(df2_6,dm$Year == 2013)
COI_2013<-t(COI2013)
COI2014 <- subset(df2_6,dm$Year == 2014)
COI_2014<-t(COI2014)

RRA_2011_18S=apply(prop.table(as.matrix(S18_2011),2),1,mean)
RRA_2012_18S=apply(prop.table(as.matrix(S18_2012),2),1,mean)
RRA_2013_18S=apply(prop.table(as.matrix(S18_2013),2),1,mean)
```

```
RRA_2014_18S=apply(prop.table(as.matrix(S18_2014),2),1,mean)
RRA_2011_COI=apply(prop.table(as.matrix(COI_2011),2),1,mean)
RRA_2012_COI=apply(prop.table(as.matrix(COI_2012),2),1,mean)
RRA_2013_COI=apply(prop.table(as.matrix(COI_2013),2),1,mean)
RRA_2014_COI=apply(prop.table(as.matrix(COI_2014),2),1,mean)
RRA_18S_year <- cbind (RRA_2011_18S, RRA_2012_18S, RRA_2013_18S, RRA_2014_18S)
colnames(RRA_18S_year) <- c("RRA_2011","RRA_2012","RRA_2013","RRA_2014")
write.csv(RRA_18S_year,'RRA_Year_18S.csv', row.names = TRUE)
RRA_COI_year.all <- cbind (RRA_2011_COI, RRA_2012_COI, RRA_2013_COI, RRA_2014_COI)
colnames(RRA_COI_year.all) <- c("RRA_2011","RRA_2012","RRA_2013","RRA_2014")
RRA_COI_year<- data.frame(RRA_COI_year.all)
write.csv(RRA_COI_year.all,'RRA_Year_COI.csv', row.names = TRUE)

#include morphological data
RRA_COI_year<- cbind (RRA_2011_COI, RRA_2012_COI, RRA_2013_COI)
colnames(RRA_COI_year) <- c("RRA_2011","RRA_2012","RRA_2013")
colnames(morp) <- c("RA_2011","RA_2012","RA_2013")
morp<- data.frame(morp)
RRA_COI_year<- data.frame(RRA_COI_year)
Metazoa_verg<-merge(RRA_COI_year,morp,by="row.names",all.x=TRUE)

###T test,
t.test(Metazoa_verg$RRA_2011,Metazoa_verg$RA_2011)
```

4. RRA PER YEAR TO COMPARE TO MOPHOLOGICAL RA, relative abundance data

```
##
##  Welch Two Sample t-test
##
## data:  Metazoa_verg$RRA_2011 and Metazoa_verg$RA_2011
## t = -0.92498, df = 3.2972, p-value = 0.4176
## alternative hypothesis: true difference in means is not equal to 0
## 95 percent confidence interval:
##  -0.7093934  0.3772505
## sample estimates:
##  mean of x  mean of y
## 0.07142857 0.23750000
```

```
t.test(Metazoa_verg$RRA_2012,Metazoa_verg$RA_2012)
```

```
##
##  Welch Two Sample t-test
##
## data:  Metazoa_verg$RRA_2012 and Metazoa_verg$RA_2012
## t = -1.3235, df = 3.5262, p-value = 0.2649
## alternative hypothesis: true difference in means is not equal to 0
## 95 percent confidence interval:
##  -0.5417421  0.2045993
## sample estimates:
##  mean of x  mean of y
## 0.07142857 0.24000000
```

```
t.test(Metazoa_verg$RRA_2013,Metazoa_verg$RA_2013)
```

```
##
##  Welch Two Sample t-test
##
## data:  Metazoa_verg$RRA_2013 and Metazoa_verg$RA_2013
## t = -1.5372, df = 4.45, p-value = 0.1919
## alternative hypothesis: true difference in means is not equal to 0
## 95 percent confidence interval:
##  -0.4612733  0.1241304
## sample estimates:
##  mean of x  mean of y
## 0.07142857 0.24000000
```

```
t.test(RRA_COI_year,morp)
```

```
##
##  Welch Two Sample t-test
##
## data:  RRA_COI_year and morp
## t = -1.6405, df = 23.627, p-value = 0.1142
## alternative hypothesis: true difference in means is not equal to 0
## 95 percent confidence interval:
##  -0.21390384  0.02453876
## sample estimates:
##  mean of x  mean of y
## 0.07142857 0.16611111
```

```
Metazoa_verg.all<-merge(RRA_COI_year.all,morp,by="row.names",all.x=TRUE)
write.csv(Metazoa_verg.all,'RRA_RA_compPerYear.csv', row.names = TRUE)

##
```

```
##### 5.Statistical distribution tests with euclidean distance/Sorensen dissimilarity, based on OTU lev

#Euclidian 18S
euc.18S =vegdist(df3_8,binary=TRUE)
anosim(euc.18S, dm2$Weight)
```

```
##
## Call:
## anosim(x = euc.18S, grouping = dm2$Weight)
## Dissimilarity: binary bray
##
## ANOSIM statistic R: 0.332
##       Significance: 0.037
##
## Permutation: free
## Number of permutations: 999
```

```r
anosim(euc.18S, dm2$code.port)
```

```
##
## Call:
## anosim(x = euc.18S, grouping = dm2$code.port)
## Dissimilarity: binary bray
##
## ANOSIM statistic R: -0.0007829
##       Significance: 0.486
##
## Permutation: free
## Number of permutations: 999
```

```r
anosim(euc.18S, dm2$Longueur_Bouche)
```

```
##
## Call:
## anosim(x = euc.18S, grouping = dm2$Longueur_Bouche)
## Dissimilarity: binary bray
##
## ANOSIM statistic R: -0.1191
##       Significance: 0.84
##
## Permutation: free
## Number of permutations: 999
```

```r
anosim(euc.18S, dm2$Sexe)
```

```
##
## Call:
## anosim(x = euc.18S, grouping = dm2$Sexe)
## Dissimilarity: binary bray
##
## ANOSIM statistic R: -0.01499
##       Significance: 0.621
##
## Permutation: free
## Number of permutations: 999
```

```r
anosim(euc.18S, dm2$Reproductive.Stage)
```

```
##
## Call:
## anosim(x = euc.18S, grouping = dm2$Reproductive.Stage)
## Dissimilarity: binary bray
##
## ANOSIM statistic R: -0.02501
##       Significance: 0.649
##
## Permutation: free
## Number of permutations: 999
```

```r
anosim(euc.18S, dm2$SIZE_CLASS)
```

```
##
## Call:
## anosim(x = euc.18S, grouping = dm2$SIZE_CLASS)
## Dissimilarity: binary bray
##
## ANOSIM statistic R: -0.06999
##       Significance: 0.991
##
## Permutation: free
## Number of permutations: 999
```

```r
anosim(euc.18S, dm2$Year)
```

```
##
## Call:
## anosim(x = euc.18S, grouping = dm2$Year)
## Dissimilarity: binary bray
##
## ANOSIM statistic R: 0.01035
##       Significance: 0.371
##
## Permutation: free
## Number of permutations: 999
```

```r
Weight_18S.euc<- betadisper(euc.18S, dm2$Weight, type = c("centroid"))
Port_18S.euc<- betadisper(euc.18S, dm2$code.port, type = c("centroid"))
mouth.Lengt_18S.euc<- betadisper(euc.18S, dm2$Longueur_Bouche, type = c("centroid"))
Sex_18S.euc<- betadisper(euc.18S, dm2$Sexe, type = c("centroid"))
rep.stage_18S.euc<- betadisper(euc.18S, dm2$Reproductive.Stage, type = c("centroid"))
size.class_18S.euc<- betadisper(euc.18S, dm2$SIZE_CLASS, type = c("centroid"))
Year_18S.euc<- betadisper(euc.18S, dm2$Year, type = c("centroid"))
anova(Weight_18S.euc)
```

```
## Analysis of Variance Table
##
## Response: Distances
##           Df  Sum Sq  Mean Sq F value  Pr(>F)
## Groups    36 1.64314 0.045643  15.714 0.00046 ***
## Residuals  7 0.02033 0.002905
## ---
## Signif. codes:  0 '***' 0.001 '**' 0.01 '*' 0.05 '.' 0.1 ' ' 1
```

```r
plot (Weight_18S.euc)
```

```
## Warning in plot.xy(xy.coords(x, y), type = type, ...): valeur '26' de pch non
## implémentée

## Warning in plot.xy(xy.coords(x, y), type = type, ...): valeur '31' de pch non
## implémentée
```

```
## Warning in plot.xy(xy.coords(x, y), type = type, ...): valeur '30' de pch non
## implémentée

## Warning in plot.xy(xy.coords(x, y), type = type, ...): valeur '27' de pch non
## implémentée

## Warning in plot.xy(xy.coords(x, y), type = type, ...): valeur '29' de pch non
## implémentée

## Warning in plot.xy(xy.coords(x, y), type = type, ...): valeur '28' de pch non
## implémentée
```

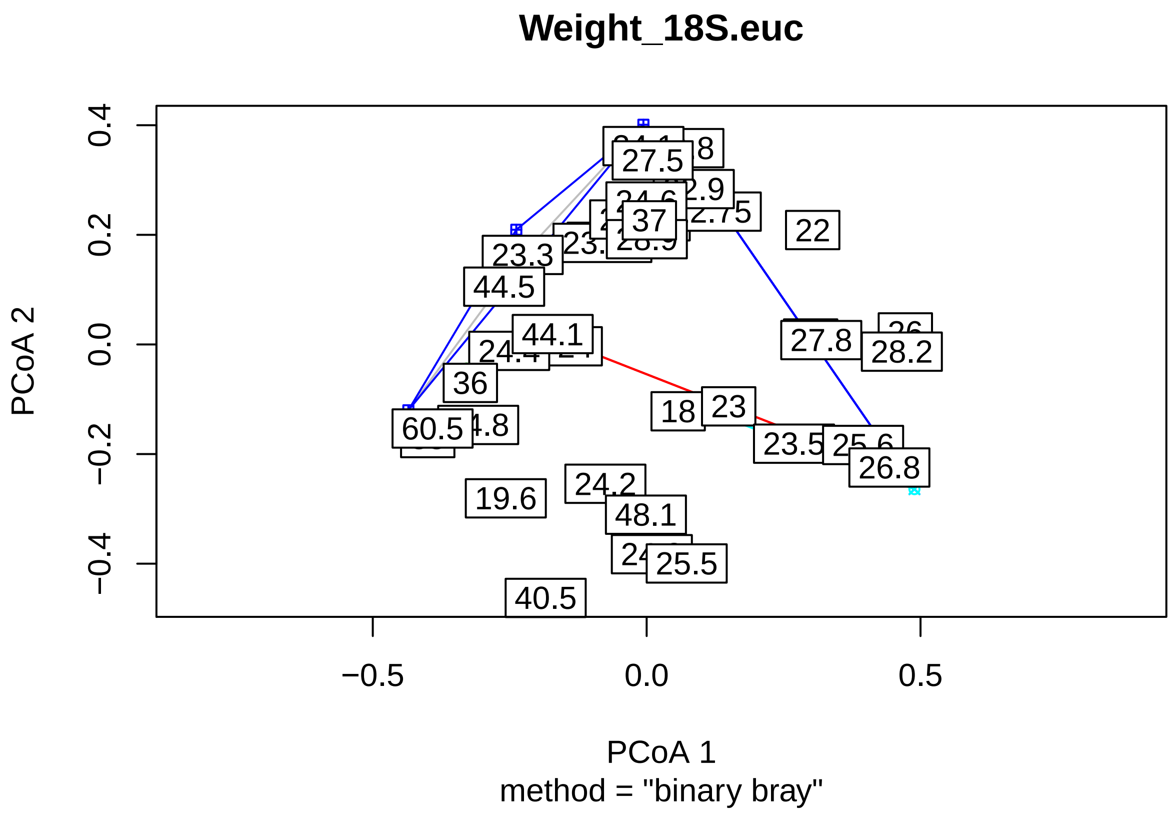


```r
anova(Port_18S.euc)
```

```
## Analysis of Variance Table
##
## Response: Distances
##           Df   Sum Sq   Mean Sq F value Pr(>F)
## Groups     3 0.029892 0.0099641  1.8234 0.1584
## Residuals 40 0.218589 0.0054647
```

```r
plot(Port_18S.euc)
```

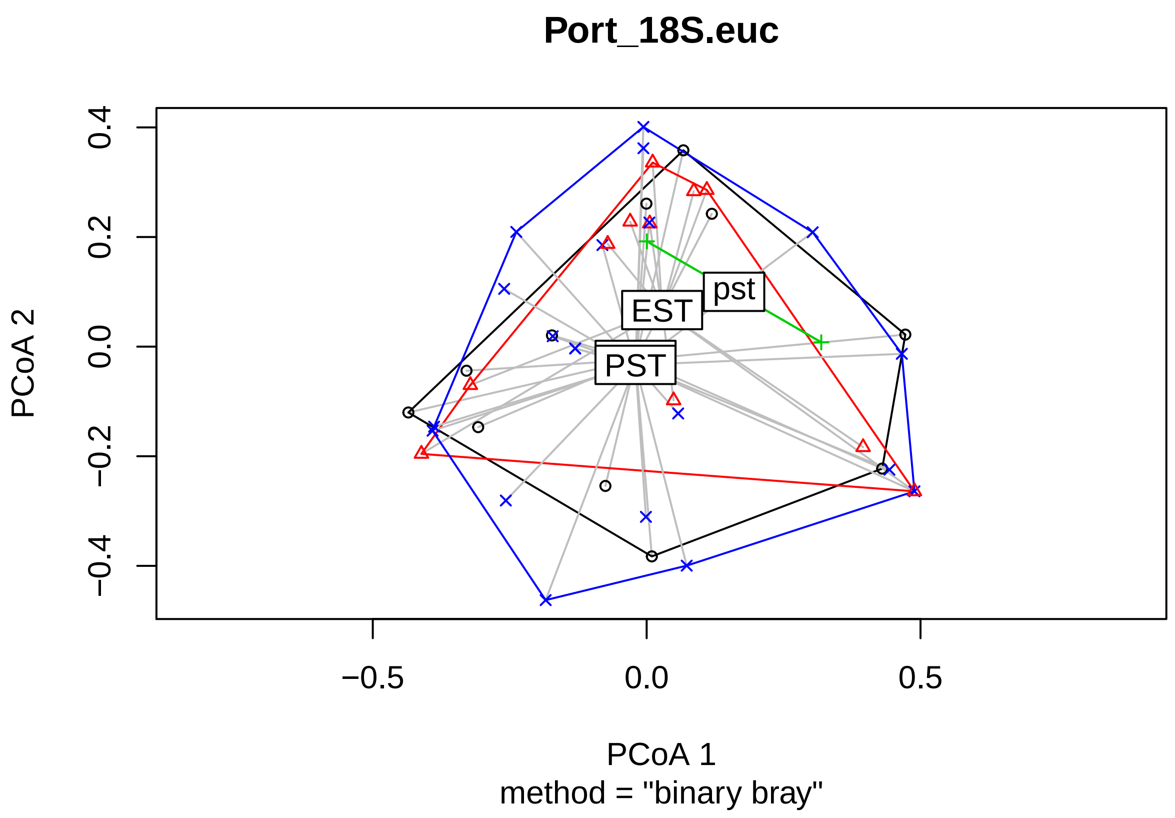


```r
anova(mouth.Lengt_18S.euc)
```

```
## Analysis of Variance Table
##
## Response: Distances
##           Df  Sum Sq  Mean Sq F value    Pr(>F)
## Groups    29 2.86170 0.098679  74.153 4.178e-11 ***
## Residuals 14 0.01863 0.001331
## ---
## Signif. codes:  0 '***' 0.001 '**' 0.01 '*' 0.05 '.' 0.1 ' ' 1
```

```r
plot(mouth.Lengt_18S.euc)
```

```
## Warning in plot.xy(xy.coords(x, y), type = type, ...): valeur '29' de pch non
## implémentée

## Warning in plot.xy(xy.coords(x, y), type = type, ...): valeur '26' de pch non
## implémentée

## Warning in plot.xy(xy.coords(x, y), type = type, ...): valeur '30' de pch non
## implémentée

## Warning in plot.xy(xy.coords(x, y), type = type, ...): valeur '26' de pch non
## implémentée
```

```
## Warning in plot.xy(xy.coords(x, y), type = type, ...): valeur '28' de pch non
## implémentée

## Warning in plot.xy(xy.coords(x, y), type = type, ...): valeur '27' de pch non
## implémentée
```

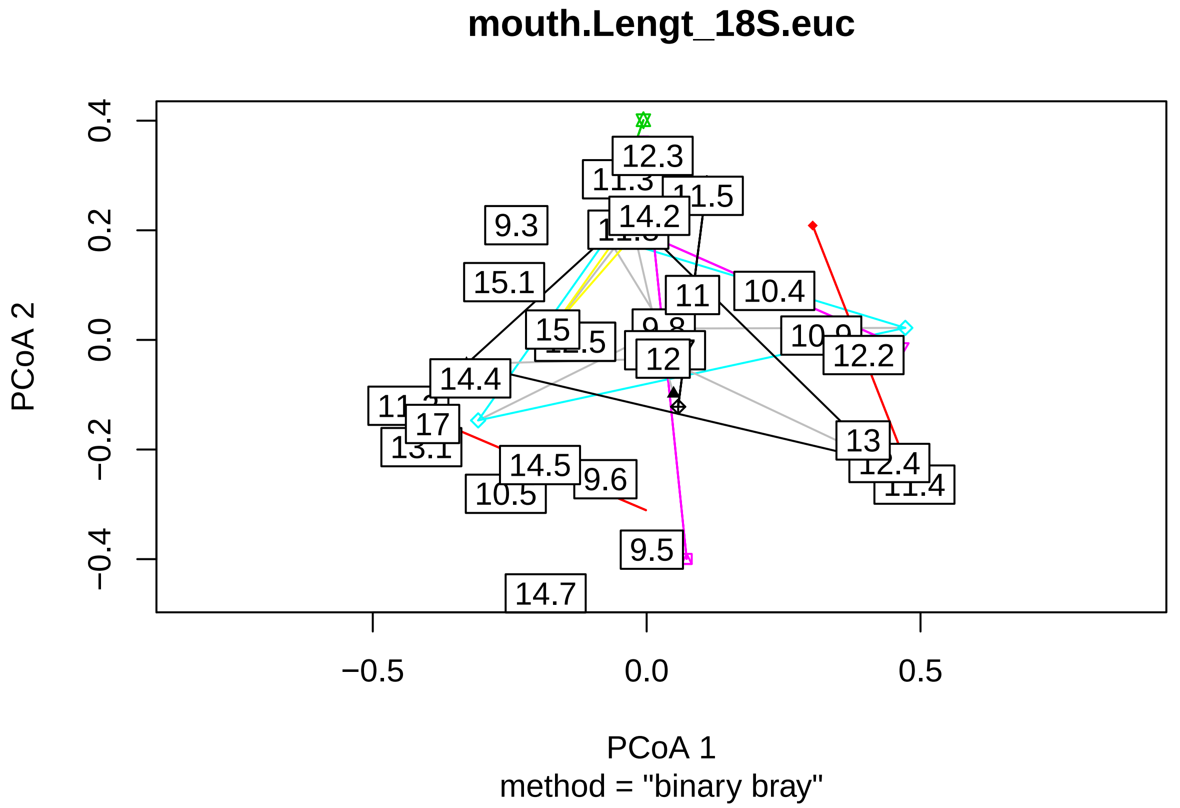


```r
anova(Sex_18S.euc)
```

```
## Analysis of Variance Table
##
## Response: Distances
##           Df   Sum Sq   Mean Sq F value  Pr(>F)
## Groups     2 0.038222 0.0191109  3.3269 0.04582 *
## Residuals 41 0.235520 0.0057444
## ---
## Signif. codes:  0 '***' 0.001 '**' 0.01 '*' 0.05 '.' 0.1 ' ' 1
```

```r
plot(Sex_18S.euc)
```

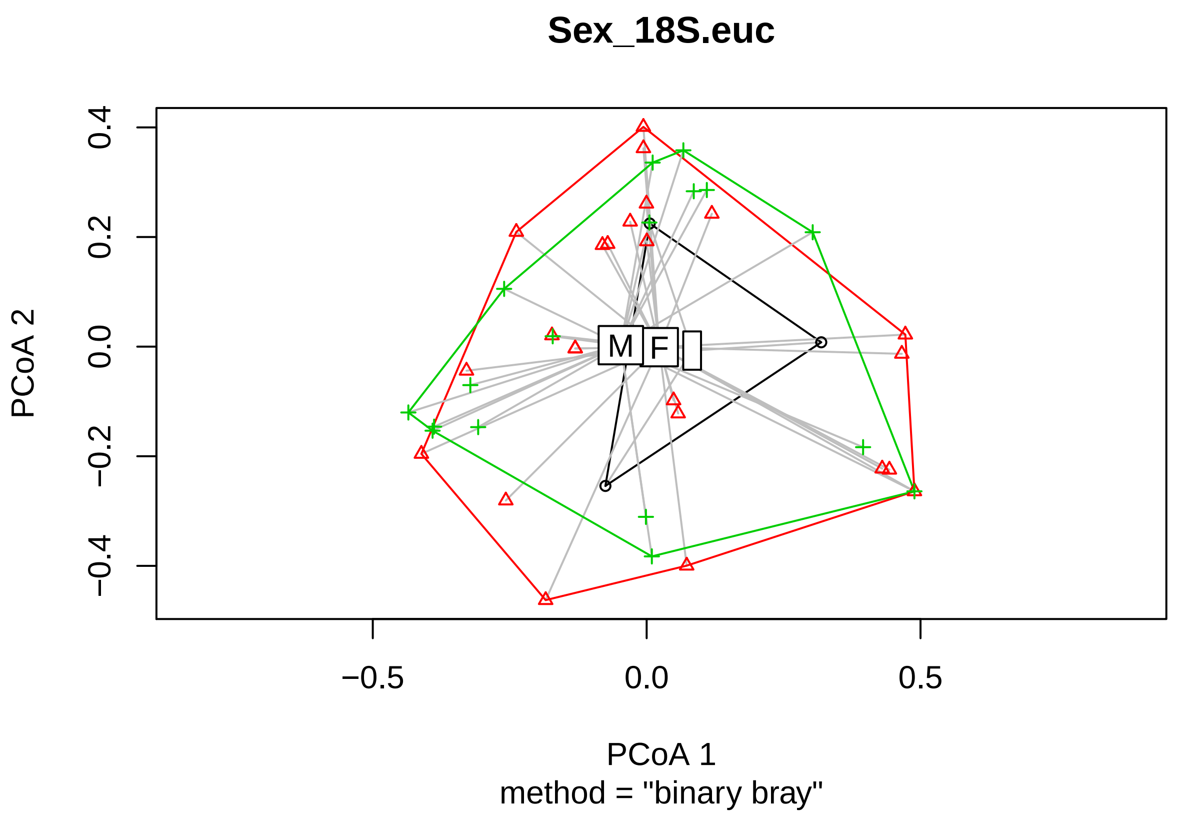


```r
anova(rep.stage_18S.euc)
```

```
## Analysis of Variance Table
##
## Response: Distances
##           Df   Sum Sq   Mean Sq F value Pr(>F)
## Groups     3 0.058879 0.0196264  3.6315 0.0208 *
## Residuals 40 0.216182 0.0054046
## ---
## Signif. codes:  0 '***' 0.001 '**' 0.01 '*' 0.05 '.' 0.1 ' ' 1
```

```r
plot(rep.stage_18S.euc)
```

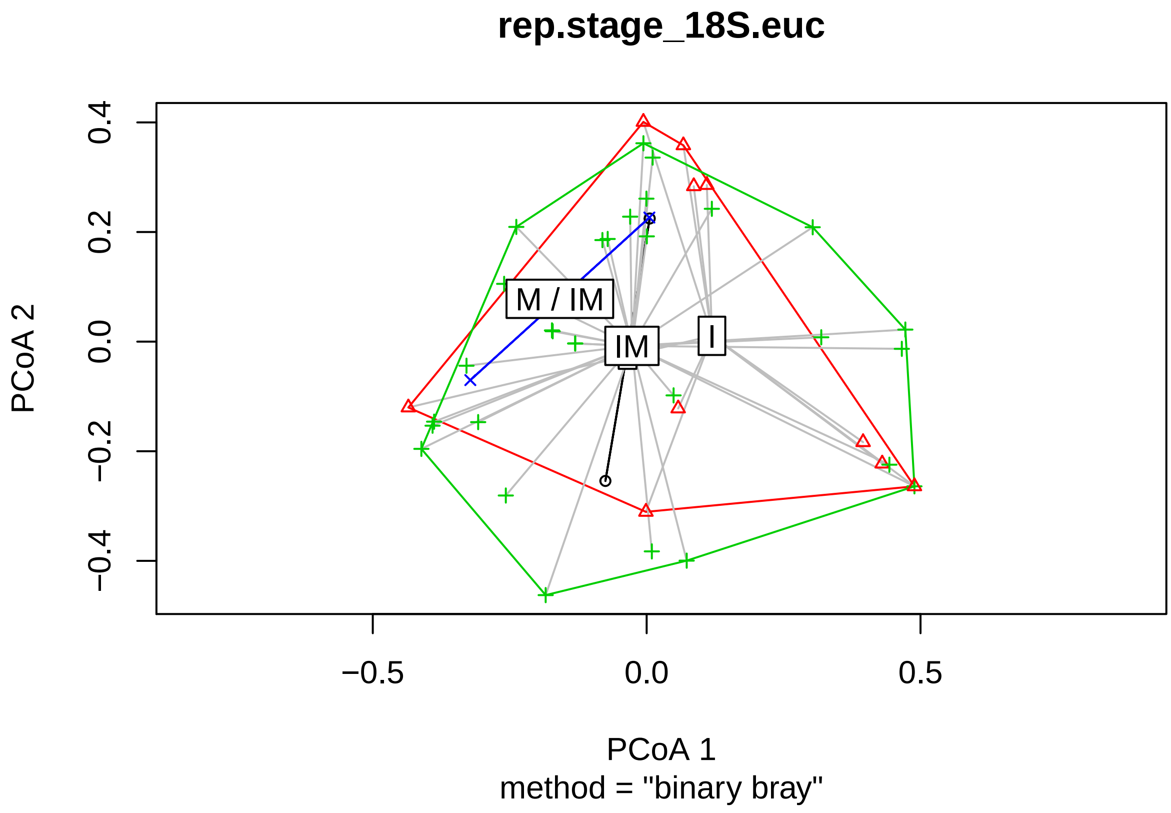


```r
anova(size.class_18S.euc)
```

```
## Analysis of Variance Table
##
## Response: Distances
##           Df   Sum Sq  Mean Sq F value  Pr(>F)
## Groups     1 0.038615 0.038615  6.3065 0.01596 *
## Residuals 42 0.257169 0.006123
## ---
## Signif. codes:  0 '***' 0.001 '**' 0.01 '*' 0.05 '.' 0.1 ' ' 1
```

```r
plot(size.class_18S.euc)
```

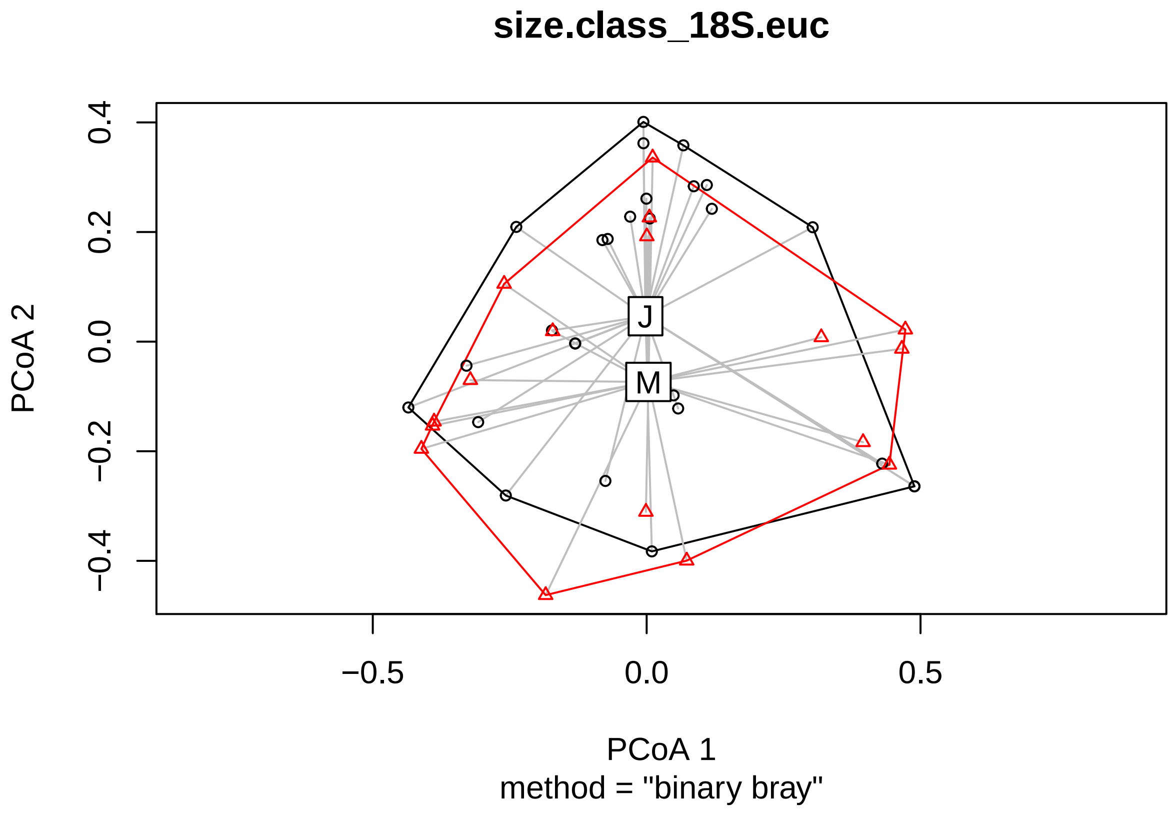


```r
anova(Year_18S.euc)
```

```
## Analysis of Variance Table
##
## Response: Distances
##           Df   Sum Sq   Mean Sq F value Pr(>F)
## Groups     3 0.023615 0.0078718  1.1881 0.3265
## Residuals 40 0.265013 0.0066253
```

```r
plot(Year_18S.euc)
```

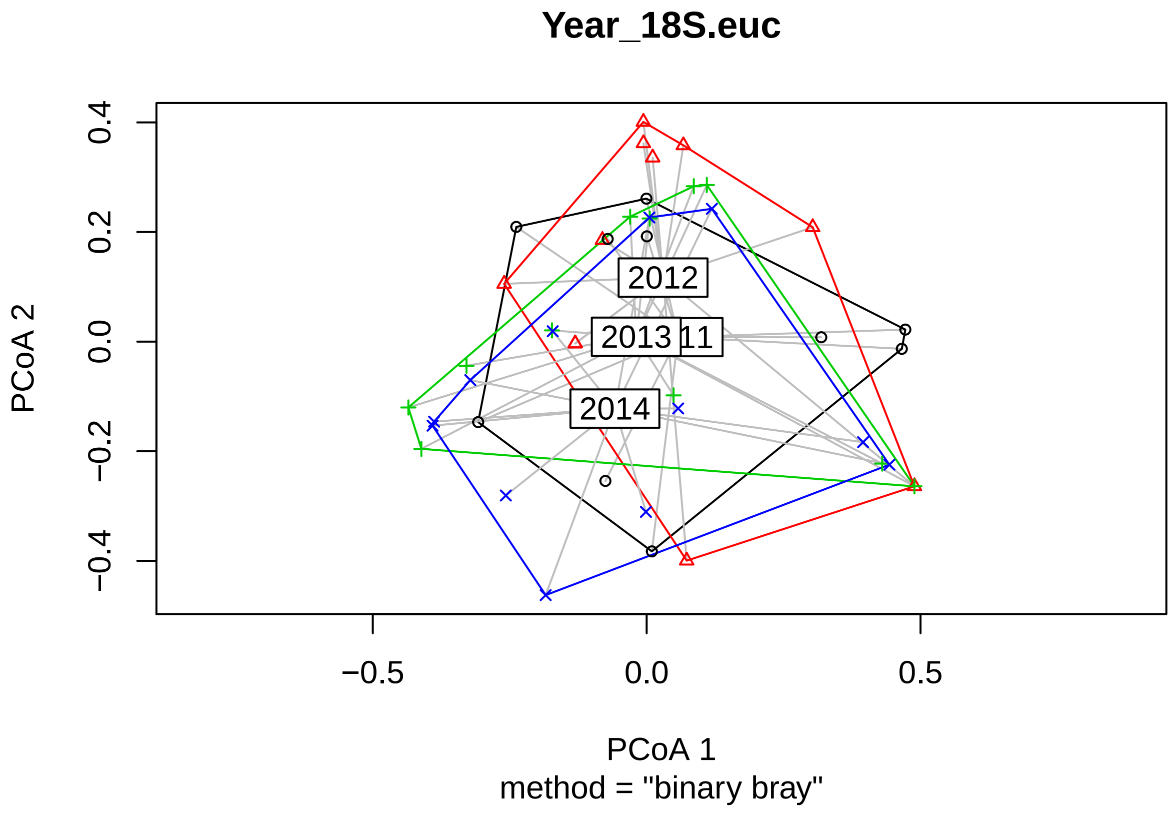


```r
#####Sorensen 18S
sor.18S =vegdist(df3_8pa,binary=TRUE)
anosim(sor.18S, dm2$Weight)
```

```
##
## Call:
## anosim(x = sor.18S, grouping = dm2$Weight)
## Dissimilarity: binary bray
##
## ANOSIM statistic R: 0.332
##       Significance: 0.057
##
## Permutation: free
## Number of permutations: 999
```

```r
anosim(sor.18S, dm2$code.port)
```

```
##
## Call:
## anosim(x = sor.18S, grouping = dm2$code.port)
## Dissimilarity: binary bray
##
## ANOSIM statistic R: -0.0007829
##       Significance: 0.47
##
```

```
## Permutation: free
## Number of permutations: 999
```

```r
anosim(sor.18S, dm2$Longueur_Bouche)
```

```
##
## Call:
## anosim(x = sor.18S, grouping = dm2$Longueur_Bouche)
## Dissimilarity: binary bray
##
## ANOSIM statistic R: -0.1191
##       Significance: 0.852
##
## Permutation: free
## Number of permutations: 999
```

```r
anosim(sor.18S, dm2$Sexe)
```

```
##
## Call:
## anosim(x = sor.18S, grouping = dm2$Sexe)
## Dissimilarity: binary bray
##
## ANOSIM statistic R: -0.01499
##       Significance: 0.646
##
## Permutation: free
## Number of permutations: 999
```

```r
anosim(sor.18S, dm2$Reproductive.Stage)
```

```
##
## Call:
## anosim(x = sor.18S, grouping = dm2$Reproductive.Stage)
## Dissimilarity: binary bray
##
## ANOSIM statistic R: -0.02501
##       Significance: 0.63
##
## Permutation: free
## Number of permutations: 999
```

```r
anosim(sor.18S, dm2$SIZE_CLASS)
```

```
##
## Call:
## anosim(x = sor.18S, grouping = dm2$SIZE_CLASS)
## Dissimilarity: binary bray
##
## ANOSIM statistic R: -0.06999
##       Significance: 0.993
```

```
##
## Permutation: free
## Number of permutations: 999
```

```
anosim(sor.18S, dm2$Year)
```

```
##
## Call:
## anosim(x = sor.18S, grouping = dm2$Year)
## Dissimilarity: binary bray
##
## ANOSIM statistic R: 0.01035
##       Significance: 0.369
##
## Permutation: free
## Number of permutations: 999
```

```
Weight_18S.sor<- betadisper(sor.18S, dm2$Weight, type = c("centroid"))
Port_18S.sor<- betadisper(sor.18S, dm2$code.port, type = c("centroid"))
mouth.Lengt_18S.sor<- betadisper(sor.18S, dm2$Longueur_Bouche, type = c("centroid"))
Sex_18S.sor<- betadisper(sor.18S, dm2$Sexe, type = c("centroid"))
rep.stage_18S.sor<- betadisper(sor.18S, dm2$Reproductive.Stage, type = c("centroid"))
size.class_18S.sor<- betadisper(sor.18S, dm2$SIZE_CLASS, type = c("centroid"))
Year_18S.sor<- betadisper(sor.18S, dm2$Year, type = c("centroid"))
anova(Weight_18S.sor)
```

```
## Analysis of Variance Table
##
## Response: Distances
##           Df  Sum Sq  Mean Sq F value  Pr(>F)
## Groups    36 1.64314 0.045643  15.714 0.00046 ***
## Residuals  7 0.02033 0.002905
## ---
## Signif. codes:  0 '***' 0.001 '**' 0.01 '*' 0.05 '.' 0.1 ' ' 1
```

```
plot (Weight_18S.sor)
```

```
## Warning in plot.xy(xy.coords(x, y), type = type, ...): valeur '26' de pch non
## implémentée
```

```
## Warning in plot.xy(xy.coords(x, y), type = type, ...): valeur '31' de pch non
## implémentée
```

```
## Warning in plot.xy(xy.coords(x, y), type = type, ...): valeur '30' de pch non
## implémentée
```

```
## Warning in plot.xy(xy.coords(x, y), type = type, ...): valeur '27' de pch non
## implémentée
```

```
## Warning in plot.xy(xy.coords(x, y), type = type, ...): valeur '29' de pch non
## implémentée
```

```
## Warning in plot.xy(xy.coords(x, y), type = type, ...): valeur '28' de pch non
## implémentée
```

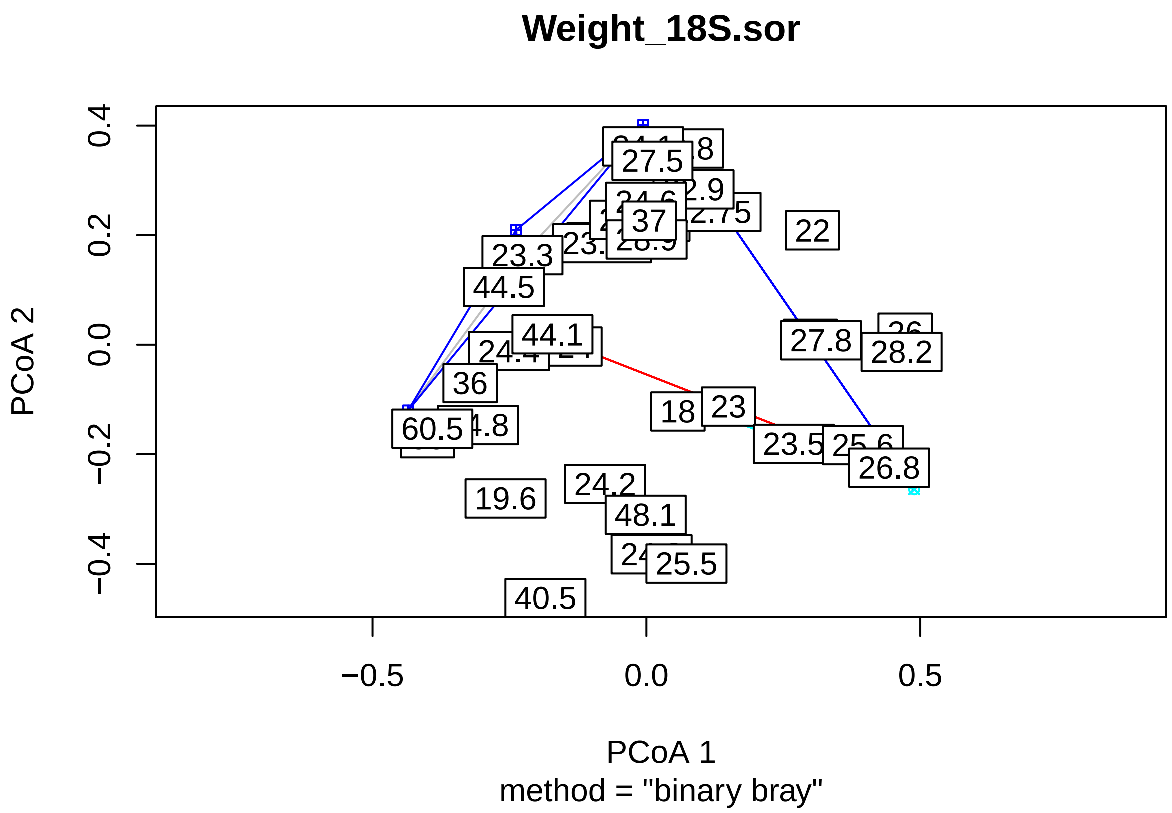


```r
anova(Port_18S.sor)
```

```
## Analysis of Variance Table
##
## Response: Distances
##           Df   Sum Sq   Mean Sq F value Pr(>F)
## Groups     3 0.029892 0.0099641  1.8234 0.1584
## Residuals 40 0.218589 0.0054647
```

```r
plot(Port_18S.sor)
```

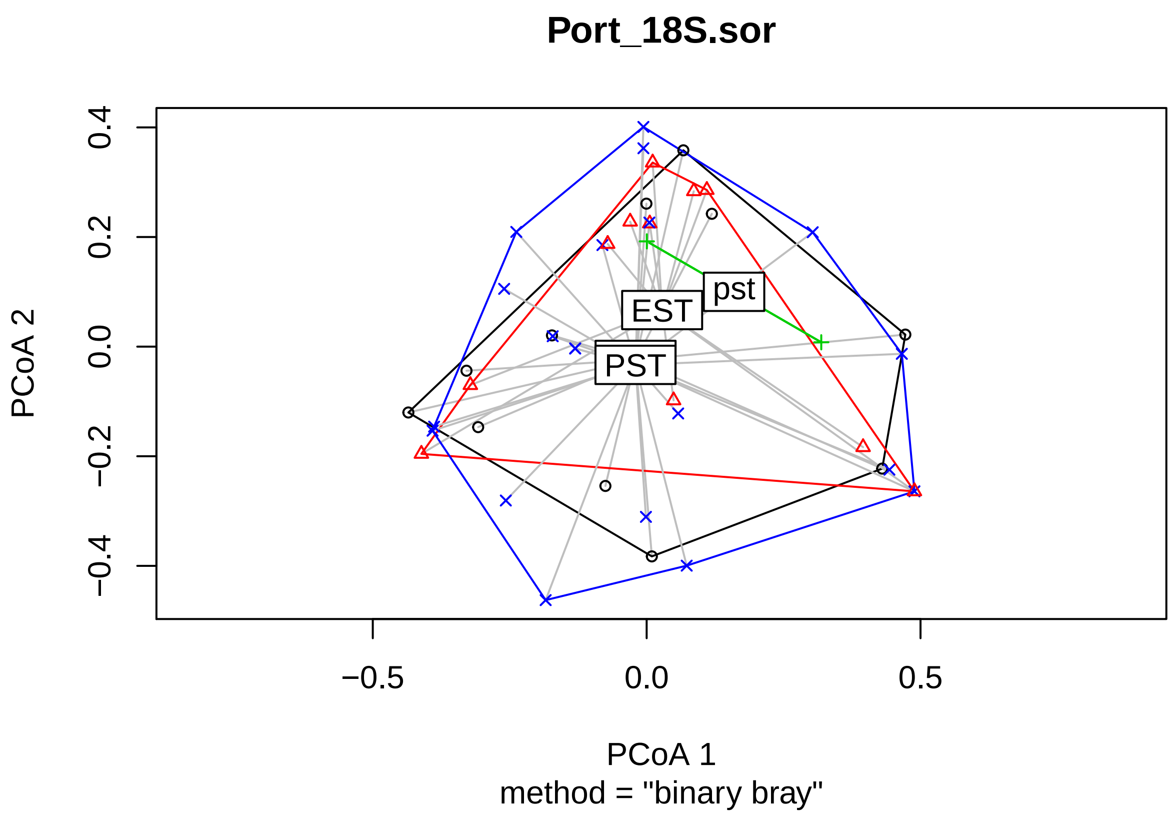


```
anova(mouth.Lengt_18S.sor)
```

```
## Analysis of Variance Table
##
## Response: Distances
##           Df  Sum Sq  Mean Sq F value    Pr(>F)
## Groups    29 2.86170 0.098679  74.153 4.178e-11 ***
## Residuals 14 0.01863 0.001331
## ---
## Signif. codes:  0 '***' 0.001 '**' 0.01 '*' 0.05 '.' 0.1 ' ' 1
```

```
plot(mouth.Lengt_18S.sor)
```

```
## Warning in plot.xy(xy.coords(x, y), type = type, ...): valeur '29' de pch non
## implémentée

## Warning in plot.xy(xy.coords(x, y), type = type, ...): valeur '26' de pch non
## implémentée

## Warning in plot.xy(xy.coords(x, y), type = type, ...): valeur '30' de pch non
## implémentée

## Warning in plot.xy(xy.coords(x, y), type = type, ...): valeur '26' de pch non
## implémentée
```

```
## Warning in plot.xy(xy.coords(x, y), type = type, ...): valeur '28' de pch non
## implémentée

## Warning in plot.xy(xy.coords(x, y), type = type, ...): valeur '27' de pch non
## implémentée
```

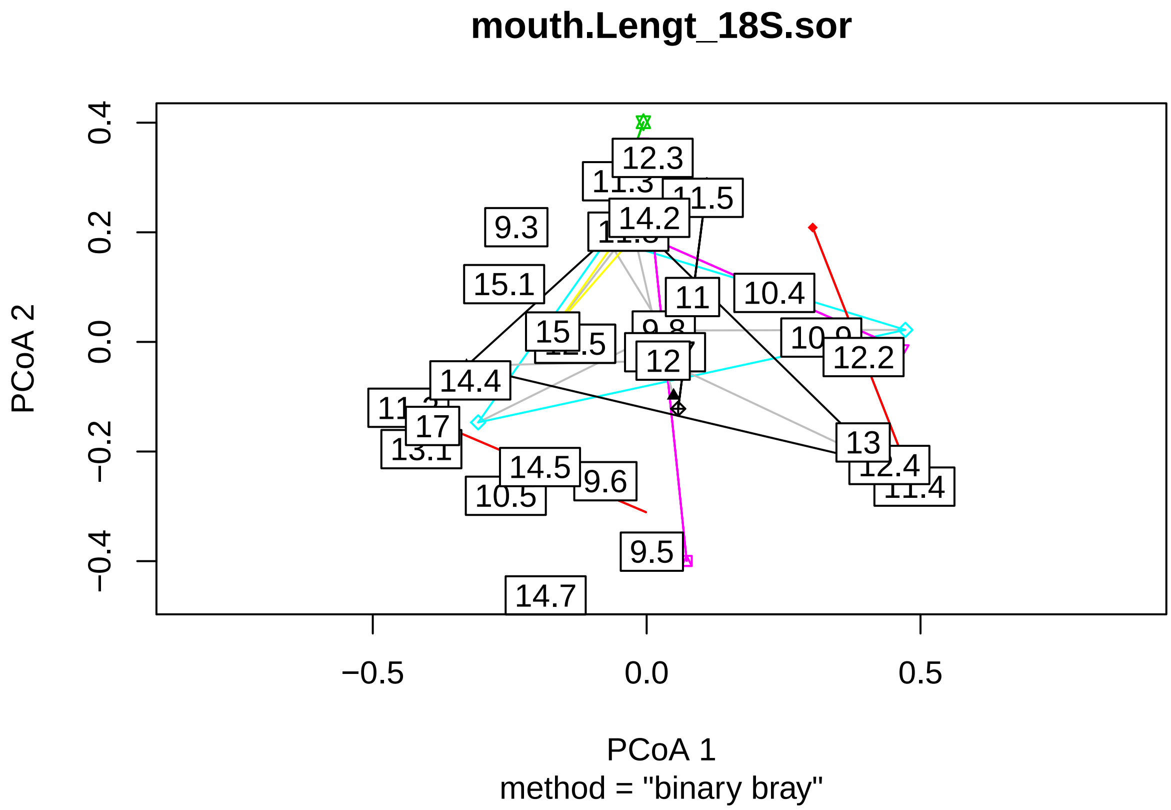


```r
anova(Sex_18S.sor)
```

```
## Analysis of Variance Table
##
## Response: Distances
##           Df   Sum Sq   Mean Sq F value  Pr(>F)
## Groups     2 0.038222 0.0191109  3.3269 0.04582 *
## Residuals 41 0.235520 0.0057444
## ---
## Signif. codes:  0 '***' 0.001 '**' 0.01 '*' 0.05 '.' 0.1 ' ' 1
```

```r
plot(Sex_18S.sor)
```

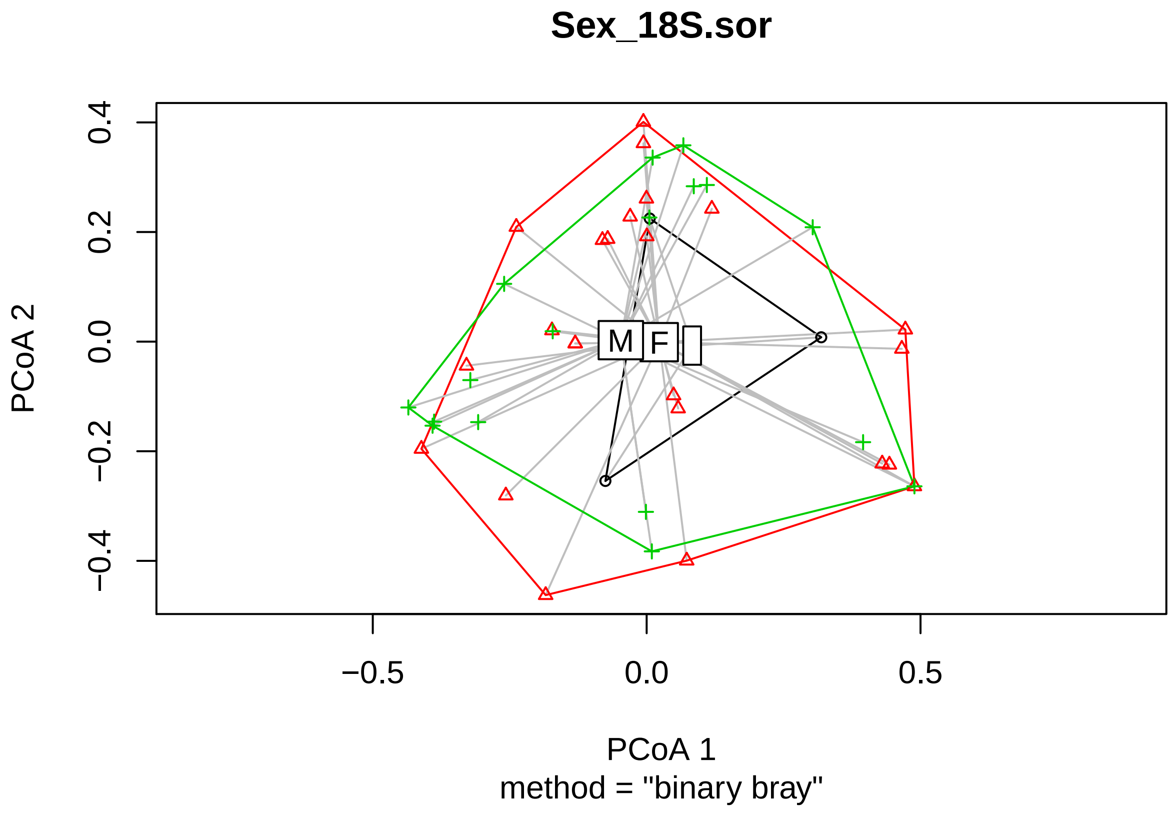


```r
anova(rep.stage_18S.sor)
```

```
## Analysis of Variance Table
##
## Response: Distances
##           Df   Sum Sq   Mean Sq F value Pr(>F)
## Groups     3 0.058879 0.0196264  3.6315 0.0208 *
## Residuals 40 0.216182 0.0054046
## ---
## Signif. codes:  0 '***' 0.001 '**' 0.01 '*' 0.05 '.' 0.1 ' ' 1
```

```r
plot(rep.stage_18S.sor)
```

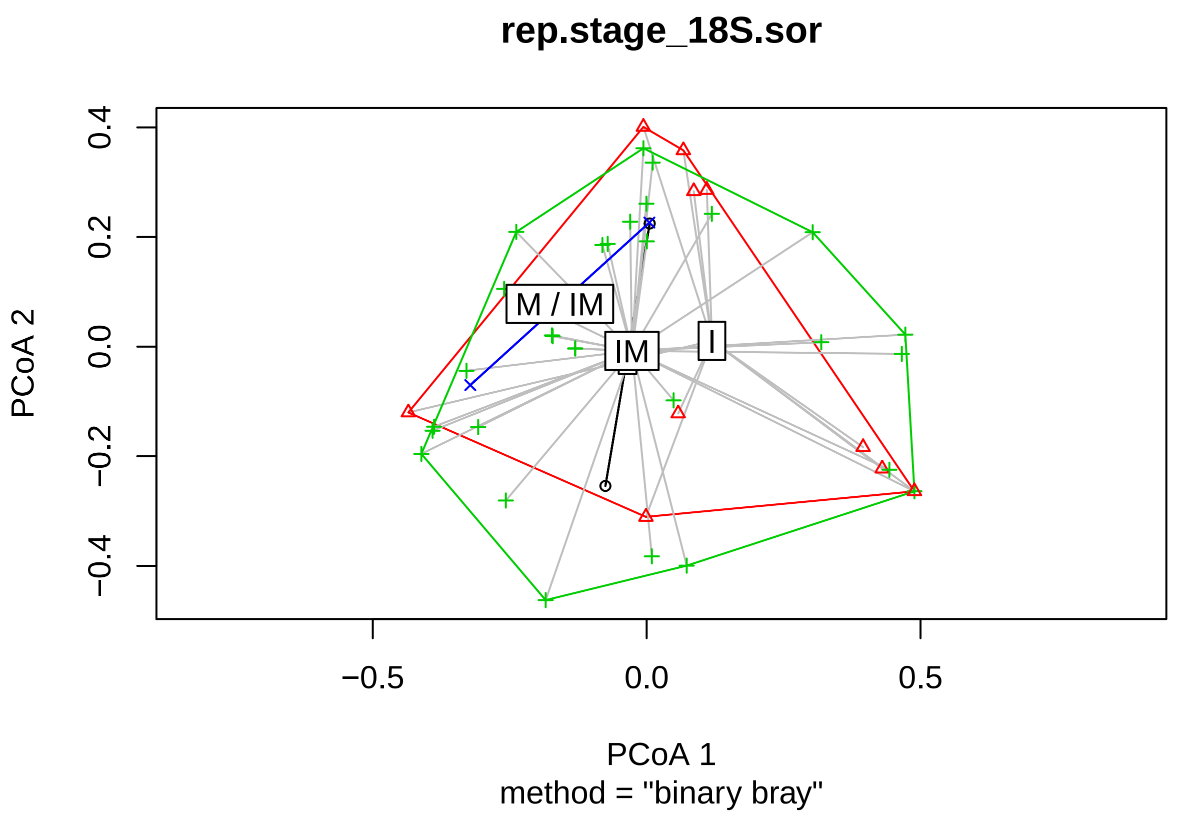


```
anova(size.class_18S.sor)
```

```
## Analysis of Variance Table
##
## Response: Distances
##           Df   Sum Sq  Mean Sq F value  Pr(>F)
## Groups     1 0.038615 0.038615  6.3065 0.01596 *
## Residuals 42 0.257169 0.006123
## ---
## Signif. codes:  0 '***' 0.001 '**' 0.01 '*' 0.05 '.' 0.1 ' ' 1
```

```
plot(size.class_18S.sor)
```

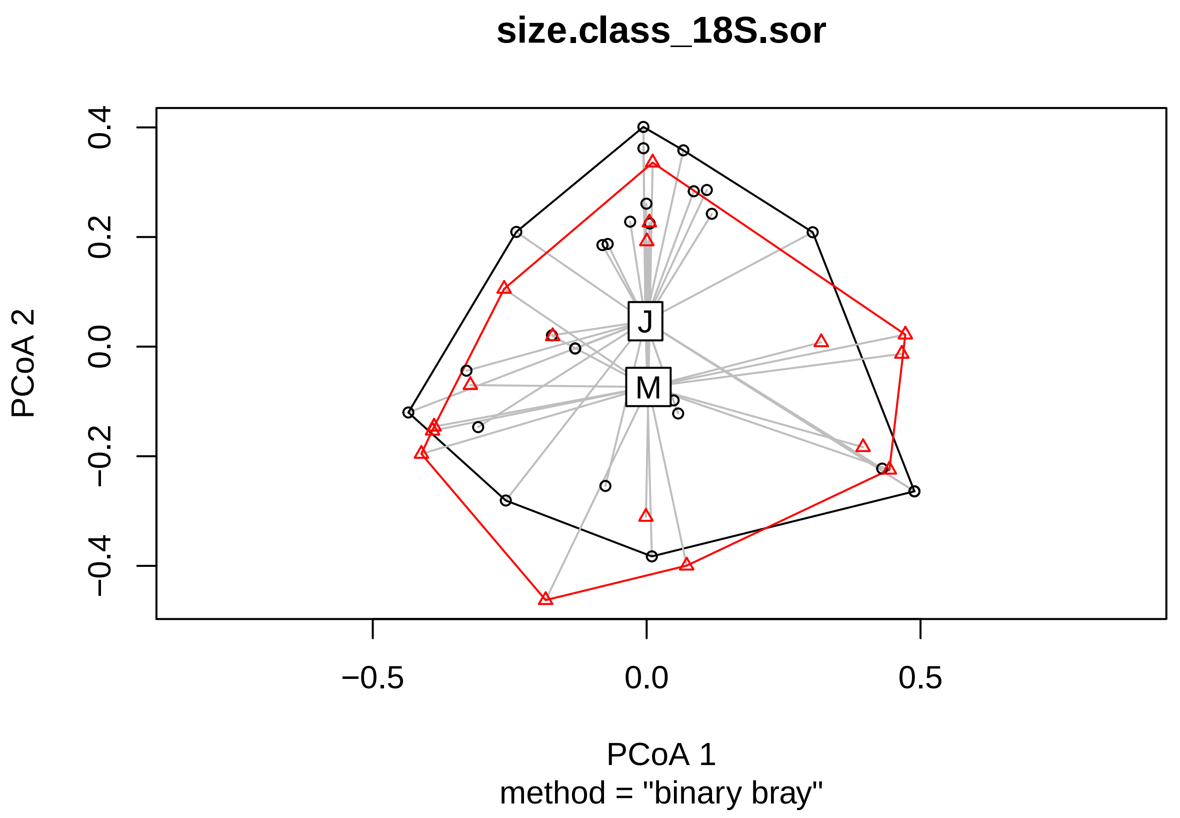


```r
anova(Year_18S.sor)
```

```
## Analysis of Variance Table
##
## Response: Distances
##           Df   Sum Sq   Mean Sq F value Pr(>F)
## Groups     3 0.023615 0.0078718  1.1881 0.3265
## Residuals 40 0.265013 0.0066253
```

```r
plot(Year_18S.sor)
```

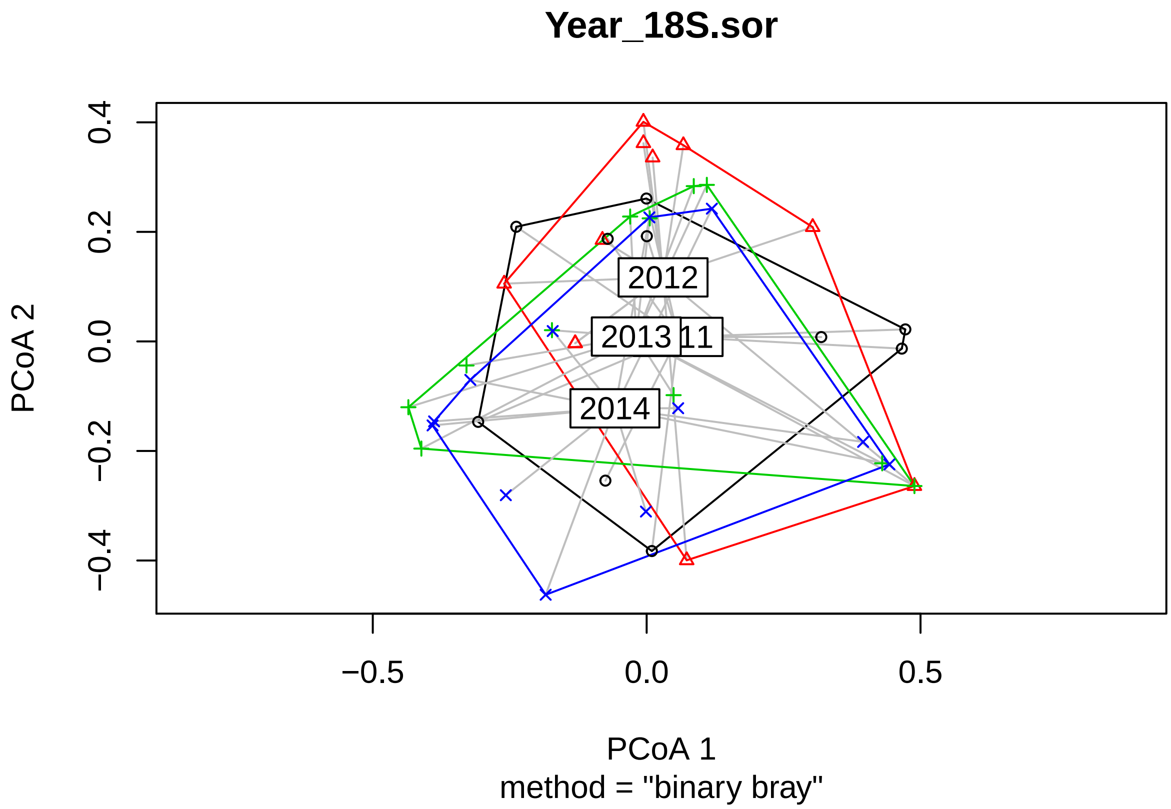


```
#####Euclidian COI
euc.COI =vegdist(df4_8,method="euclidean")
anosim(euc.COI, dm$Weight)
```

```
##
## Call:
## anosim(x = euc.COI, grouping = dm$Weight)
## Dissimilarity: euclidean
##
## ANOSIM statistic R: 0.2279
##       Significance: 0.113
##
## Permutation: free
## Number of permutations: 999
```

```
anosim(euc.COI, dm$code.port)
```

```
##
## Call:
## anosim(x = euc.COI, grouping = dm$code.port)
## Dissimilarity: euclidean
##
## ANOSIM statistic R: 0.04341
##       Significance: 0.205
##
```

```
## Permutation: free
## Number of permutations: 999
```

```r
anosim(euc.COI, dm$Longueur_Bouche)
```

```
##
## Call:
## anosim(x = euc.COI, grouping = dm$Longueur_Bouche)
## Dissimilarity: euclidean
##
## ANOSIM statistic R: 0.2149
##       Significance: 0.05
##
## Permutation: free
## Number of permutations: 999
```

```r
anosim(euc.COI, dm$Sexe)
```

```
##
## Call:
## anosim(x = euc.COI, grouping = dm$Sexe)
## Dissimilarity: euclidean
##
## ANOSIM statistic R: 0.0436
##       Significance: 0.158
##
## Permutation: free
## Number of permutations: 999
```

```r
anosim(euc.COI, dm$Reproductive.Stage)
```

```
##
## Call:
## anosim(x = euc.COI, grouping = dm$Reproductive.Stage)
## Dissimilarity: euclidean
##
## ANOSIM statistic R: -0.07427
##       Significance: 0.81
##
## Permutation: free
## Number of permutations: 999
```

```r
anosim(euc.COI, dm$SIZE_CLASS)
```

```
##
## Call:
## anosim(x = euc.COI, grouping = dm$SIZE_CLASS)
## Dissimilarity: euclidean
##
## ANOSIM statistic R: -0.01529
##       Significance: 0.558
```

```
##
## Permutation: free
## Number of permutations: 999
```

```
anosim(euc.COI, dm$Year)
```

```
##
## Call:
## anosim(x = euc.COI, grouping = dm$Year)
## Dissimilarity: euclidean
##
## ANOSIM statistic R: 0.05183
##       Significance: 0.085
##
## Permutation: free
## Number of permutations: 999
```

```
Weight_COI.euc<- betadisper(euc.COI, dm$Weight, type = c("centroid"))
Port_COI.euc<- betadisper(euc.COI, dm$code.port, type = c("centroid"))
mouth.Length_COI.euc<- betadisper(euc.COI, dm$Longueur_Bouche, type = c("centroid"))
Sex_COI.euc<- betadisper(euc.COI, dm$Sexe, type = c("centroid"))
rep.stage_COI.euc<- betadisper(euc.COI, dm$Reproductive.Stage, type = c("centroid"))
size.class_COI.euc<- betadisper(euc.COI, dm$SIZE_CLASS, type = c("centroid"))
Year_COI.euc<- betadisper(euc.COI, dm$Year, type = c("centroid"))

anova(Weight_COI.euc)
```

```
## Analysis of Variance Table
##
## Response: Distances
##           Df  Sum Sq  Mean Sq F value   Pr(>F)
## Groups    37 1.41857 0.038340  10.764 0.001569 **
## Residuals  7 0.02493 0.003562
## ---
## Signif. codes:  0 '***' 0.001 '**' 0.01 '*' 0.05 '.' 0.1 ' ' 1
```

```
plot (Weight_COI.euc)
```

```
## Warning in plot.xy(xy.coords(x, y), type = type, ...): valeur '28' de pch non
## implémentée

## Warning in plot.xy(xy.coords(x, y), type = type, ...): valeur '30' de pch non
## implémentée

## Warning in plot.xy(xy.coords(x, y), type = type, ...): valeur '29' de pch non
## implémentée

## Warning in plot.xy(xy.coords(x, y), type = type, ...): valeur '27' de pch non
## implémentée

## Warning in plot.xy(xy.coords(x, y), type = type, ...): valeur '31' de pch non
## implémentée
```

```
## Warning in plot.xy(xy.coords(x, y), type = type, ...): valeur '26' de pch non
## implémentée
```

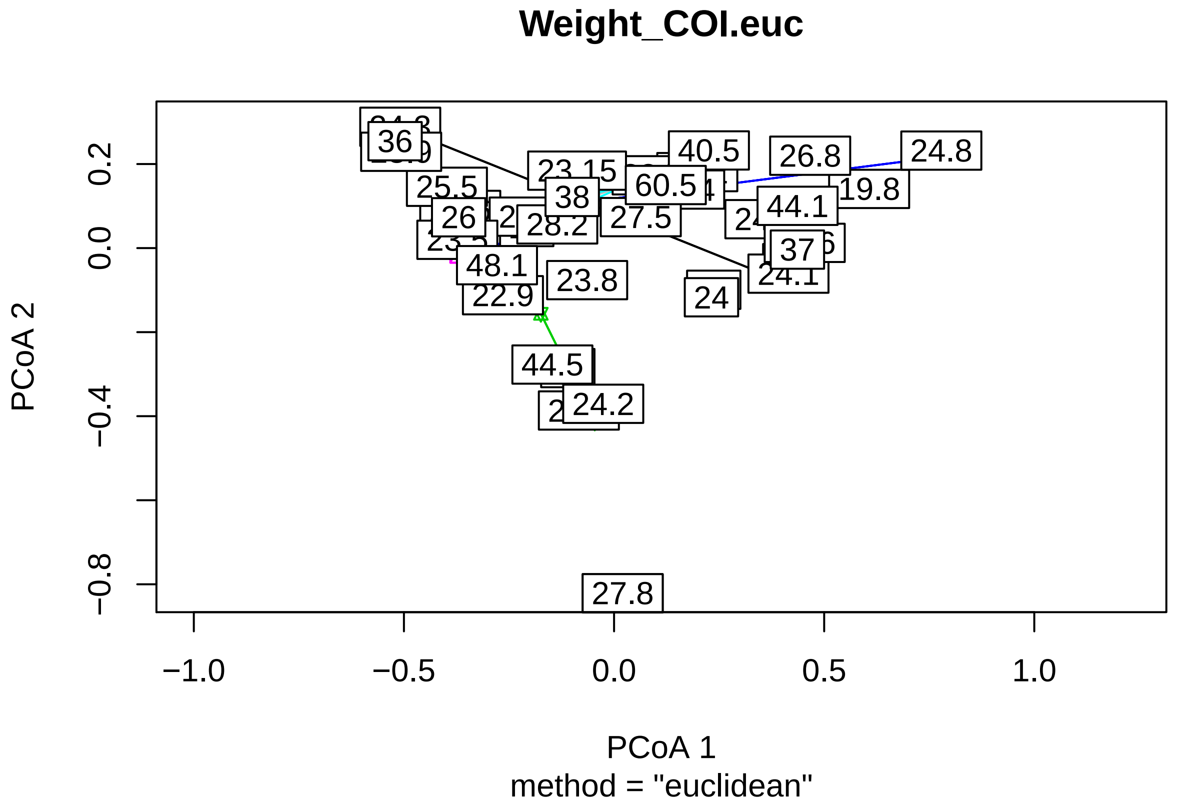


```r
anova(Port_COI.euc)
```

```
## Analysis of Variance Table
##
## Response: Distances
##           Df  Sum Sq  Mean Sq F value Pr(>F)
## Groups     3 0.10027 0.033424  1.0942 0.3624
## Residuals 41 1.25246 0.030548
```

```r
plot(Port_COI.euc)
```

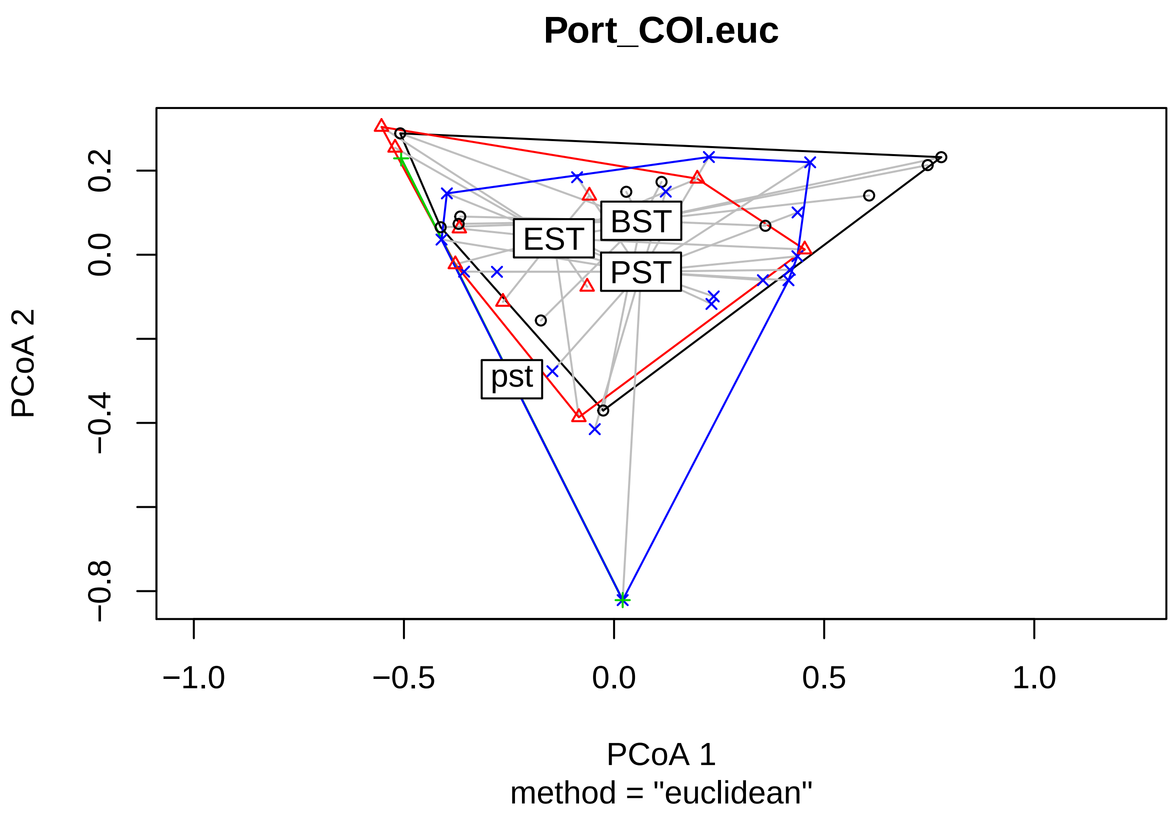


```r
anova(mouth.Length_COI.euc)
```

```
## Analysis of Variance Table
##
## Response: Distances
##           Df  Sum Sq  Mean Sq F value    Pr(>F)
## Groups    29 1.70356 0.058743  19.258 1.559e-07 ***
## Residuals 15 0.04575 0.003050
## ---
## Signif. codes:  0 '***' 0.001 '**' 0.01 '*' 0.05 '.' 0.1 ' ' 1
```

```r
plot(mouth.Length_COI.euc)
```

```
## Warning in plot.xy(xy.coords(x, y), type = type, ...): valeur '29' de pch non
## implémentée

## Warning in plot.xy(xy.coords(x, y), type = type, ...): valeur '26' de pch non
## implémentée

## Warning in plot.xy(xy.coords(x, y), type = type, ...): valeur '30' de pch non
## implémentée

## Warning in plot.xy(xy.coords(x, y), type = type, ...): valeur '26' de pch non
## implémentée
```

```
## Warning in plot.xy(xy.coords(x, y), type = type, ...): valeur '28' de pch non
## implémentée

## Warning in plot.xy(xy.coords(x, y), type = type, ...): valeur '27' de pch non
## implémentée
```

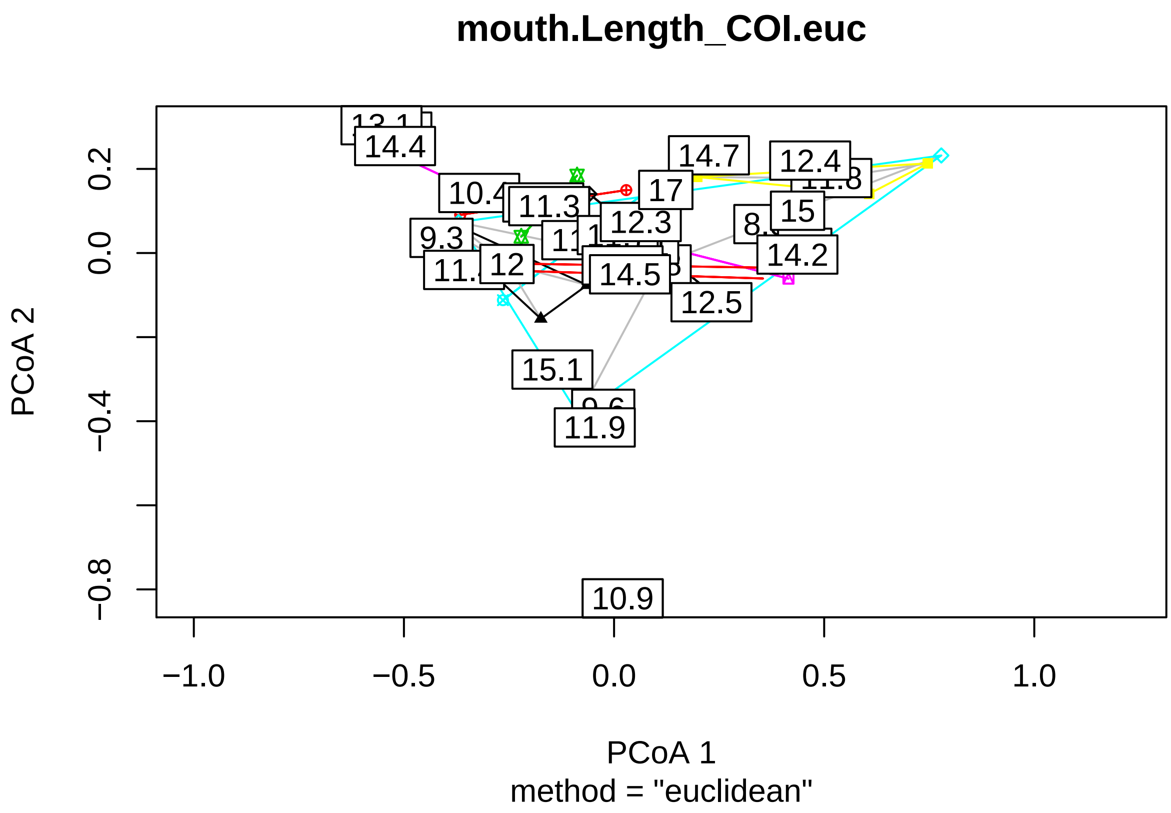


```r
anova(Sex_COI.euc)
```

```
## Analysis of Variance Table
##
## Response: Distances
##           Df  Sum Sq  Mean Sq F value Pr(>F)
## Groups     2 0.05264 0.026318  0.7447 0.4811
## Residuals 42 1.48437 0.035342
```

```r
plot(Sex_COI.euc)
```

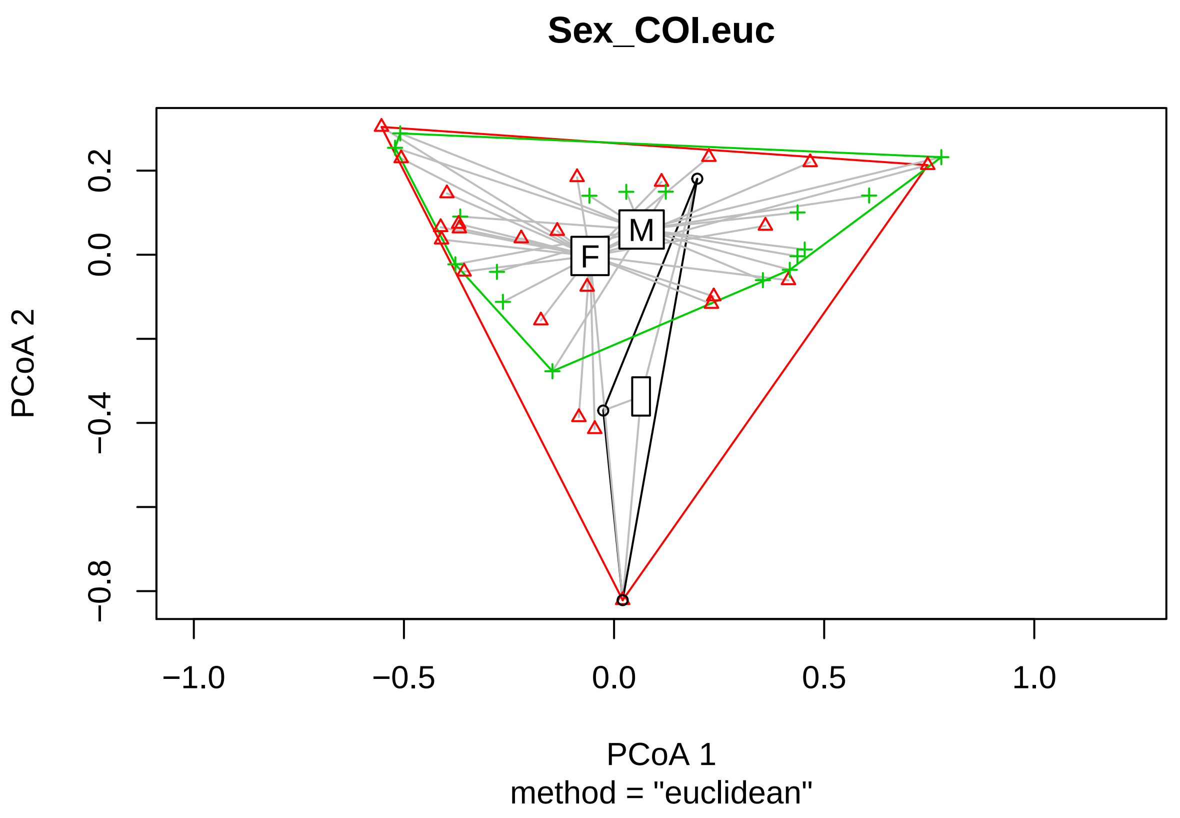


```r
anova(rep.stage_COI.euc)
```

```
## Analysis of Variance Table
##
## Response: Distances
##           Df  Sum Sq  Mean Sq F value Pr(>F)
## Groups     3 0.08514 0.028381  0.7531 0.5269
## Residuals 41 1.54500 0.037683
```

```r
plot(rep.stage_COI.euc)
```

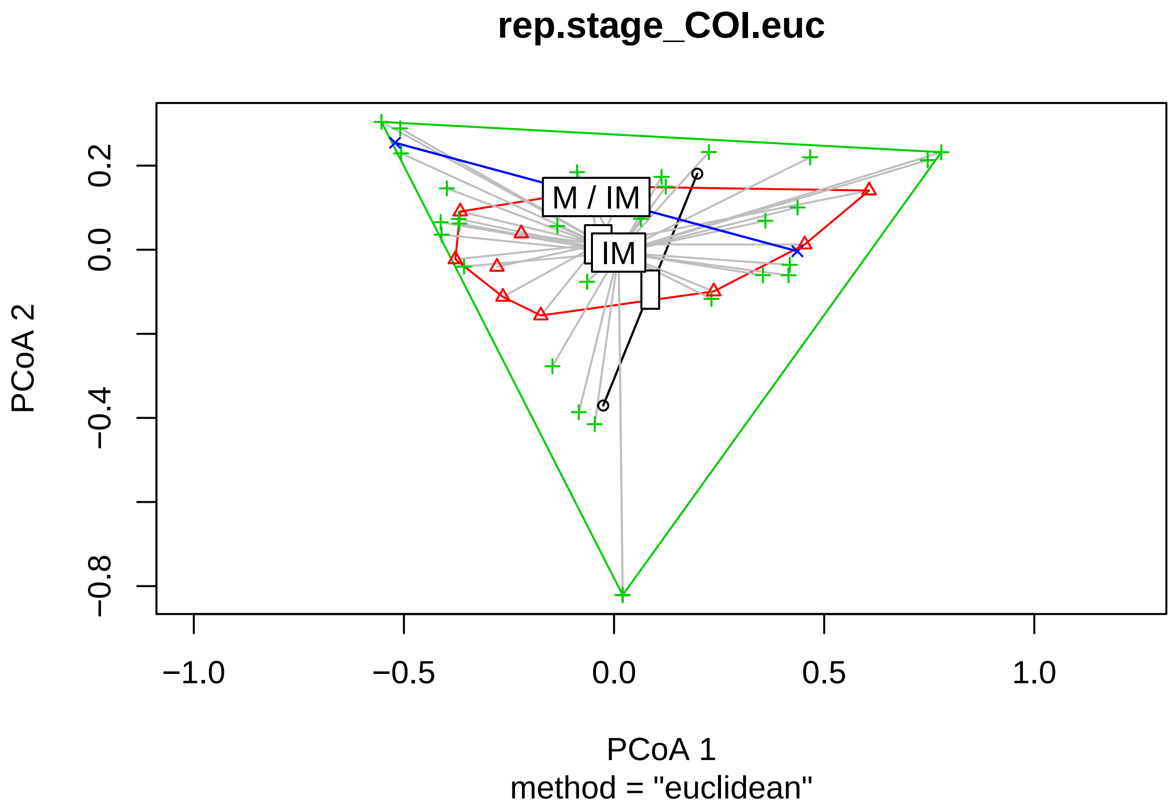


```r
anova(size.class_COI.euc)
```

```
## Analysis of Variance Table
##
## Response: Distances
##           Df  Sum Sq  Mean Sq F value Pr(>F)
## Groups     1 0.00114 0.001141  0.0287 0.8662
## Residuals 43 1.70816 0.039725
```

```r
plot(size.class_COI.euc)
```

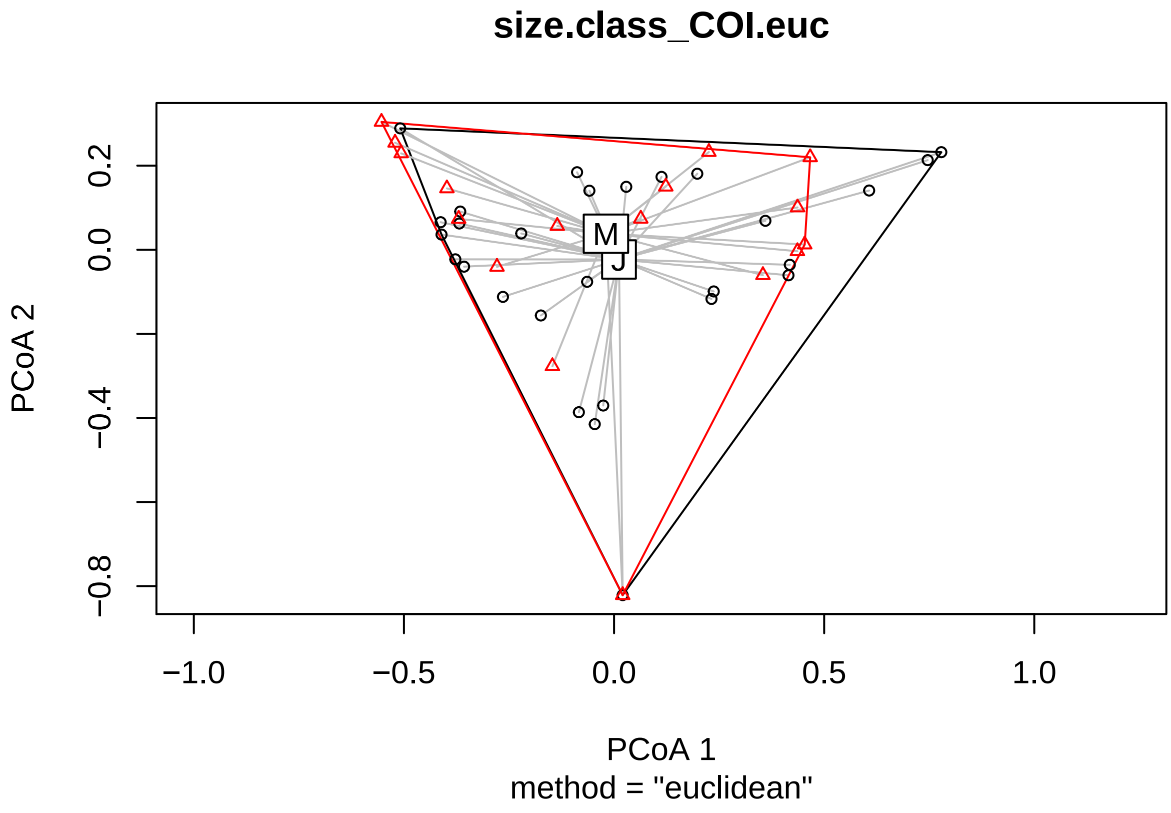


```
anova(Year_COI.euc)
```

```
## Analysis of Variance Table
##
## Response: Distances
##           Df  Sum Sq  Mean Sq F value Pr(>F)
## Groups     3 0.15961 0.053204   1.283  0.293
## Residuals 41 1.70026 0.041470
```

```
plot(Year_COI.euc)
```

Year_COI.euc

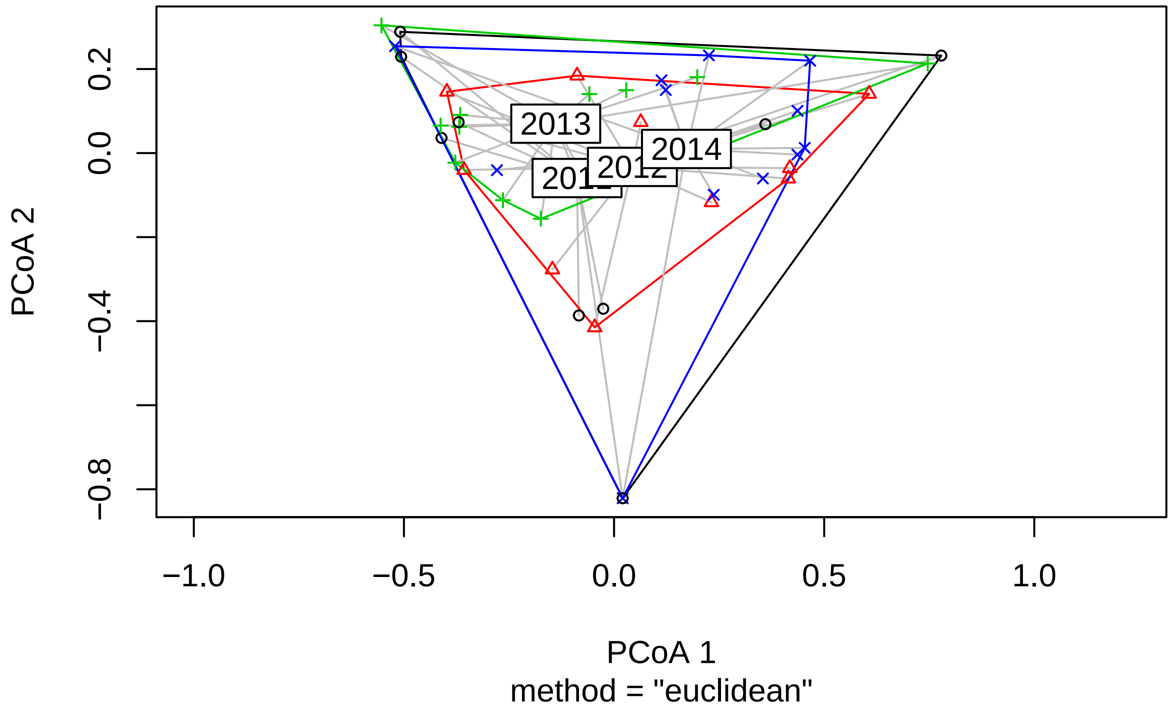


```r
#####sorensen COI
sor.COI=vegdist(df4_8,binary=TRUE)
anosim(sor.COI, dm$Weight)
```

```
##
## Call:
## anosim(x = sor.COI, grouping = dm$Weight)
## Dissimilarity: binary bray
##
## ANOSIM statistic R: 0.08936
##       Significance: 0.318
##
## Permutation: free
## Number of permutations: 999
```

```r
anosim(sor.COI, dm$code.port)
```

```
##
## Call:
## anosim(x = sor.COI, grouping = dm$code.port)
## Dissimilarity: binary bray
##
## ANOSIM statistic R: 0.0196
##       Significance: 0.335
##
```

```
## Permutation: free
## Number of permutations: 999
```

```r
anosim(sor.COI, dm$Longueur_Bouche)
```

```
##
## Call:
## anosim(x = sor.COI, grouping = dm$Longueur_Bouche)
## Dissimilarity: binary bray
##
## ANOSIM statistic R: -0.1258
##       Significance: 0.834
##
## Permutation: free
## Number of permutations: 999
```

```r
anosim(sor.COI, dm$Sexe)
```

```
##
## Call:
## anosim(x = sor.COI, grouping = dm$Sexe)
## Dissimilarity: binary bray
##
## ANOSIM statistic R: 0.02986
##       Significance: 0.253
##
## Permutation: free
## Number of permutations: 999
```

```r
anosim(sor.COI, dm$Reproductive.Stage)
```

```
##
## Call:
## anosim(x = sor.COI, grouping = dm$Reproductive.Stage)
## Dissimilarity: binary bray
##
## ANOSIM statistic R: 0.01397
##       Significance: 0.417
##
## Permutation: free
## Number of permutations: 999
```

```r
anosim(sor.COI, dm$SIZE_CLASS)
```

```
##
## Call:
## anosim(x = sor.COI, grouping = dm$SIZE_CLASS)
## Dissimilarity: binary bray
##
## ANOSIM statistic R: -0.07794
##       Significance: 0.958
```

```
##
## Permutation: free
## Number of permutations: 999
```

```
anosim(sor.COI, dm$Year)
```

```
##
## Call:
## anosim(x = sor.COI, grouping = dm$Year)
## Dissimilarity: binary bray
##
## ANOSIM statistic R: -0.00137
##       Significance: 0.463
##
## Permutation: free
## Number of permutations: 999
```

```
Weight_COI.sor<- betadisper(sor.COI, dm$Weight, type = c("centroid"))
Port_COI.sor<- betadisper(sor.COI, dm$code.port, type = c("centroid"))
mouth.Length_COI.sor<- betadisper(sor.COI, dm$Longueur_Bouche, type = c("centroid"))
Sex_COI.sor<- betadisper(sor.COI, dm$Sexe, type = c("centroid"))
rep.stage_COI.sor<- betadisper(sor.COI, dm$Reproductive.Stage, type = c("centroid"))
size.class_COI.sor<- betadisper(sor.COI, dm$SIZE_CLASS, type = c("centroid"))
Year_COI.sor<- betadisper(sor.COI, dm$Year, type = c("centroid"))

anova(Weight_COI.sor)
```

```
## Analysis of Variance Table
##
## Response: Distances
##           Df Sum Sq  Mean Sq F value    Pr(>F)
## Groups    37 1.2730 0.034405  13.603 0.0007349 ***
## Residuals  7 0.0177 0.002529
## ---
## Signif. codes:  0 '***' 0.001 '**' 0.01 '*' 0.05 '.' 0.1 ' ' 1
```

```
plot (Weight_COI.sor)
```

```
## Warning in plot.xy(xy.coords(x, y), type = type, ...): valeur '28' de pch non
## implémentée

## Warning in plot.xy(xy.coords(x, y), type = type, ...): valeur '30' de pch non
## implémentée

## Warning in plot.xy(xy.coords(x, y), type = type, ...): valeur '29' de pch non
## implémentée

## Warning in plot.xy(xy.coords(x, y), type = type, ...): valeur '27' de pch non
## implémentée

## Warning in plot.xy(xy.coords(x, y), type = type, ...): valeur '31' de pch non
## implémentée
```

```
## Warning in plot.xy(xy.coords(x, y), type = type, ...): valeur '26' de pch non
## implémentée
```

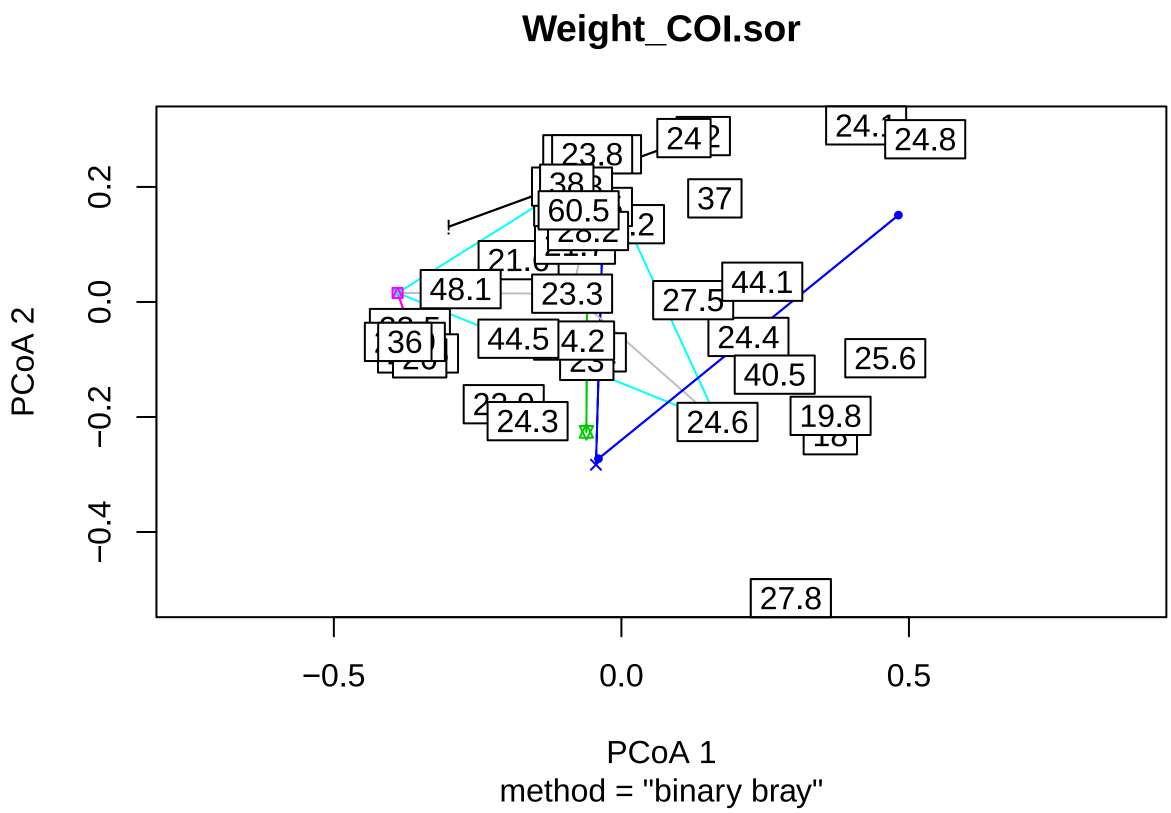


```r
anova(Port_COI.sor)
```

```
## Analysis of Variance Table
##
## Response: Distances
##           Df  Sum Sq  Mean Sq F value Pr(>F)
## Groups     3 0.09739 0.032464  1.5571 0.2144
## Residuals 41 0.85483 0.020849
```

```r
plot(Port_COI.sor)
```

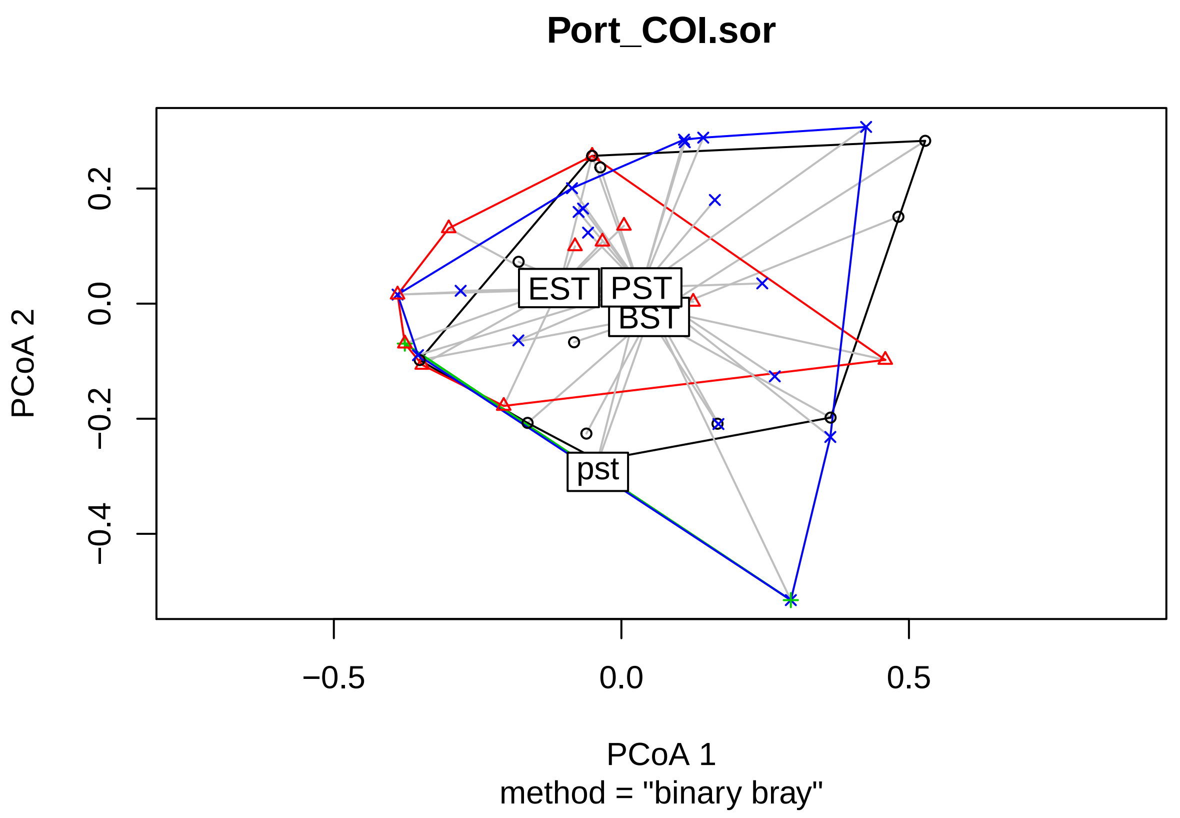


```r
anova(mouth.Length_COI.sor)
```

```
## Analysis of Variance Table
##
## Response: Distances
##           Df  Sum Sq  Mean Sq F value    Pr(>F)
## Groups    29 1.99564 0.068815  33.653 2.946e-09 ***
## Residuals 15 0.03067 0.002045
## ---
## Signif. codes:  0 '***' 0.001 '**' 0.01 '*' 0.05 '.' 0.1 ' ' 1
```

```r
plot(mouth.Length_COI.sor)
```

```
## Warning in plot.xy(xy.coords(x, y), type = type, ...): valeur '29' de pch non
## implémentée

## Warning in plot.xy(xy.coords(x, y), type = type, ...): valeur '26' de pch non
## implémentée

## Warning in plot.xy(xy.coords(x, y), type = type, ...): valeur '30' de pch non
## implémentée

## Warning in plot.xy(xy.coords(x, y), type = type, ...): valeur '26' de pch non
## implémentée
```

```
## Warning in plot.xy(xy.coords(x, y), type = type, ...): valeur '28' de pch non
## implémentée

## Warning in plot.xy(xy.coords(x, y), type = type, ...): valeur '27' de pch non
## implémentée
```

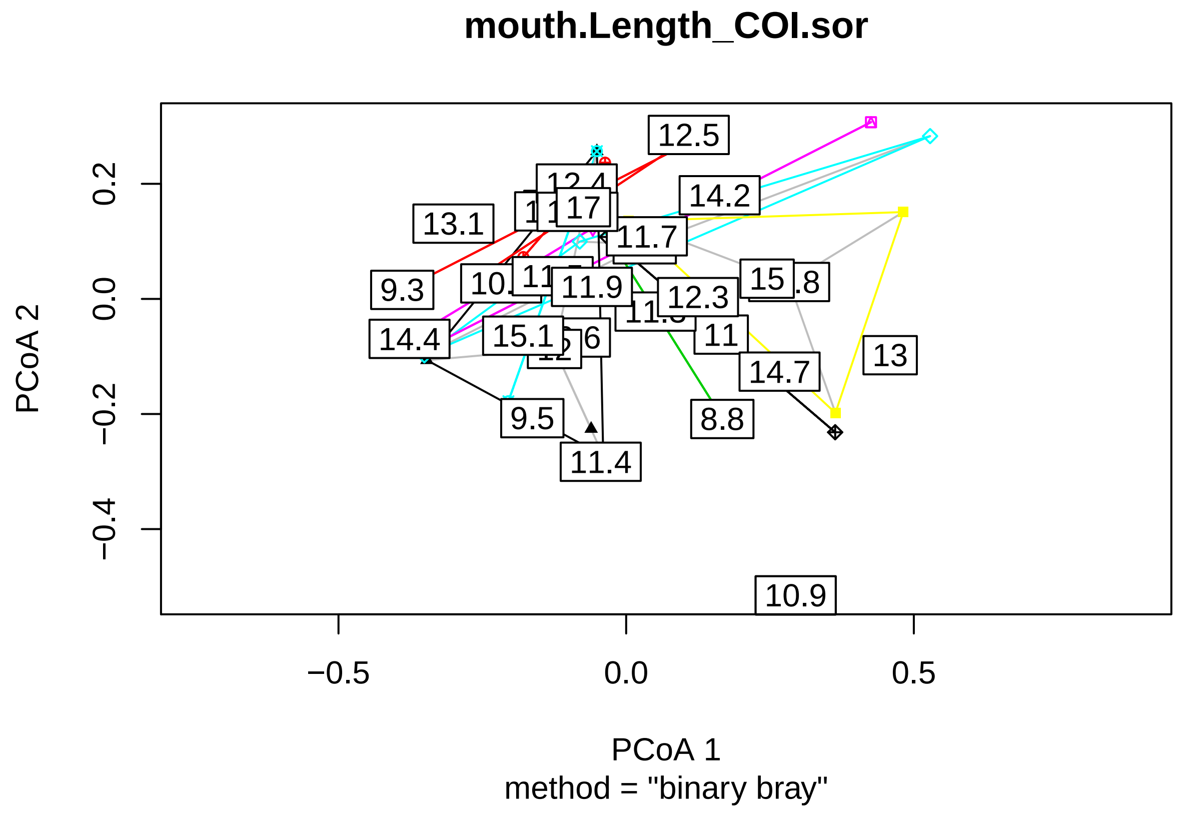


```r
anova(Sex_COI.sor)
```

```
## Analysis of Variance Table
##
## Response: Distances
##           Df  Sum Sq   Mean Sq F value Pr(>F)
## Groups     2 0.01099 0.0054928  0.2413 0.7867
## Residuals 42 0.95619 0.0227665
```

```r
plot(Sex_COI.sor)
```

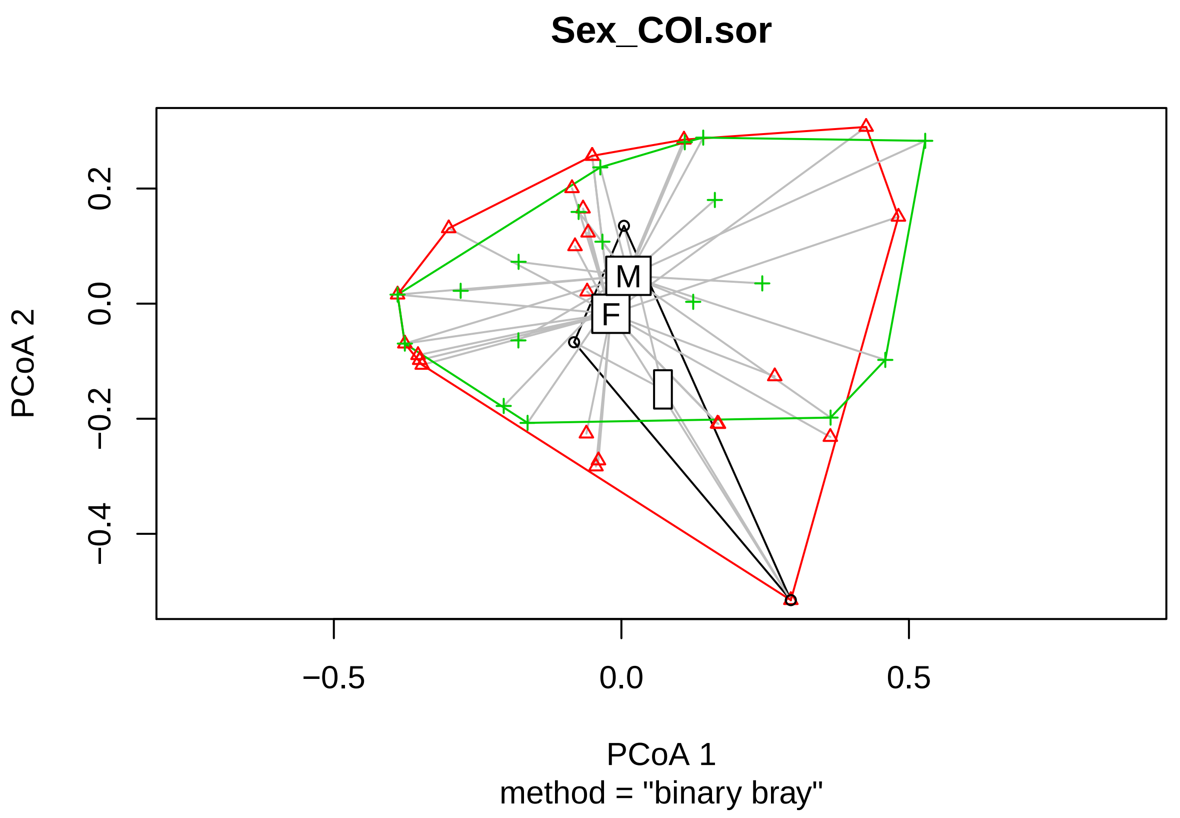


```
anova(rep.stage_COI.sor)
```

```
## Analysis of Variance Table
##
## Response: Distances
##           Df  Sum Sq  Mean Sq F value Pr(>F)
## Groups     3 0.05859 0.019531  0.7842 0.5097
## Residuals 41 1.02120 0.024907
```

```
plot(rep.stage_COI.sor)
```

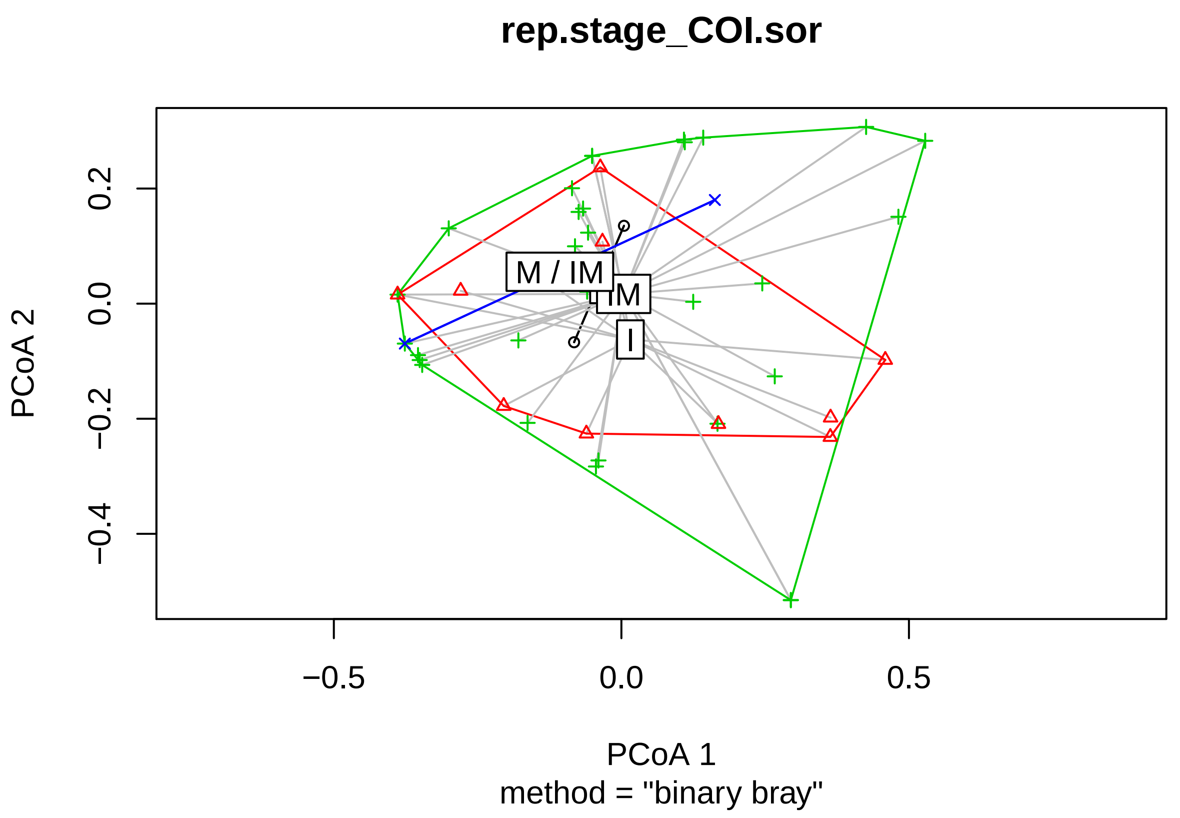


```r
anova(size.class_COI.sor)
```

```
## Analysis of Variance Table
##
## Response: Distances
##           Df  Sum Sq  Mean Sq F value Pr(>F)
## Groups     1 0.07354 0.073541   2.807 0.1011
## Residuals 43 1.12656 0.026199
```

```r
plot(size.class_COI.sor)
```

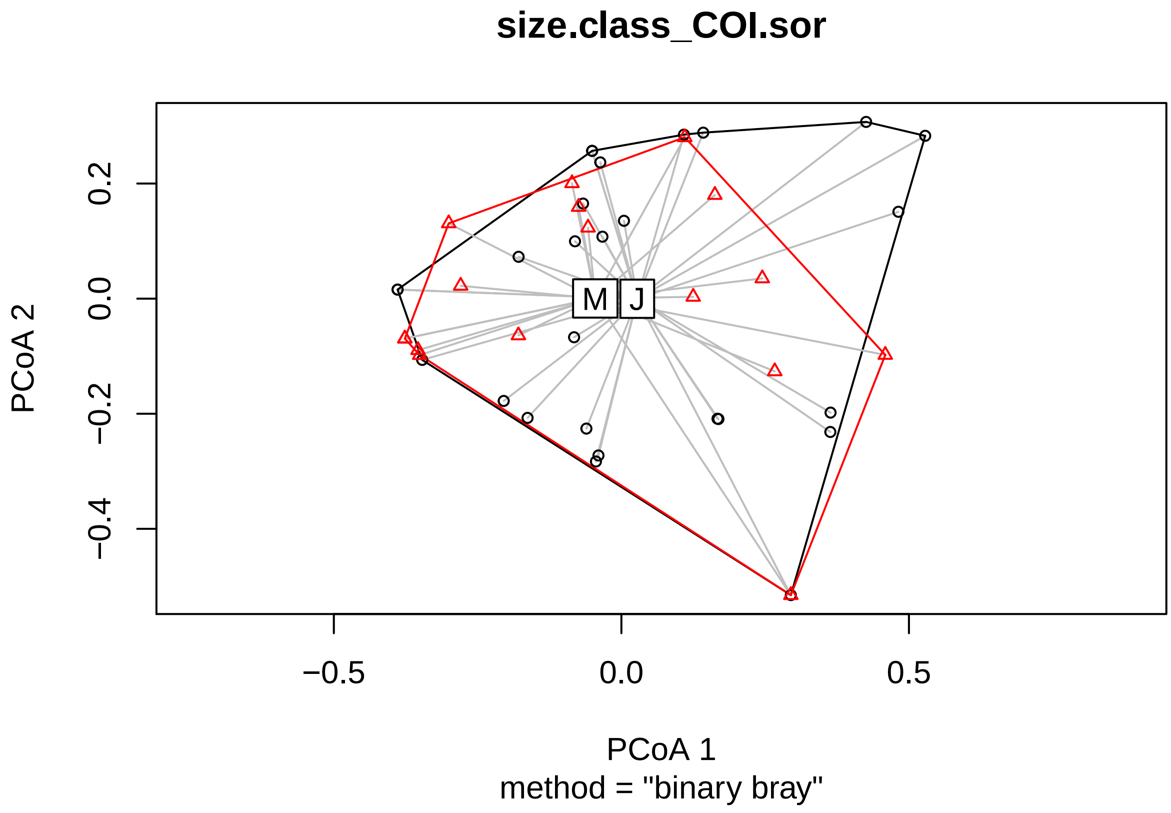


```r
anova(Year_COI.sor)
```

```
## Analysis of Variance Table
##
## Response: Distances
##           Df  Sum Sq  Mean Sq F value Pr(>F)
## Groups     3 0.05604 0.018681  0.8481 0.4757
## Residuals 41 0.90314 0.022028
```

```r
plot(Year_COI.sor)
```

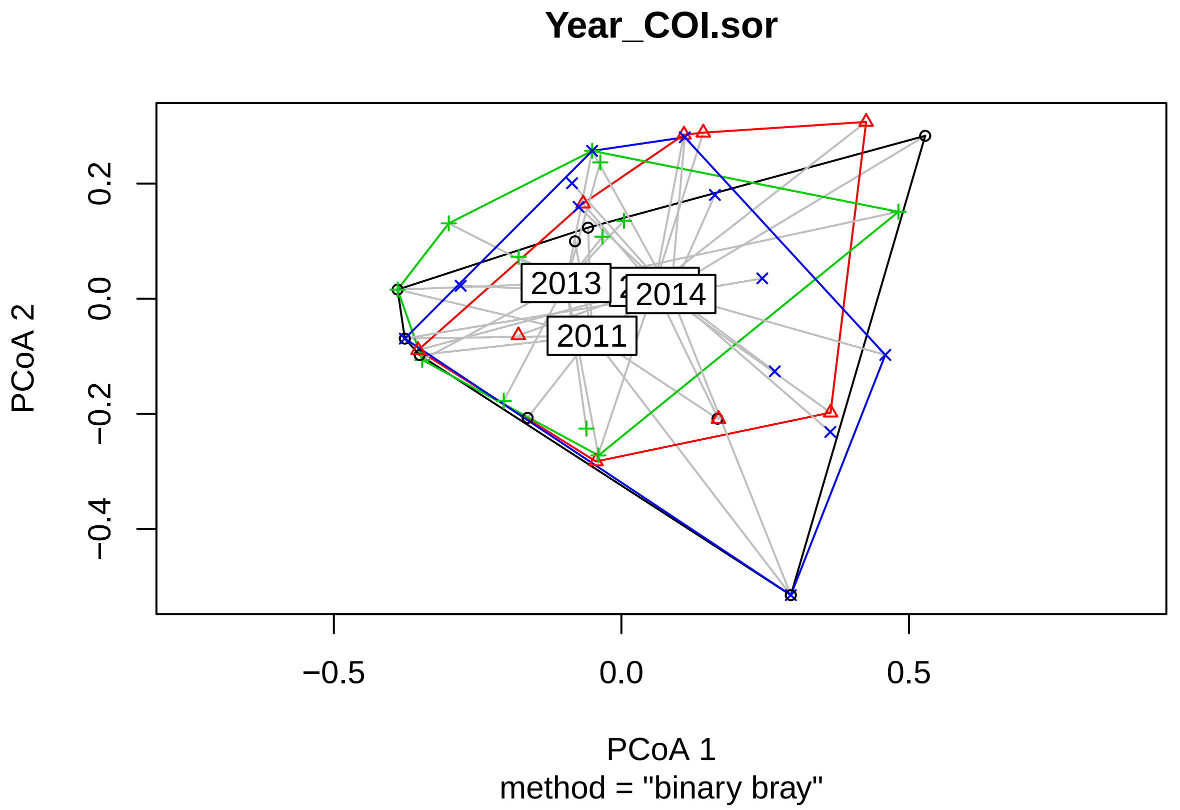

6.GlM and predictive models and their Drawings

```
#PREP DATASETS
tPA_Tuna18S_D<-t(PA_Tuna18S_D)
tPA_TunaCOI_D<-t(PA_TunaCOI_D)
df5 <- cbind(dm2,tPA_Tuna18S_D)
df6 <- cbind(dm,tPA_TunaCOI_D)
PA_TunaCOI_D
```

```
##                  M275 J290 J269 M281 M261 M198 M273 J233 J201 M280 J222 M118
## anchovy             0    1    1    1    1    1    1    1    0    1    1    0
## Arthropoda          0    0    0    1    1    1    0    0    1    1    0    1
## cephaloda           0    0    0    0    0    0    0    0    0    0    0    0
## Cepolaepola         0    0    0    0    0    0    0    0    0    0    0    0
## Chaetognatha        0    0    0    0    0    0    0    0    0    0    0    0
## Cnidaria            0    0    0    0    0    1    0    0    0    1    0    0
## Echinodermata       0    0    0    0    0    0    0    0    0    0    0    0
## mackerel            0    0    1    0    0    0    0    0    0    0    0    1
## Mollusca            0    0    1    0    0    0    0    0    0    0    0    0
## sardine             1    0    0    0    0    1    1    1    1    1    1    1
## sprat               0    0    1    1    0    0    0    1    1    0    1    0
## Tunicata            0    0    0    0    0    0    0    0    0    0    0    0
## Vertebrata          0    0    1    1    0    0    0    0    1    0    0    1
## Xenacoelomorpha     0    0    0    0    0    0    0    0    0    0    0    0
##                  M277 J221 M262 M279 J226 J53 J215 M117 M33 J47 J276 J52 M29
## anchovy             1    1    1    1    1   1    1    1   0   1    1   1   1
## Arthropoda          1    0    1    0    0   0    1    0   0   0    0   0   0
```

```
## cephaloda          0    0    0    0    0   0    0    0   0   0    0   0   0
## Cepolaepola        0    0    0    0    0   0    0    0   0   0    0   0   0
## Chaetognatha       0    0    0    0    0   0    0    0   0   0    0   0   0
## Cnidaria           0    0    0    0    0   0    0    0   0   0    0   0   0
## Echinodermata      0    0    0    0    0   0    0    0   0   0    0   0   0
## mackerel           0    0    0    0    0   1    0    0   0   1    0   0   0
## Mollusca           0    0    0    0    0   0    0    0   0   1    0   0   0
## sardine            0    0    0    1    0   0    0    1   1   0    1   1   1
## sprat              0    1    0    0    0   0    0    1   0   0    1   1   1
## Tunicata           0    0    0    0    0   0    0    0   0   0    0   0   0
## Vertebrata         0    0    1    1    0   0    0    0   1   0    0   0   0
## Xenacoelomorpha    0    0    0    0    0   0    0    0   0   0    0   0   0
##                 J138 J125 J121 M49 J144 J134 J150 J227 J57 M30 J46 J145 J96
## anchovy            0    0    0   1    1    1    1    1   1   1   1    1   1
## Arthropoda         1    0    0   0    0    0    0    0   0   0   0    0   0
## cephaloda          1    0    0   0    0    0    0    0   0   0   0    0   0
## Cepolaepola        0    1    1   0    0    0    0    0   0   0   0    0   0
## Chaetognatha       0    0    0   0    0    0    0    0   0   0   0    0   0
## Cnidaria           0    0    0   0    0    0    0    0   0   0   0    0   0
## Echinodermata      0    0    0   0    0    0    0    0   0   0   0    0   0
## mackerel           1    0    0   1    1    1    1    1   1   0   1    0   1
## Mollusca           0    0    0   0    0    0    0    0   0   0   0    0   0
## sardine            1    0    0   0    1    0    1    0   0   0   0    1   0
## sprat              0    0    0   0    1    0    1    1   0   1   1    0   0
## Tunicata           0    0    0   0    0    0    0    0   0   0   0    0   0
## Vertebrata         0    0    0   0    0    0    0    0   1   0   0    0   0
## Xenacoelomorpha    0    0    0   0    0    0    0    0   0   0   0    0   0
##                 J164 M123 M253 J229 J217 J205 J170
## anchovy            1    1    0    1    1    1    1
## Arthropoda         0    0    1    0    1    0    0
## cephaloda          0    0    0    0    0    0    0
## Cepolaepola        0    0    0    0    0    0    0
## Chaetognatha       0    0    0    0    0    0    0
## Cnidaria           0    0    0    0    0    0    0
## Echinodermata      0    0    0    0    0    0    0
## mackerel           0    1    0    0    0    0    0
## Mollusca           0    0    0    0    0    0    0
## sardine            1    1    1    1    1    1    0
## sprat              0    1    1    0    1    1    1
## Tunicata           0    0    0    0    0    0    0
## Vertebrata         0    0    0    0    0    0    0
## Xenacoelomorpha    0    0    0    0    0    0    0
```

```
PA_Tuna18S_D
```

```
##                 M275 J290 J269 M281 M261 M198 M273 J233 J201 M280 J222 M118
## Annelida           0    0    0    0    0    0    0    0    0    0    0    0
## Arthropoda         0    0    0    0    1    1    1    0    1    0    0    1
## Cnidaria           0    0    0    1    1    1    1    0    0    1    0    1
## Ctenophora         0    0    0    0    0    0    0    0    0    0    0    1
## Platyhelminthes    0    0    0    0    0    0    0    0    0    0    0    0
## Tunicata           1    0    0    0    0    1    1    0    0    1    0    1
## Vertebrata         0    1    1    0    0    1    0    1    1    0    1    1
##                 M277 J221 M262 M279 J226 J53 J215 M117 M33 J47 J276 J52 M29
```

```
## Annelida           0    0    0    0    0   0    0    0   0   0    0   0   1
## Arthropoda         0    0    1    0    0   0    0    1   0   0    1   0   0
## Cnidaria           0    0    0    1    0   0    1    0   0   0    1   1   0
## Ctenophora         0    0    0    0    1   1    0    0   1   1    1   1   0
## Platyhelminthes    0    0    0    0    0   0    0    0   0   0    0   1   1
## Tunicata           0    0    0    1    0   1    1    0   0   1    0   1   1
## Vertebrata         1    1    1    0    0   1    0    1   0   0    1   1   0
##                 J138 J125 J121 M49 J144 J134 J150 J227 J57 M30 J46 J145 J96
## Annelida           0    0    0   0    0    0    0    0   0   0   0    0   0
## Arthropoda         0    0    1   1    0    0    0    0   0   1   1    1   0
## Cnidaria           0    1    0   1    0    1    1    1   1   0   0    1   1
## Ctenophora         1    0    0   0    1    1    1    0   1   0   1    0   1
## Platyhelminthes    0    0    0   1    0    0    0    1   0   0   0    0   0
## Tunicata           1    0    1   1    0    0    0    0   0   0   0    0   0
## Vertebrata         1    1    0   1    1    1    1    1   1   1   1    1   1
##                 J164 M123 M253 J229 J217 J205
## Annelida           0    0    0    0    1    0
## Arthropoda         0    0    1    1    0    0
## Cnidaria           1    0    1    0    0    0
## Ctenophora         1    1    0    0    0    0
## Platyhelminthes    0    0    0    1    0    0
## Tunicata           1    0    1    0    1    1
## Vertebrata         0    1    1    0    1    1
```

```
tPA_Tuna18S_D<-t(PA_Tuna18S_D)
tPA_TunaCOI_D<-t(PA_TunaCOI_D)
df5 <- cbind(dm2,tPA_Tuna18S_D)
df5$Year<-as.factor(df5$Year)
df6 <- cbind(dm,tPA_TunaCOI_D)
df6$Year<-as.factor(df6$Year)
df5$log.body.mass <- log10(df5$Weight)
df5$log.Length <- log10(df5$Longueur_Bouche)
df6$log.body.mass <- log10(df6$Weight)
df6$log.Length <- log10(df6$Longueur_Bouche)

#18S
modelCNI <- glm(Cnidaria ~ (Year+log.body.mass+log.Length), family = binomial, data = df5)
modelCNI02 <- step(modelCNI, direction = "both")
```

```
## Start:  AIC=70.06
## Cnidaria ~ (Year + log.body.mass + log.Length)
##
##                 Df Deviance    AIC
## - Year           3   58.540 64.540
## - log.Length     1   58.194 68.194
## - log.body.mass  1   58.658 68.658
## <none>               58.061 70.061
##
## Step:  AIC=64.54
## Cnidaria ~ log.body.mass + log.Length
##
##                 Df Deviance    AIC
## - log.Length     1   58.555 62.555
## - log.body.mass  1   59.327 63.327
```

```
## <none>               58.540 64.540
## + Year           3   58.061 70.061
##
## Step:  AIC=62.56
## Cnidaria ~ log.body.mass
##
##                 Df Deviance    AIC
## <none>               58.555 62.555
## - log.body.mass  1   60.633 62.633
## + log.Length     1   58.540 64.540
## + Year           3   58.194 68.194
```

```
modelCNI03 <- glm(Cnidaria~ 1,family = binomial, data = df5)
modelCNI04 <- step(modelCNI03
                   , direction = "both", scope = list(lower = ~1,upper = ~(Year+log.body.mass+log.Lengt
```

```
## Start:  AIC=62.63
## Cnidaria ~ 1
##
##                 Df Deviance    AIC
## + log.body.mass  1   58.555 62.555
## <none>               60.633 62.633
## + log.Length     1   59.327 63.327
## + Year           3   59.340 67.340
##
## Step:  AIC=62.56
## Cnidaria ~ log.body.mass
##
##                 Df Deviance    AIC
## <none>               58.555 62.555
## - log.body.mass  1   60.633 62.633
## + log.Length     1   58.540 64.540
## + Year           3   58.194 68.194
```

```
AIC(modelCNI,modelCNI02,modelCNI03,modelCNI04)
```

```
##            df      AIC
## modelCNI    6 70.06078
## modelCNI02  2 62.55541
## modelCNI03  1 62.63281
## modelCNI04  2 62.55541
```

```
summary(modelCNI, test="Chisq")
```

```
##
## Call:
## glm(formula = Cnidaria ~ (Year + log.body.mass + log.Length),
##     family = binomial, data = df5)
##
## Deviance Residuals:
##     Min       1Q   Median       3Q      Max
## -1.7111  -1.0310  -0.8776   1.2323   1.4846
```

```
##
## Coefficients:
##                Estimate Std. Error z value Pr(>|z|)
## (Intercept)     -1.5498    10.6781  -0.145    0.885
## Year2012         0.9303     2.0428   0.455    0.649
## Year2013         0.5372     1.9628   0.274    0.784
## Year2014         1.0924     2.1330   0.512    0.609
## log.body.mass    5.8629     7.6532   0.766    0.444
## log.Length      -7.1420    19.5955  -0.364    0.716
##
## (Dispersion parameter for binomial family taken to be 1)
##
##     Null deviance: 60.633  on 43  degrees of freedom
## Residual deviance: 58.061  on 38  degrees of freedom
## AIC: 70.061
##
## Number of Fisher Scoring iterations: 4
```

```
modelCTE <- glm(Ctenophora ~ (Year+log.body.mass+log.Length), family = binomial, data = df5)
modelCTE02 <- step(modelCTE, direction = "both")
```

```
## Start:  AIC=54.45
## Ctenophora ~ (Year + log.body.mass + log.Length)
##
##                 Df Deviance    AIC
## <none>               42.446 54.446
## - Year           3   48.976 54.976
## - log.Length     1   46.303 56.303
## - log.body.mass  1   49.345 59.345
```

```
modelCTE03 <- glm(Ctenophora~ 1,family = binomial, data = df5)
modelCTE04 <- step(modelCTE03
                   , direction = "both", scope = list(lower = ~1,upper = ~(Year+log.body.mass+log.Lengt
```

```
## Start:  AIC=59.68
## Ctenophora ~ 1
##
##                 Df Deviance    AIC
## + log.body.mass  1   50.517 54.517
## + Year           3   50.883 58.883
## <none>               57.682 59.682
## + log.Length     1   56.251 60.251
##
## Step:  AIC=54.52
## Ctenophora ~ log.body.mass
##
##                 Df Deviance    AIC
## <none>               50.517 54.517
## + log.Length     1   48.976 54.976
## + Year           3   46.303 56.303
## - log.body.mass  1   57.682 59.682
```

```r
AIC(modelCTE,modelCTE02,modelCTE03,modelCTE04)
```

```
##            df      AIC
## modelCTE    6 54.44555
## modelCTE02  6 54.44555
## modelCTE03  1 59.68240
## modelCTE04  2 54.51744
```

```r
summary(modelCTE, test="Chisq")
```

```
##
## Call:
## glm(formula = Ctenophora ~ (Year + log.body.mass + log.Length),
##     family = binomial, data = df5)
##
## Deviance Residuals:
##     Min       1Q   Median       3Q      Max
## -1.8862  -0.8674  -0.4349   0.8116   1.9476
##
## Coefficients:
##                Estimate Std. Error z value Pr(>|z|)
## (Intercept)      11.703     13.489   0.868   0.3856
## Year2012          2.485      2.806   0.886   0.3758
## Year2013          4.554      2.832   1.608   0.1078
## Year2014          4.858      2.918   1.665   0.0959 .
## log.body.mass    26.947     12.503   2.155   0.0311 *
## log.Length      -50.389     28.886  -1.744   0.0811 .
## ---
## Signif. codes:  0 '***' 0.001 '**' 0.01 '*' 0.05 '.' 0.1 ' ' 1
##
## (Dispersion parameter for binomial family taken to be 1)
##
##     Null deviance: 57.682  on 43  degrees of freedom
## Residual deviance: 42.446  on 38  degrees of freedom
## AIC: 54.446
##
## Number of Fisher Scoring iterations: 5
```

```r
modelART <- glm(Arthropoda~ (Year+log.body.mass+log.Length), family = binomial, data = df5)
modelART02 <- step(modelART, direction = "both")
```

```
## Start:  AIC=63.15
## Arthropoda ~ (Year + log.body.mass + log.Length)
##
##                 Df Deviance    AIC
## - Year           3   55.233 61.233
## <none>               51.153 63.153
## - log.body.mass  1   54.941 64.941
## - log.Length     1   55.540 65.540
##
## Step:  AIC=61.23
## Arthropoda ~ log.body.mass + log.Length
```

```
##
##                  Df Deviance    AIC
## - log.body.mass  1   56.008 60.008
## - log.Length     1   56.459 60.459
## <none>               55.233 61.233
## + Year           3   51.153 63.153
##
## Step:  AIC=60.01
## Arthropoda ~ log.Length
##
##                  Df Deviance    AIC
## - log.Length     1   56.464 58.464
## <none>               56.008 60.008
## + log.body.mass  1   55.233 61.233
## + Year           3   54.941 64.941
##
## Step:  AIC=58.46
## Arthropoda ~ 1
##
##                  Df Deviance    AIC
## <none>               56.464 58.464
## + log.Length     1   56.008 60.008
## + log.body.mass  1   56.459 60.459
## + Year           3   55.543 63.543
```

```
modelART03 <- glm(Arthropoda~ 1,family = binomial, data = df5)
modelART04 <- step(modelART03
                   , direction = "both", scope = list(lower = ~1,upper = ~(Year+log.body.mass+log.Lengt
```

```
## Start:  AIC=58.46
## Arthropoda ~ 1
##
##                  Df Deviance    AIC
## <none>               56.464 58.464
## + log.Length     1   56.008 60.008
## + log.body.mass  1   56.459 60.459
## + Year           3   55.543 63.543
```

```
AIC(modelART,modelART02,modelART03,modelART04)
```

```
##            df      AIC
## modelART    6 63.15254
## modelART02  1 58.46402
## modelART03  1 58.46402
## modelART04  1 58.46402
```

```
summary(modelART, test="Chisq")
```

```
##
## Call:
## glm(formula = Arthropoda ~ (Year + log.body.mass + log.Length),
##     family = binomial, data = df5)
```

```
##
## Deviance Residuals:
##     Min       1Q   Median       3Q      Max
## -1.4933  -0.9142  -0.6786   1.0691   1.8454
##
## Coefficients:
##               Estimate Std. Error z value Pr(>|z|)
## (Intercept)    -24.252     13.259  -1.829   0.0674 .
## Year2012        -3.649      2.396  -1.523   0.1278
## Year2013        -4.286      2.372  -1.806   0.0709 .
## Year2014        -4.374      2.571  -1.701   0.0890 .
## log.body.mass  -16.925      9.378  -1.805   0.0711 .
## log.Length      47.387     24.626   1.924   0.0543 .
## ---
## Signif. codes:  0 '***' 0.001 '**' 0.01 '*' 0.05 '.' 0.1 ' ' 1
##
## (Dispersion parameter for binomial family taken to be 1)
##
##     Null deviance: 56.464  on 43  degrees of freedom
## Residual deviance: 51.153  on 38  degrees of freedom
## AIC: 63.153
##
## Number of Fisher Scoring iterations: 4
```

```r
modelVER <- glm(Vertebrata ~ (Year+log.body.mass+log.Length), family = binomial, data = df5)
modelVER02 <- step(modelVER, direction = "both")
```

```
## Start:  AIC=62.04
## Vertebrata ~ (Year + log.body.mass + log.Length)
##
##                 Df Deviance    AIC
## - Year           3   52.020 58.020
## - log.body.mass  1   50.086 60.086
## - log.Length     1   51.120 61.120
## <none>               50.043 62.043
##
## Step:  AIC=58.02
## Vertebrata ~ log.body.mass + log.Length
##
##                 Df Deviance    AIC
## - log.Length     1   52.037 56.037
## - log.body.mass  1   53.504 57.504
## <none>               52.020 58.020
## + Year           3   50.043 62.043
##
## Step:  AIC=56.04
## Vertebrata ~ log.body.mass
##
##                 Df Deviance    AIC
## <none>               52.037 56.037
## - log.body.mass  1   55.043 57.043
## + log.Length     1   52.020 58.020
## + Year           3   51.120 61.120
```

```r
modelVER03 <- glm(Vertebrata~ 1,family = binomial, data = df5)
modelVER04 <- step(modelVER03
                   , direction = "both", scope = list(lower = ~1,upper = ~(Year+log.body.mass+log.Lengt
```

```
## Start:  AIC=57.04
## Vertebrata ~ 1
##
##                   Df Deviance    AIC
## + log.body.mass  1   52.037 56.037
## <none>                55.043 57.043
## + log.Length     1   53.504 57.504
## + Year           3   53.762 61.762
##
## Step:  AIC=56.04
## Vertebrata ~ log.body.mass
##
##                   Df Deviance    AIC
## <none>                52.037 56.037
## - log.body.mass  1   55.043 57.043
## + log.Length     1   52.020 58.020
## + Year           3   51.120 61.120
```

```r
AIC(modelVER,modelVER02,modelVER03,modelVER04)
```

```
##            df      AIC
## modelVER    6 62.04323
## modelVER02  2 56.03721
## modelVER03  1 57.04324
## modelVER04  2 56.03721
```

```r
summary(modelVER, test="Chisq")
```

```
##
## Call:
## glm(formula = Vertebrata ~ (Year + log.body.mass + log.Length),
##     family = binomial, data = df5)
##
## Deviance Residuals:
##     Min       1Q   Median       3Q      Max
## -2.1311  -1.0603   0.5821   0.8881   1.2635
##
## Coefficients:
##                 Estimate Std. Error z value Pr(>|z|)
## (Intercept)     -18.390     12.107  -1.519    0.129
## Year2012         -2.667      2.263  -1.178    0.239
## Year2013         -1.729      2.186  -0.791    0.429
## Year2014         -2.352      2.342  -1.004    0.315
## log.body.mass    -1.757      8.467  -0.207    0.836
## log.Length       21.931     21.360   1.027    0.305
##
## (Dispersion parameter for binomial family taken to be 1)
##
```

```
##     Null deviance: 55.043  on 43  degrees of freedom
## Residual deviance: 50.043  on 38  degrees of freedom
## AIC: 62.043
##
## Number of Fisher Scoring iterations: 4
```

```
modelTUN <- glm(Tunicata ~ (Year+log.body.mass+log.Length), family = binomial, data = df5)
modelTUN02 <- step(modelTUN, direction = "both")
```

```
## Start:  AIC=63.92
## Tunicata ~ (Year + log.body.mass + log.Length)
##
##                 Df Deviance    AIC
## - Year           3   55.933 61.933
## - log.body.mass  1   52.522 62.522
## - log.Length     1   52.603 62.603
## <none>               51.923 63.923
##
## Step:  AIC=61.93
## Tunicata ~ log.body.mass + log.Length
##
##                 Df Deviance    AIC
## - log.body.mass  1   56.186 60.186
## <none>               55.933 61.933
## - log.Length     1   58.661 62.661
## + Year           3   51.923 63.923
##
## Step:  AIC=60.19
## Tunicata ~ log.Length
##
##                 Df Deviance    AIC
## <none>               56.186 60.186
## - log.Length     1   59.534 61.534
## + log.body.mass  1   55.933 61.933
## + Year           3   52.522 62.522
```

```
modelTUN03 <- glm(Tunicata~ 1,family = binomial, data = df5)
modelTUN04 <- step(modelTUN03
                 , direction = "both", scope = list(lower = ~1,upper = ~(Year+log.body.mass+log.Length))
```

```
## Start:  AIC=61.53
## Tunicata ~ 1
##
##                 Df Deviance    AIC
## + log.Length     1   56.186 60.186
## + Year           3   52.610 60.610
## <none>               59.534 61.534
## + log.body.mass  1   58.661 62.661
##
## Step:  AIC=60.19
## Tunicata ~ log.Length
##
##                 Df Deviance    AIC
```

```
## <none>                56.186 60.186
## - log.Length     1    59.534 61.534
## + log.body.mass  1    55.933 61.933
## + Year           3    52.522 62.522
```

```r
AIC(modelTUN,modelTUN02,modelTUN03,modelTUN04)
```

```
##            df      AIC
## modelTUN    6 63.92257
## modelTUN02  2 60.18589
## modelTUN03  1 61.53428
## modelTUN04  2 60.18589
```

```r
summary(modelTUN, test="Chisq")
```

```
##
## Call:
## glm(formula = Tunicata ~ (Year + log.body.mass + log.Length),
##     family = binomial, data = df5)
##
## Deviance Residuals:
##     Min       1Q   Median       3Q      Max
## -1.5941  -0.9816  -0.6203   0.9335   1.9846
##
## Coefficients:
##               Estimate Std. Error z value Pr(>|z|)
## (Intercept)     -7.588     12.068  -0.629   0.5295
## Year2012        -2.697      2.268  -1.189   0.2344
## Year2013        -2.993      2.187  -1.369   0.1711
## Year2014        -4.185      2.483  -1.686   0.0919 .
## log.body.mass   -6.351      8.306  -0.765   0.4445
## log.Length      17.510     21.490   0.815   0.4152
## ---
## Signif. codes:  0 '***' 0.001 '**' 0.01 '*' 0.05 '.' 0.1 ' ' 1
##
## (Dispersion parameter for binomial family taken to be 1)
##
##     Null deviance: 59.534  on 43  degrees of freedom
## Residual deviance: 51.923  on 38  degrees of freedom
## AIC: 63.923
##
## Number of Fisher Scoring iterations: 4
```

```r
# same for COI

modelan01 <- glm(anchovy~ (Year+log.body.mass+log.Length), family = binomial, data = df6)
modelan02 <- step(modelan01, direction = "both")
```

```
## Start:  AIC=43.53
## anchovy ~ (Year + log.body.mass + log.Length)
##
##                   Df Deviance    AIC
```

```
## - Year            3   37.163 43.163
## <none>                31.534 43.534
## - log.Length      1   33.549 43.549
## - log.body.mass   1   37.521 47.521
##
## Step:  AIC=43.16
## anchovy ~ log.body.mass + log.Length
##
##                  Df Deviance    AIC
## - log.Length      1   37.203 41.203
## <none>                37.163 43.163
## + Year            3   31.534 43.534
## - log.body.mass   1   41.110 45.110
##
## Step:  AIC=41.2
## anchovy ~ log.body.mass
##
##                  Df Deviance    AIC
## <none>                37.203 41.203
## + log.Length      1   37.163 43.163
## + Year            3   33.549 43.549
## - log.body.mass   1   42.121 44.121
```

```
modelan03 <- glm(anchovy~ 1,family = binomial, data = df6)
modelan04 <- step(modelan03
                  , direction = "both", scope = list(lower = ~1,upper = ~(Year+log.body.mass+log.Length
```

```
## Start:  AIC=44.12
## anchovy ~ 1
##
##                  Df Deviance    AIC
## + log.body.mass   1   37.203 41.203
## <none>                42.121 44.121
## + log.Length      1   41.110 45.110
## + Year            3   40.819 48.819
##
## Step:  AIC=41.2
## anchovy ~ log.body.mass
##
##                  Df Deviance    AIC
## <none>                37.203 41.203
## + log.Length      1   37.163 43.163
## + Year            3   33.549 43.549
## - log.body.mass   1   42.121 44.121
```

```
AIC(modelan01,modelan02,modelan03,modelan04)
```

```
##           df      AIC
## modelan01  6 43.53367
## modelan02  2 41.20300
## modelan03  1 44.12063
## modelan04  2 41.20300
```

```r
summary(modelan01, test="Chisq")
```

```
##
## Call:
## glm(formula = anchovy ~ (Year + log.body.mass + log.Length),
##     family = binomial, data = df6)
##
## Deviance Residuals:
##      Min        1Q    Median        3Q       Max
## -2.46190   0.07094   0.39318   0.52515   1.35065
##
## Coefficients:
##               Estimate Std. Error z value Pr(>|z|)
## (Intercept)     -1.235     16.366  -0.075   0.9399
## Year2012         4.452      3.196   1.393   0.1636
## Year2013         5.056      2.994   1.689   0.0912 .
## Year2014         2.729      2.916   0.936   0.3493
## log.body.mass   30.178     14.363   2.101   0.0356 *
## log.Length     -39.844     29.631  -1.345   0.1787
## ---
## Signif. codes:  0 '***' 0.001 '**' 0.01 '*' 0.05 '.' 0.1 ' ' 1
##
## (Dispersion parameter for binomial family taken to be 1)
##
##     Null deviance: 42.121  on 44  degrees of freedom
## Residual deviance: 31.534  on 39  degrees of freedom
## AIC: 43.534
##
## Number of Fisher Scoring iterations: 6
```

```r
modelar01 <- glm(Arthropoda~ (Year+log.body.mass+log.Length), family = binomial, data = df6)
modelar02 <- step(modelar01, direction = "both")
```

```
## Start:  AIC=58.96
## Arthropoda ~ (Year + log.body.mass + log.Length)
##
##                 Df Deviance    AIC
## - Year           3   48.982 54.982
## - log.Length     1   47.104 57.104
## - log.body.mass  1   48.406 58.406
## <none>               46.963 58.963
##
## Step:  AIC=54.98
## Arthropoda ~ log.body.mass + log.Length
##
##                 Df Deviance    AIC
## - log.Length     1   48.983 52.983
## - log.body.mass  1   50.878 54.878
## <none>               48.982 54.982
## + Year           3   46.963 58.963
##
## Step:  AIC=52.98
```

```
## Arthropoda ~ log.body.mass
##
##                 Df Deviance    AIC
## <none>               48.983 52.983
## - log.body.mass  1   52.192 54.192
## + log.Length     1   48.982 54.982
## + Year           3   47.104 57.104
```

```r
modelar03 <- glm(Arthropoda~ 1,family = binomial, data = df6)
modelar04 <- step(modelar03
                  , direction = "both", scope = list(lower = ~1,upper = ~(Year+log.body.mass+log.Length)
```

```
## Start:  AIC=54.19
## Arthropoda ~ 1
##
##                 Df Deviance    AIC
## + log.body.mass  1   48.983 52.983
## <none>               52.192 54.192
## + log.Length     1   50.878 54.878
## + Year           3   50.947 58.947
##
## Step:  AIC=52.98
## Arthropoda ~ log.body.mass
##
##                 Df Deviance    AIC
## <none>               48.983 52.983
## - log.body.mass  1   52.192 54.192
## + log.Length     1   48.982 54.982
## + Year           3   47.104 57.104
```

```r
AIC(modelar01,modelar02,modelar03,modelar04)
```

```
##           df      AIC
## modelar01  6 58.96261
## modelar02  2 52.98265
## modelar03  1 54.19237
## modelar04  2 52.98265
```

```r
summary(modelar01, test="Chisq")
```

```
##
## Call:
## glm(formula = Arthropoda ~ (Year + log.body.mass + log.Length),
##     family = binomial, data = df6)
##
## Deviance Residuals:
##     Min       1Q   Median       3Q      Max
## -1.2187  -0.8124  -0.6083   0.9081   2.2695
##
## Coefficients:
##                 Estimate Std. Error z value Pr(>|z|)
## (Intercept)      6.3402    12.4061   0.511    0.609
```

```
## Year2012       -0.6547     2.3040  -0.284    0.776
## Year2013       -1.7461     2.2715  -0.769    0.442
## Year2014       -0.5027     2.3619  -0.213    0.831
## log.body.mass -11.0264     9.5105  -1.159    0.246
## log.Length      8.3316    22.2404   0.375    0.708
##
## (Dispersion parameter for binomial family taken to be 1)
##
##     Null deviance: 52.192  on 44  degrees of freedom
## Residual deviance: 46.963  on 39  degrees of freedom
## AIC: 58.963
##
## Number of Fisher Scoring iterations: 5
```

```
modelmr01 <- glm(mackerel~ (Year+log.body.mass+log.Length), family = binomial, data = df6)
modelmr02 <- step(modelmr01, direction = "both")
```

```
## Start:  AIC=63
## mackerel ~ (Year + log.body.mass + log.Length)
##
##                 Df Deviance    AIC
## - Year           3   52.752 58.752
## - log.Length     1   51.128 61.128
## - log.body.mass  1   51.162 61.162
## <none>               51.005 63.005
##
## Step:  AIC=58.75
## mackerel ~ log.body.mass + log.Length
##
##                 Df Deviance    AIC
## - log.Length     1   53.238 57.238
## <none>               52.752 58.752
## - log.body.mass  1   55.212 59.212
## + Year           3   51.005 63.005
##
## Step:  AIC=57.24
## mackerel ~ log.body.mass
##
##                 Df Deviance    AIC
## <none>               53.238 57.238
## - log.body.mass  1   55.799 57.799
## + log.Length     1   52.752 58.752
## + Year           3   51.128 61.128
```

```
modelmr03 <- glm(mackerel~ 1,family = binomial, data = df6)
modelmr04 <- step(modelmr03
                  , direction = "both", scope = list(lower = ~1,upper = ~(Year+log.body.mass+log.Length
```

```
## Start:  AIC=57.8
## mackerel ~ 1
##
##                 Df Deviance    AIC
## + log.body.mass  1   53.238 57.238
```

```
## <none>               55.799 57.799
## + log.Length     1   55.212 59.212
## + Year           3   53.971 61.971
##
## Step:  AIC=57.24
## mackerel ~ log.body.mass
##
##                 Df Deviance    AIC
## <none>               53.238 57.238
## - log.body.mass  1   55.799 57.799
## + log.Length     1   52.752 58.752
## + Year           3   51.128 61.128
```

```
AIC(modelmr01,modelmr02,modelmr03,modelmr04)
```

```
##           df      AIC
## modelmr01  6 63.00483
## modelmr02  2 57.23836
## modelmr03  1 57.79881
## modelmr04  2 57.23836
```

```
summary(modelmr01, test="Chisq")
```

```
##
## Call:
## glm(formula = mackerel ~ (Year + log.body.mass + log.Length),
##     family = binomial, data = df6)
##
## Deviance Residuals:
##     Min       1Q   Median       3Q      Max
## -1.2037  -0.8971  -0.6048   1.1929   2.2000
##
## Coefficients:
##                Estimate Std. Error z value Pr(>|z|)
## (Intercept)    -12.3754    12.3174  -1.005    0.315
## Year2012        -0.7957     2.1940  -0.363    0.717
## Year2013        -1.7617     2.1188  -0.831    0.406
## Year2014        -1.8070     2.4218  -0.746    0.456
## log.body.mass    3.2378     8.1481   0.397    0.691
## log.Length       7.4815    21.4473   0.349    0.727
##
## (Dispersion parameter for binomial family taken to be 1)
##
##     Null deviance: 55.799  on 44  degrees of freedom
## Residual deviance: 51.005  on 39  degrees of freedom
## AIC: 63.005
##
## Number of Fisher Scoring iterations: 4
```

```
modelsa01 <- glm(sardine~ (Year+log.body.mass+log.Length), family = binomial, data = df6)
modelsa02 <- step(modelsa01, direction = "both")
```

```
## Start:  AIC=67.8
## sardine ~ (Year + log.body.mass + log.Length)
##
##                 Df Deviance    AIC
## - Year           3   59.163 65.163
## - log.Length     1   55.803 65.803
## - log.body.mass  1   55.968 65.968
## <none>               55.795 67.795
##
## Step:  AIC=65.16
## sardine ~ log.body.mass + log.Length
##
##                 Df Deviance    AIC
## - log.body.mass  1   60.477 64.477
## <none>               59.163 65.163
## - log.Length     1   62.149 66.149
## + Year           3   55.795 67.795
##
## Step:  AIC=64.48
## sardine ~ log.Length
##
##                 Df Deviance    AIC
## - log.Length     1   62.183 64.183
## <none>               60.477 64.477
## + log.body.mass  1   59.163 65.163
## + Year           3   55.968 65.968
##
## Step:  AIC=64.18
## sardine ~ 1
##
##                 Df Deviance    AIC
## <none>               62.183 64.183
## + Year           3   56.435 64.435
## + log.Length     1   60.477 64.477
## + log.body.mass  1   62.149 66.149
```

```
modelsa03 <- glm(sardine~ 1,family = binomial, data = df6)
modelsa04 <- step(modelsa03
                  , direction = "both", scope = list(lower = ~1,upper = ~(Year+log.body.mass+log.Length)
```

```
## Start:  AIC=64.18
## sardine ~ 1
##
##                 Df Deviance    AIC
## <none>               62.183 64.183
## + Year           3   56.435 64.435
## + log.Length     1   60.477 64.477
## + log.body.mass  1   62.149 66.149
```

```
AIC(modelsa01,modelsa02,modelsa03,modelsa04)
```

```
##           df     AIC
## modelsa01  6 67.7953
```

```
## modelsa02  1 64.1831
## modelsa03  1 64.1831
## modelsa04  1 64.1831
```

```
summary(modelsa01, test="Chisq")
```

```
##
## Call:
## glm(formula = sardine ~ (Year + log.body.mass + log.Length),
##     family = binomial, data = df6)
##
## Deviance Residuals:
##     Min       1Q   Median       3Q      Max
## -1.9265  -1.0544   0.6229   0.9368   1.6033
##
## Coefficients:
##               Estimate Std. Error z value Pr(>|z|)
## (Intercept)      2.038     11.556   0.176    0.860
## Year2012         1.217      2.157   0.564    0.573
## Year2013         0.290      2.053   0.141    0.888
## Year2014         2.114      2.317   0.912    0.362
## log.body.mass   -3.328      8.049  -0.414    0.679
## log.Length       1.797     20.827   0.086    0.931
##
## (Dispersion parameter for binomial family taken to be 1)
##
##     Null deviance: 62.183  on 44  degrees of freedom
## Residual deviance: 55.795  on 39  degrees of freedom
## AIC: 67.795
##
## Number of Fisher Scoring iterations: 4
```

```
modelsp01 <- glm(sprat~ (Year+log.body.mass+log.Length), family = binomial, data = df6)
modelsp02 <- step(modelsp01, direction = "both")
```

```
## Start:  AIC=67.41
## sprat ~ (Year + log.body.mass + log.Length)
##
##                 Df Deviance    AIC
## - Year           3   56.828 62.828
## - log.body.mass  1   55.483 65.483
## - log.Length     1   55.918 65.918
## <none>               55.414 67.414
##
## Step:  AIC=62.83
## sprat ~ log.body.mass + log.Length
##
##                 Df Deviance    AIC
## - log.body.mass  1   57.168 61.168
## - log.Length     1   57.905 61.905
## <none>               56.828 62.828
## + Year           3   55.414 67.414
##
```

```
## Step:  AIC=61.17
## sprat ~ log.Length
##
##                  Df Deviance    AIC
## <none>               57.168 61.168
## + log.body.mass  1   56.828 62.828
## - log.Length     1   61.827 63.827
## + Year           3   55.483 65.483
```

```
modelsp03 <- glm(sprat~ 1,family = binomial, data = df6)
modelsp04 <- step(modelsp03
                  , direction = "both", scope = list(lower = ~1,upper = ~(Year+log.body.mass+log.Length)
```

```
## Start:  AIC=63.83
## sprat ~ 1
##
##                  Df Deviance    AIC
## + log.Length     1   57.168 61.168
## + log.body.mass  1   57.905 61.905
## <none>               61.827 63.827
## + Year           3   60.312 68.312
##
## Step:  AIC=61.17
## sprat ~ log.Length
##
##                  Df Deviance    AIC
## <none>               57.168 61.168
## + log.body.mass  1   56.828 62.828
## - log.Length     1   61.827 63.827
## + Year           3   55.483 65.483
```

```
AIC(modelsp01,modelsp02,modelsp03,modelsp04)
```

```
##           df      AIC
## modelsp01  6 67.41386
## modelsp02  2 61.16769
## modelsp03  1 63.82654
## modelsp04  2 61.16769
```

```
summary(modelsp01, test="Chisq")
```

```
##
## Call:
## glm(formula = sprat ~ (Year + log.body.mass + log.Length), family = binomial,
##     data = df6)
##
## Deviance Residuals:
##     Min       1Q   Median       3Q      Max
## -1.4381  -1.0623  -0.6026   1.0925   1.7905
##
## Coefficients:
##              Estimate Std. Error z value Pr(>|z|)
```

```
## (Intercept)   -18.6381    12.5318  -1.487    0.137
## Year2012       -0.1934     2.1366  -0.091    0.928
## Year2013       -0.6075     2.0433  -0.297    0.766
## Year2014       -1.4233     2.3606  -0.603    0.547
## log.body.mass   2.1234     8.0662   0.263    0.792
## log.Length     14.8976    21.2226   0.702    0.483
##
## (Dispersion parameter for binomial family taken to be 1)
##
##     Null deviance: 61.827  on 44  degrees of freedom
## Residual deviance: 55.414  on 39  degrees of freedom
## AIC: 67.414
##
## Number of Fisher Scoring iterations: 4
```

```
### make drawing
line.length <- 1000
df5$Year<-as.factor(df5$Year)

plot(df5$log.body.mass, df5$Ctenophora, type="n", xlab="", ylab = "")
modelan <- glm(anchovy~ (Year+log.body.mass+log.Length), family = binomial, data = df6)
x.anchovy.1 <- seq(min(df6$log.body.mass),max(df6$log.body.mass),length=line.length)
y.anchovy.1  <- predict(modelan, list(log.body.mass=x.anchovy.1, log.Length=rep(mean(df5$log.Length),10
                                      Year=as.factor(rep("2012",1000))), type="response")
rug(jitter(df6$log.body.mass[df6$anchovy==0]), col = "navy")
rug(jitter(df6$log.body.mass[df6$anchovy==0]), col = "navy", line=-0.06)
rug(jitter(df6$log.body.mass[df6$anchovy==1]),side=3, col="navy")
rug(jitter(df6$log.body.mass[df6$anchovy==1]),side=3, col="navy",line=-0.06)
lines(x.anchovy.1,y.anchovy.1,type="l", col="navy", lwd=2)


modelCTE <- glm(Ctenophora ~(Year+log.body.mass+log.Length), family = binomial, data = df5)
x.Ctenophora.1 <- seq(min(df5$log.body.mass),max(df5$log.body.mass),length=line.length)
y.Ctenophora.1  <- predict(modelCTE , list(log.body.mass=x.Ctenophora.1,
                                           log.Length=rep(mean(df5$log.Length),1000),Year=as.factor(rep
rug(jitter(df5$log.body.mass[df5$Ctenophora==0]), col = "aquamarine2",line=-0.02)
rug(jitter(df5$log.body.mass[df5$Ctenophora==1]),side=3, col="aquamarine2", line=-0.02)
lines(x.Ctenophora.1,y.Ctenophora.1,type="l", col="aquamarine2", lwd=2)
```

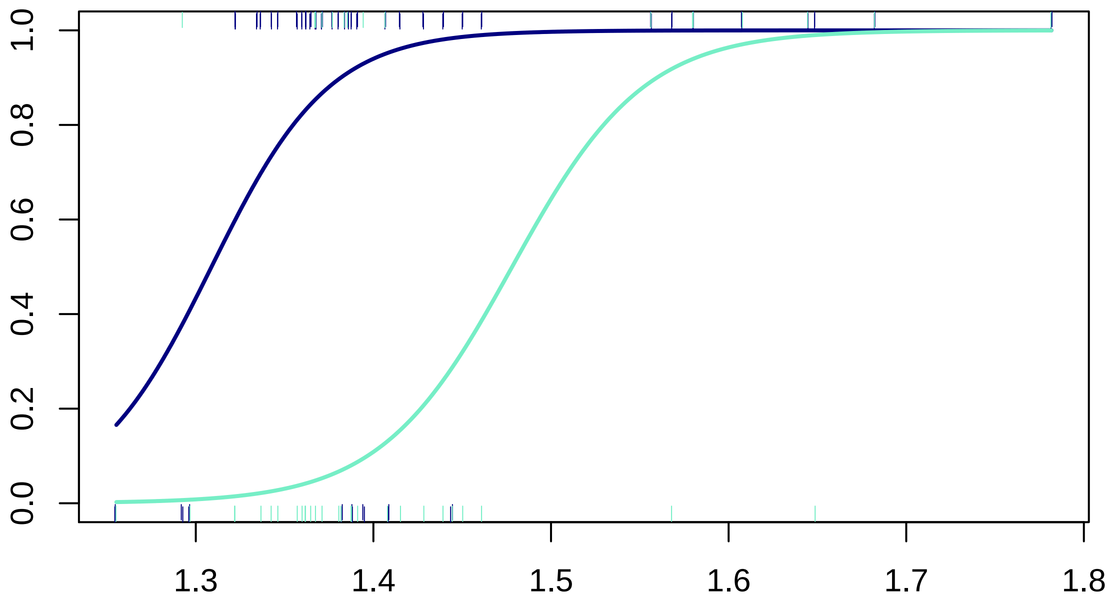
1.0
0.8
0.6
0.4
0.2
0.0
1.3
1.4
1.5
1.6
1.7
1.8
